# 

**Published:** 1874-12

**Authors:** 


### NOTICE.

With pleasure we give place to the following from Dr. Goddard, Treasurer of the American Dental Association.

An opportunity is now offered to those who first apply to procure the Transactions without money and without price.

"At the last meeting of the American Dental Association, held at Detroit, Mich., a resolution was adopted instructing the Treasurer to dispose of the back numbers of their Transactions for the cost of mailing.

I hereby give notice that the Transactions of 1865—66, one volume, can be had by applying to Dr. L. D. Shepard, Hotel Boylston, Boston, Mass., and sending twenty-five cents.

For 1867, of Dr. Wm. H. Goddard, Louisville, Ky., for fifteen cents.

For 1868, of A. M. Leslie and Co., St. Louis, Mo., for fifteen cents.

For 1871—72, one volume, of M. S. Dean, Chicago, Ills., for twenty cents.

For 1873, of Dr. Dean, bound in paper, for 50 cts.

The Transactions of 1869 were consumed in the great fire in Chicago, Ills.

Persons wishing any of the above will address the parties above mentioned. None will be sent without the amount specified accompanying the request.

WM. H. GODDARD, Treas.,
65 W. Walnut st., Louisville, Ky.

### THE PHYSICIAN'S VISITING LIST FOR 1875.

This is a well arranged engagement and memorandum book prepared for the use of physicians, by Lindsay and Blakiston, of Philadelphia. It contains all that is required for the profession.

It is furnished by booksellers generally.

AMERICAN DENTAL ASSOCIATION—Meets on the first Tuesday in Aug., 1874, at Detroit. *Pres.* T. L. Buckingham, Philad.; *Cor. Sec.* J. Taft, Cincinnati, O.; *Rec. Sec.* M. S. Dean, Chicago.

*American Dental Convention* Meets at Saratoga Springs, second Tuesday in Aug., 1874. *Pres.* John Allen, New York City; *Cor. Sec.* A. Tees, Philadelphia, Pa., *Rec. Sec.* C. L. Hurlbut, Springfield, Mass.

*List of Societies Represented in the American Dental Association.*

*American Academy of Dental Science*Meets at Boston, Mass. *Pres.* DANIEL HARWOOD, M. D., of Boston; *Sec.* L. D. Shepard, D. D. S., of Boston; *Cor. Sec.*, E. N. HARRIS.

*Brooklyn Dental Society* Meets semi-monthly. *Pres.* W. T. Shannon,; *Rec. Sec.* C. P. Crandell; *Cor. Sec.* A. N. Chapman.

*Buffalo Dental Society*Meets second Monday of each month at Buffalo. *Pres.* S. A. FREEMAN; *Rec. Sec.* G. C. DABOLL.

*Boston Dental Institute*—Meets first Tuesday of each month. *Pres.* D. G. WILLIAMS; *Cor. Sec.* D. S. DICKERMAN.

*California State Dental Association* Meets annually in June. *Pres.* C. C. Knowles, San Francisco; *Rec. Sec.* H. J. Plomteaux, Woodland.

*Cincinnati Dental Society*—Meets Semi-monthly at Cincinnati. *Pres.* H. R. SMITH; *Rec. Sec.* D. C. HAWXHURST.

*Chicago Dental Society* Meets monthly at Chicago. *Pres* I. N. Crouse; *Rec. Sec.* Edgar D. Swain.

*Connecticut Valley Dental Association.* Meets semi-annually, second Tuesday of May and October. *Pres.* A. A. Howland, Barre, Vt.; *Rec. Sec.* W. H. Jones, of Boston, Mass.

*Connecticut State Dental Association.* *Pres.* R. C. Dunham, New Britain; *Rec. Sec.* D. E. Lane, Hartford.

*Charleston Dental Association*—Meets second Tuesday of every month; *Pres.* T. F. Chupein; *Sec. and Treas.* B. A. Muckenfuss.

*Delaware Dental Association*—Meets semi-annually. *Pres.* E. W. Haines, Newark; *Sec.* C. R. Jeffries, Wilmington.

*Eastern Ohio Dental Association*—Meets on Tuesday, August 26th, 1873, at Alliance; *Pres.* A. J. Douds; *Sec.* J. M. Porter.

*East Tennessee Dental Association*—Meets annually at Knoxville, Tenn. *Pres.* Wm. H. Cooke, of Cleveland; *Rec. Sec.* A. P. White, Knoxville.

*Forest City Society of Dental Surgeons*—*Pres.* B. Strickland, Cleveland, *Rec. Sec.* H. L. Ambler, Cleveland; *Cor. Sec.* Chas. Buffett, Cleveland.

*Georgia State Dental Society* Next meeting at Atlanta, July 1873. *Pres.* F. Y. Clark; *Cor. Sec.* J. P. H. Brown, Augusta.

*Harris Dental Association* Meets quarterly on the first Thursday in February, May, August, November. *Pres.* J. A. Martin, Strasburg; *Sec.* Wm. N. Amer, Lancaster,

*Hudson River Association of Dental Surgeons* Meets on the second Wednesdays of May, September and January. *Pres.* L. S. Straw, Newburg, N. Y; *Rec. Sec.* T. W. DuBois, Poughkeepsie, N. Y.

*Hudson Valley Dental Association* Meets monthly at Troy, N. Y. *Pres.* L. C. Wheeler, *Rec. Sec.* S. G. Andres; *Cor. Sec.* S. D. French.

*Illinois State Dental Society* Meets annually. *Pres.* C. Stoddard Smith, Springfield; *Sec.* C. R. E. Koch, Chicago. Meets second Tuesday in May, 1874, at Jacksonville.

*Indiana State Dental Society*—Meets next year at Indianapolis, on the first Tuesday of June. *Pres.* M. Wells, Indianapolis; *Cor. and Rec. Sec.* P. McDonald, Goshen.

*Iowa State Dental Society*—Meets annually. Next meeting at Davenport, on the second Tuesday of May. *Pres.* L. C. Ingersoll, Keokuk. *Rec. Sec.* E. P. White, of Davenport; *Cor. Sec.* A. J. Redrick, Sioux City.

*Kansas State Dental Society* Meets semi-annually. Next meeting at Topeka, Sept. 12, 1873. *Pres.* J. B. Wheeler; *Sec.* E. C. Fuller.

*Kansas and Missouri Valley Dental Association*—Meets first and third Tuesdays of erch month. *Pres.* J. R.Stark; *Sec.* H. C. Massie, Kansas City, Mo.

*Kentucky State Dental Association* Meets annually and semi-monthly, in the City of Louisville. Annnal meeting, first Tuesday in June, at 2 o'clock, P. M. Semi-monthly meetings, second and fourth Tuesday nights. G. W. Priest, *Pres.*; B. O. Doyle. *Sec.*, both at Louisville.

*Lake Erie Dental Association* Meets Annually. *Pres.* C. B. Ansart, Oil City, Pa.; *Sec.* C. D. Elliot, Franklin, Pa. Next meeting first Tuesday of May, 1874.

*Mad River Dental Association* Meets at Dayton on the first Tuesdays in April and October. *Pres.* G. W. Keely, Oxford; *Sec.* J. A Stipp, Sidney.

*Maine Dental Society*—Meets in Aug., at Thomaston. *Pres.* E. J. Roberts, Augusta; *Sec.* C. E. Hussey, Biddeford.

*Maryland Association of Dentists* Meets the last Thursday of every month. *Pres.* J. B. Bean; *Sec.* S. M. Field.

*Massachusetts Dental Society*—Meets monthly. *Pres.* J. H. Batchelder, of Salem; *Sec.* C. Wilson; *Cor. Sec.* L. D. Shepard.

*Michigan State Dental Association*—Meets annually second Tuesday of Oct., at Ypsilanti. *Pres.* D. C. Hawxhurst, Ann Arbor; *Rec. & Cor. Sec.* T. R. Perry, Grand Rapids

*Michigan Central Dental Association.* Meets annually at Jackson, Secon Wednesday of January, 1873. *Pres.* C. C. Jenkins, Anu Arbor, *Sec.* W. H. Darrance, Jackson.

*Mississippi Valley Dental Association* Meets annually at St. Louis, on first Wednesday in March, 1874. *Pres.* W. H. Eames, St. Louis, Mo.; *Rec. Sec.* F. Hunter Cin.

*Minnesota Dental Associrtion* Meets annually. Next meeting at Faribault, last Wednesday in June, 1874. *Pres.* J. A. Bowman, Minneapolis; *Sec.* C. D. Montreville, St. Paul.

*Missouri Dental Association* Meets on the first Tuesday of June, in St. Joseph. *Pres.* J. K. Stark, Kansas City; *Sec.* W. H. Eames, St. Louis.

*Missouri Valley Dental Association** Meets on the second Tuesday in July and January. *Pres.* A. S. Billings, Omaha, Neb.; *Sec. and Treas.* J. F. Sanborn, Tabor, Iowa.

*Merrimac Valley Dental Association* Meets semi-annually first Tuesday of Oct. and May.* *Pres.* L. H. Kidder, Mass; *Rec. Sec.* A. M. Dudley, of Lowell; *Cor. Sec.* D. T. Porter, Lawrence, Mass.

*Memphis Dental Society*—Meets on the first Tuesday of each month. *Pres.* W. M. Arrington; *Sec.* V. F. Elliott; *Treas.* C. L. Harris.

*Nashville Dental Association*—Meets on the second Tuesday of each month. *Pres* J. C. Ross; *Sec.* R. R. Freeman; *Cor. Sec.* S. J. Cobb.

*New Jersey State Dental Society*—Meets annually. Next meeting at Mount Holly, on the second Tuesday in July, 1874. *Pres.* J. W. Cosad, Jersey City; *Sec.* J. W. Scarborough, Lambertville.

*New York State Dental Society*--Meets at Albany on the last Wednesday of June next. *Pres.* J. G. Ambler, New Yook; *Sec.* Chas. Barnes, of Syracuse, *Cor. Sec.* S. A. Freeman.

*New Haven Dental Society*--Meets on the first Wednesday of each month at New Haven. *Pres.* J. T. Metcalf, *Rec and Cor. Sec.* C. L. Smith, of New Haven.

*New Orleans Dental Society* Meets monthly. *Pres.*————————; *Rec. Sec.* A. F McLane.

*Northern Ohio Dental Association.*Meets annually, second Tuesday of June, 1873, at Put-in Bay. *Pres.* J. A. Robinson, Cleveland; *Rec. Sec.* A. F. Price, Fremont.

*Northern Iowa Dental Association*—Meets annually, on the second Tuesday in June, 1873. Next meeting at Waterloo; *Pres.* J. N. Abbott, Lancaster, *Rec. Sec.* A. V. Eaton, Anamosa; *Cor. Sec.* A. R. Mason, Waterloo.

*Ohio College Dental Association*--Meets annually in March, at Cincinnati. *Pres.* W. H. Goddard, Louisville, Ky.; *Rec. Sec.* J. Taft, of Cincinnati.

*Ohio State Dental Association*--Meets annually at Columbus. Next meeting, first Wednesday of December. *Pres.* J. M. Porter, Massilon; *Rec. Sec.* H. A. Smith Cincinnati, O.; *Cor. Sec.* C. R. Butler, Cleveland, O.

*Odontographic Society of Pennsylvania*--Meets monthly in Philadelphia. Next meeting, Wednesday, Sept. 1st. *Pres.* C. T. Stellwagen; *Rec. Sec.* A. Boice; *Cor. Sec.* W. F. Litch.

*Odontological Society of New York City* Meets the second Tuesday of each monh. *Pres.* C. E. Francis; *Rec. Sec.* W. E. Hong.

*Old Colony Dental Association* Meets quarterly.* *Pres.* C. G. Davis; *Rec. Sec.* L. W. Puffer, North Bridgewater, Mass; *Cor. Sec.* N. C. Fowler, Yarmouth, Mass.

*Ohio Valley Dental Association* Meets annually, in N. E. Ky; this year in Paris, Ky. Tuesday, Sept. 9th.

*Oregon State Dental Society* Meets annually. *Pres.* J. H. Hatch, Portland; *Rec. sec.* J. R. Cardwell, Portland.

*Pennsylvania State Dental Society* Meets next at Wilkesbarre, second Tuesday of July, 1874. *Pres.* G. W. Klump, Williamsport; *Rec. Sec.* R. H. Moffit, Harrisburg *Cor. Sec.* S. Welchens, Lancaster.

*Poughkeepsie Dental Society* Meets monthly. *Pres.* J. H. Mann; *Sec.* H. F. Clark

*San Francisco Dental Association* Meets monthly. *Pres.* C. C. Knowles; *Cor. Sec.* H. E. Knox; *Rec. Sec.* Wm. Dutch.

*St. Louis Dental Society* Meets monthly. *Pres.* W. H. Morrison; *Sec.* A. H. Fuller.

*Susquehanna Dental Association* Meets annually.* *Pres.* J. L. Fordham: *Rec. Sec.* G. W. Klump, Williamsport. Meets Nov. 12, 1873.

*Society of Dental Surgeons of the City of New York* Meets semi-monthly in New York. *Pres.* B. F. Franklin: *Rec. Sec.* J. S. Latimer; *Cor. Sec.* E. H. Burras.

**Southern Dental Association* Meets annually. Next meeting the last Tuesday of July, 1873, at Baltimore, Md. *Pres.* H. M. Grant Virginia.: *Rec. Sec.* J. F. Thompson Va

*South Carolina State Dental Association* Meets semi-annually in May and Nov. *Pres.* W. C. Wardlaw, Abbeville: *Sec.* O. J. Bond, Marion.

*Tennessee Dental Association* Meets semi-annually. Next meeting in Nashville, on the 4th Wednesday in June. 1874. *Pres.* R. Russell, Nashville: *Sec.* A. Hartman, Murfreesboro; *Cor. Sec.* L. G. Noel, Nashville.

*Virginia State Dental Association* Meets in Richmond, first Thursday in Nov. of each year. *Pres.* H. M. Grant, Abington: *Sec.* G. F. Kersee, Richmond.

*Washington, City Dental Society* Meets annually. *Pres.* R. F. Hunt, *Sec.* Dr. Evans.

*Wabash Valley Dental Association* Meets annually. Next meeting at Wayne, third Tuesday in March, 1872. *Pres.* A. B. Cunningham, Attica: *Sec.* S. B. Brown, Fort Wayne.

* *Wisconsin State Dental Association* Meets semi-annually. Next meeting at Watertown. second Tuesday of January, 1872. *Pres.* Edgar Palmer, Le Crook *Sec.* C. C. Chittenden, Madison.

**Place of meeting fixed by appointment,*

### District Dental Societies of the State of New York.

*First District Dental Society* Meets Monday in New York. Annual meeting, second Wednesday in June : *Pres.* E. A. Bogue, New York : *Sec.* J. S. Latimer,. N. Y.

*Second District Dental Society.* Meets quarterly in Brooklyn. Annual meeting second Tuesday in April. *Pres.* W. S. Elliott : *Rec. Sec.* M. E. Elmandorf. Brooklyn : *Cor.* F. W. Dalbare.

*Third District Dental Society* Meets at Albany semi-annually and annually. *Pres.* D. D. French, Troy : *Sec.* S. J. Andress, Albany.

**Fourth District Dental Society* Meets annually in June at Saratoga. *Pres.* C, A. Elmore, Saratoga Springs. *Sec.* E. S. Pearsall, Fort Edwards.

**Fifth District Dental Society* Meets annually at Saratoga. *Pres.* W. W. Perkins *Sec.* F. D. Neelans.

**Sixth District Dental Society* Annual meeting at Binghampton, second Tuesday in June. *Pres.* R. Walker : *Rec. Sec.* P. L. Foote

**Seventh District Dental Society* Annual meeting is held the last Tuesday in June, at Rochester. *Pres.* G. W. Tripp, Auburn ; *Sec.* J. E. Line, Rochester.

*Eighth District Dental Society* Meets annually on the second Tuesday in May, at Buffalo : *Bres.* J. C. Gifford, *Sec.* S. A. Freeman, Buffalo.

T*he Seventh and Eighth District Societies* Hold a union meeting on the first Tuesday of October, alternately at Buffalo and Rochester.

*Place and time of meeting fixed by appointment.

---

The Ohio State Board of Dental Examiners will meet in Columbus on the first Tuesday of Dec. 1874, at 10 o'clock. *Pres.* J. Taft ; *Sec.* W. P. Horton.

---

### List of Exchanges.

ST. LOUIS MEDICAL AND SURGICAL JOURNAL.
BOSTON MEDICAL AND SURGICAL JOURNAL.
THE PACIFIC MEDICAL AND SURGICAL JOURNAL, SAN FRANCISCO.
BUFFALO MEDICAL AND SURGICAL JOURNAL.
PHIDADELPHIA MEDICAL AND SURGICAL JOURNAL.
CINCINNATI LANCET AND OBSERVER.
DENTAL COSMOS, PHILADELPHIA.
BRAITHWAITE'S RETROSPECT, N. Y.
THE DENTAL TIMES, PHILADELPHIA.
LADIES' REPOSITORY, CINCINNATI.
HALL'S JOURNAL OF HEALTH, N. Y.
CHICAGO MEDICAL EXAMINER.
THE DETROIT REVIEW OF MEDICINE AND PHARMACY.
MEDICAL RECORD, N. Y.
NASHVILLE JOURNAL OF MEDICINE AND SURGERY.
AMERICAN ECLECTIC MEDICAL REVIEW. N. Y..
AMERICAN JOURNAL OF DENTAL SCIENCE, BALTIMORE.
THE UNITED STATES MEDICAL AND SURGICAL JOURNAL. CHICAGO
NEW ORLEANS JOURNAL OF MEDICINE.
WESTERN JOURNAL OF MEDICINE, INDIANAPOLIS, IND.
AMERICAN AGRICULTURIST, N. Y.
BOSTON JOURNAL OF CHEMISTRY.
JOURNAL OF APPLIED CHEMISTRY, N. Y.
DRUGGISTS' CIRCULAR AND CHEMICAL GAZETTE, N. Y.
CANADA JOURNAL OF DENTAL SCIENCE, MONTREAL.
INDIANA JOURNAL OF MEDICINE.
JOURNAL OF THE GYNÆCOLOGICAL SOCIETY, BOSTON.
MEDICAL AND SURGICAL REPORTER, SALEM, OREGON.
MISSOURI DENTAL JOURNAL, ST. LOUIS
ECLECTIC MEDICAL JOURNAL, CINCINNATI.

THE POCKET

### DENTAL REGISTER.

Every dentist with anything like a full practice, and who cares to conduct business in a systematic manner, must have a book in which to record his dental operations.

The large "Registers" that have been used for that purpose, are excellent in their place, and may still be used in connection with such a book as this, if desired; but they are open to several important objections.

Many dentists using them have doubtless experienced the inconvenience of going to the desk after each patient's sitting, to make a record, and have preferred trusting to memory till after office hours. By that time, the exact tooth operated upon has often been forgotten; and sometimes a certain operation has slipped from the mind altogether.

The fillings put in to-day are marked on the same diagram with those inserted a month or a year ago; consequently, after a little while, it is impossible to distinguish between old and recent work.

The space reserved for each account being so large, one is unwilling to spoil a blank to record trifling services, such as extracting or treating a tooth for a patient whom he may never see again; although it often happens afterward that he would be glad to have a knowledge of the service.

In recording operations on the temporary teeth, it is very unsatisfactory to cross out the twelve back teeth of the permanent set, and imagine the bicuspids to be temporary molars. In such cases, much confusion generally follows in the course of a few years.

This Pocket Register is presented with the belief that it is free from these objectionable features.

Each operation may be recorded with pen or pencil, right at the chair, in almost the same time that an appointment is made for another sitting.

Every operation bears its own date, and cannot be taken for a previous or later one.

The space for accounts is so economically used, that one will not hesitate to record the most trivial services. It consists of 224 pages, arranged as follows:

120 pages for small account , ten to the page, with blanks for date, name, residence, operation, debit, and credit. Each account is also furnished with a diagram of the teeth for marking operations performed.
24 pages for medium accounts, two to the page, with blanks and diagram, as above.
16 pages for large accounts, one to the page, with blanks and diagram.
16 pages with blanks and diagrams expressly arranged for operations on the temporary teeth, ten accounts to the page.
32 pages for appointments for 64 weeks. These are without date, and may, therefore be used for any time,
16 pages for index and memoranda.

It will be seen that the "Pocket Register" combines Account-book, Record of Operations on the Permanent and Temporary Teeth, Appointment-book, and Memoranda; and that the whole is offered for about the price of an ordinary Engagement book.

It is printed on fine writing paper, bound handsomely in leather, with tuck, or in English cloth, and is of a suitable size for carrying in the breast pocket.

— PRICE. —

*In Leather, with Tuck.* ........ $1 50
*In English Cloth,* ........ 1 25

Sent post-paid, on receipt of price. For sale, wholesale and retail, by

SPENCER & MOORE, Ohio Dental Depot,
Cincinnati, O.

AMERICAN DENTAL ASSOCIATION—Meets on the first Tuesday in Aug., 1874, at Detroit. *Pres.* T. L. Buckingham, Philad.; *Cor. Sec.* J. Taft, Cincinnati, O.; *Rec. Sec.* M. S. Dean, Chicago.

*American Dental Convention* Meets at Saratoga Springs, second Tuesday in Aug., 1874. *Pres.* John Allen, New York City; *Cor. Sec.* A. Tees, Philadelphia, Pa., *Rec. Sec.* C. L. Hurlbut, Springfield, Mass.

*List of Societies Represented in the American Dental Association.*

*American Academy of Dental Science* Meets at Boston, Mass. *Pres.* Joshua Tucker, M. D., of Boston; *Sec.* W. L. Tucker, of Boston; *Cor. Sec.*, E. N. Harris.

*Brooklyn Dental Society* Meets semi-monthly. *Pres.* W. T. Shannon,; *Rec. Sec.* C. P. Crandell; *Cor. Sec.* A. N. Chapman.

*Buffalo Dental Society*Meets second Monday of each month at Buffalo. *Pres.* S. A. FREEMAN; *Rec. Sec.* G. C. DABOLL.

*Boston Dental Institute*—Meets first Tuesday of each month. *Pres.* D. G. WILLIAMS; *Cor. Sec.* D. S. DICKERMAN.

*California State Dental Association* Meets annually in June. *Pres.* C. C. Knowles, San Francisco; *Rec. Sec.* H. J. Plomteaux, Woodland.

*Cincinnati Dental Society*—Meets Semi-monthly at Cincinnati. *Pres.* H. R. SMITH; *Rec. Sec.* D. C. HAWXHURST.

*Chicago Dental Society* Meets monthly at Chicago. *Pres.* M. S. Dean; *Rec. Sec.* Edgar D. Swain.

*Connecticut Valley Dental Association.* Meets semi-annually, second Tuesday of June and October. *Pres.* H. M. Miller, Westfield, Mass.; *Rec. Sec.* L. C. Taylor, of Holyoke, Mass.

*Connecticut State Dental Association.* *Pres.* R. C. Dunham, New Britain; *Rec. Sec.* D. E. Lane, Hartford.

*Charleston Dental Association*—Meets second Tuesday of every month; *Pres.* T. F. Chupein; *Sec. and Treas.* B. A. Muckenfuss.

*Delaware Dental Association*—Meets semi-annually. *Pres.* E. W. Haines, Newark; *Sec.* C. R. Jeffries, Wilmington.

*Eastern Ohio Dental Association*—Meets on Tuesday, August 26th, 1873, at Alliance; *Pres.* A. J. Douds; *Sec.* J. M. Porter.

*East Tennessee Dental Association*—Meets annually at Knoxville, Tenn. *Pres.* Wm. H. Cooke, of Cleveland; *Rec. Sec.* A. P. White, Knoxville.

*Forest City Society of Dental Surgeons*—*Pres.* B. Strickland, Cleveland, *Rec. Sec.* H. L. Ambler, Cleveland; *Cor. Sec.* Chas. Buffett, Cleveland.

*Georgia State Dental Society* Next meeting at Atlanta, July 1873. *Pres.* F. Y. Clark; *Cor. Sec.* J. P. H. Brown, Augusta.

*Harris Dental Association* Meets quarterly on the first Thursday in February, May, August, November. *Pres.* J. A. Martin, Strasburg; *Sec.* Wm. N. Amer, Lancaster,

*Hudson River Association of Dental Surgeons* Meets on the second Wednesdays of May, September and January. *Pres.* L. S. Straw, Newburg, N. Y; *Rec. Sec.* T. W. DuBois, Poughkeepsie, N. Y.

*Hudson Valley Dental Association*—Meets monthly at Troy, N. Y. *Pres.* L. C. Wheeler, *Rec. Sec.* S. G. Andres; *Cor. Sec.* S. D. French.

*Illinois State Dental Soeiety* Meets annually. *Pres.* C. Stoddard Smith, Springfield; *Sec.* C. R. E. Koch, Chicago. Meets second Tuesday in May, 1874, at Jacksonville.

*Indiana State Dental Society*—Meets next year at Indianapolis, on the first Tuesday of June. *Pres.* M. Wells, Indianapolis; *Cor. and Rec. Sec.* P. McDonald, Goshen.

*Iowa State Dental Society*—Meets annually. Next meeting at Davenport, on the second Tuesday of May. *Pres.* L. C. Ingersoll, Keokuk. *Rec. Sec.* E. P. White, of Davenport; *Cor. Sec.* A. J. Redrich, Sioux City.

*Kansas State Dental Society* Meets semi-annually. Next meeting at Topeka, Sept. 12, 1873. *Pres.* J. B. Wheeler; *Sec.* E. C. Fuller.

*Kansas and Missouri Valley Dental Association*—Meets first and third Tuesdays of erch month. *Pres.* J. R. Stark; *Sec.* H. C. Massie, Kansas City, Mo.

*Kentucky State Dental Association* Meets annually and semi-monthly, in the City of Louisville. Annnal meeting, first Tuesday in June, at 2 o'clock, P. M. Semi-monthly meetings, second and fourth Tuesday nights. G. W. Priest, *Pres.*; B. O. Doyle, *Sec.*, both at Louisville.

*Lake Erie Dental Association* Meets Annually. *Pres.* C. B. Ansart, Oil City, Pa.; *Sec.* C. D. Elliot, Franklin, Pa. Next meeting first Tuesday of May, 1874.

*Mad River Dental Association* Meets at Dayton on the first Tuesdays in April and October. *Pres.* G. W. Keely, Oxford; *Sec.* J. A Stipp, Sidney.

*Maine Dental Society*—Meets in Aug., at Thomaston. *Pres.* E. J. Roberts, Augusta; *Sec.* C. E. Hussey, Biddeford.

*Maryland Association of Dentists* Meets the last Thursday of every month. *Pres.* J. B. Bean; *Sec.* S. M. Field.

*Massachusetts Dental Society*—Meets monthly. *Pres.* J. H. Batchelder, of Salem; *Sec.* C. Wilson; *Cor. Sec.* L. D. Shepard.

*Michigan State Dental Association*—Meets annually second Tuesday of Oct., at Ypsilanti. *Pres.* D. C. Hawxhurst, Ann Arbor; *Rec. & Cor. Sec.* T. R. Perry, Grand Rapids

*Michigan Central Dental Association.* Meets annually at Jackson, Secon Wednesday of January, 1873. *Pres.* C. C. Jenkins, Anu Arbor, *Sec.* W. H. Darrance, Jackson.

*Mississippi Valley Dental Association* Meets annually at St. Louis, on first Wednesday in March, 1874. *Pres.* W. H. Eames, St. Louis, Mo.; *Rec. Sec.* F. Hunter Cin.

*Minnesota Dental Associrtion* Meets annually. Next meeting at Faribault, last Wednesday in June, 1874. *Pres.* J. A. Bowman, Minneapolis; *Sec.* C. D. Montreville, St. Paul.

*Missouri Dental Association* Meets on the first Tuesday of June, in St. Joseph. *Pres.* A. S. Billings, Omaha, Neb.; *Sec.* G. F. Sanborn, Tabor, Iowa.

*Missouri Valley Dental Association** Meets on the second Tuesday in July and January. *Pres.* A. S. Billings, Omaha, Neb.; *Sec. and Treas.* J. F. Sanborn, Tabor, Iowa.

*Merrimac Valley Dental Association* Meets semi-annually first Tuesday of Oct. and May.* *Pres.* L. H. Kidder, Mass; *Rec. Sec.* A. M. Dudley, of Lowell; *Cor. Sec.* D. T. Porter, Lawrence, Mass.

*Memphis Dental Society*—Meets on the first Tuesday of each month. *Pres.* W. M. Arrington; *Sec.* V. F. Elliott; *Treas.* C. L. Harris.

*Nashville Dental Association*—Meets on the second Tuesday of each month. *Pres* J. C. Ross; *Sec.* R. R. Freeman; *Cor. Sec.* S. J. Cobb.

*New Jersey State Dental Society*—Meets annually. Next meeting at Mount Holly, on the second Tuesday in July, 1874. *Pres.* J. W. Cosad, Jersey City; *Sec.* J. W. Scarborough, Lambertville.

*New York State Dental Society*--Meets at Albany on the last Wednesday of June next. *Pres.* J. G. Ambler, New Yook; *Sec.* Chas. Barnes, of Syracuse, *Cor. Sec.* S. A. Freeman.

*New Haven Dental Society*—Meets on the first Wednesday of each month at New Haven. *Pres.* J. T. Metcalf, *Rec and Cor. Sec.* C. L. Smith, of New Haven.

*New Orleans Dental Society* Meets monthly. *Pres.* ——————; *Rec. Sec.* A. F McLane.

*Northern Ohio Dental Association.* Meets annually, second Tuesday of June, 1873, at Put-in Bay. *Pres.* J. A. Robinson, Cleveland; *Rec. Sec.* A. F. Price, Fremont.

*Northern Iowa Dental Association*—Meets annually, on the second Tuesday in June, 1873. Next meeting at Waterloo; *Pres.* J. N. Abbott, Lancaster, *Rec. Sec.* A. V. Eaton, Anamosa; *Cor. Sec.* A. R. Mason, Waterloo.

*Ohio College Dental Association*--Meets annually Feb. 25. at Cincinnati. *Pres.* Jas. Leslie, Cin'ti.; *Rec. Sec.* H. A. Smith, of Cincinnati.

*Ohio State Dental Association*--Meets annually at Columbus. Next meeting, first Wednesday of December. *Pres.* H. A. Smith, Cincinnati; *Rec. Sec.* J. M. Porter, Massilon; *Cor. Sec.* C. R. Butler, Cleveland, O.

*Odontographic Society of Pennsylvania*—Meets monthly in Philadelphia. Next meeting, Wednesday, Sept. 1st. *Pres.* C. T. Stellwagen; *Rec. Sec.* A. Boice; *Cor. Sec.* W. F. Litch.

*Odontological Society of New York City* Meets the second Tuesday of each monh. *Pres.* C. E. Francis; *Rec. Sec.* W. E. Hong.

*Old Colony Dental Association* Meets quarterly.* *Pres.* C. G. Davis; *Rec. Sec.* L. W. Puffer, North Bridgewater, Mass; *Cor. Sec.* N. C. Fowler, Yarmouth, Mass.

*Ohio Valley Dental Association* Meets annually, in N. E. Ky; this year in Paris, Ky. Tuesday, Sept. 9th.

*Oregon State Dental Society* Meets annually. *Pres.* J. H. Hatch, Portland; *Rec. Sec.* J. R. Cardwell, Portland.

*Pennsylvania State Dental Society* Meets next at Wilkesbarre, second Tuesday of July, 1874. *Pres.* G. W. Klump, Williamsport; *Rec. Sec.* R. H. Moffit, Harrisburg *Cor. Sec.* S. Welchens, Lancaster.

*Poughkeepsie Dental Society* Meets monthly. *Pres.* J. H. Mann; *Sec.* H. F. Clark

*San Francisco Dental Association* Meets monthly. *Pres.* C. C. Knowles; *Cor. Sec.* H. E. Knox; *Rec. Sec.* Wm. Dutch.

*St. Louis Dental Society* Meets monthly. *Pres.* W. H. Morrison; *Sec.* A. H. Fuller.

*Susquehanna Dental Association* Meets annually.* *Pres.* J. L. Fordham: *Rec. Sec.* G. W. Klump, Williamsport. Meets Nov. 12, 1873.

*Society of Dental Surgeons of the City of New York* Meets semi-monthly in New York. *Pres.* B. F. Franklin: *Rec. Sec.* J. S. Latimer; *Cor. Sec.* E. H. Burras.

**Southern Dental Association* Meets annually. Next meeting the last Tuesday of July, 1873, at Baltimore, Md. *Pres.* H. M. Grant Virginia.: *Rec. Sec.* J. F. Thompson Va

*South Carolina State Dental Association* Meets semi-annually in May and Nov. *Pres.* W. C. Wardlaw, Abbeville: *Sec.* O. J. Bond, Marion.

*Tennessee Dental Association* Meets semi-annually. Next meeting in Nashville, on the 4th Wednesday in June. 1874. *Pres.* R. Russell, Nashville: *Sec.* A. Hartman, Murfreesboro; *Cor. Sec.* L. G. Noel, Nashville.

*Virginia State Dental Association* Meets in Richmond, first Thursday in Nov. of each year. *Pres.* H. M. Grant, Abington: *Sec.* G. F. Kersee, Richmond.

*Washington, City Dental Society* Meets annually. *Pres.* R. F. Hunt, *Sec.* Dr. Evans.

*Wabash Valley Dental Association* Meets annually. Next meeting at Wayne, third Tuesday in March, 1872. *Pres.* A. B. Cunningham, Attica: *Sec.* S. B. Brown, Fort Wayne.

* *Wisconsin State Dental Association* Meets semi-annually. Next meeting at Watertown. second Tuesday of January, 1872. *Pres.* Edgar Palmer, Le Crook *Sec.* C. C. Chittenden, Madison.

**Place of meeting fixed by appointment,*

### District Dental Societies of the State of New York.

*First District Dental Society* Meets Monday in New York. Annual meeting, second Wednesday in June : *Pres.* E. A. Bogue, New York : *Sec.* J. S. Latimer,. N. Y.

*Second District Dental Society.* Meets quarterly in Brooklyn. Annual meeting second Tuesday in April. *Pres.* W. S. Elliott : *Rec. Sec.* M. E. Elmandorf. Brooklyn : *Cor.* F. W. Dalbare.

*Third District Dental Society* Meets at Albany semi-annually and annually. *Pres.* D. D. French, Troy : *Sec.* S. J. Andress, Albany.

**Fourth District Dental Society* Meets annually in June at Saratoga. *Pres.* C, A. Elmore, Saratoga Springs. *Sec.* E. S. Pearsall, Fort Edwards.

**Fifth District Dental Society* Meets annually at Saratoga. *Pres.* W. W. Perkins *Sec.* F. D. Neelans.

**Sixth District Dental Society* Annual meeting at Binghampton, second Tuesday in June. *Pres.* R. Walker : *Rec. Sec.* P. L. Foote

**Seventh District Dental Society* Annual meeting is held the last Tuesday in June, at Rochester. *Pres.* G. W. Tripp, Auburn ; *Sec.* J. E. Line, Rochester.

*Eighth District Dental S ciety* Meets annually on the second Tuesday in May, at Buffalo : *Bres.* J. C. Gifford, *Sec.* S. A. Freeman, Buffalo.

T*he Seventh and Eighth District Societies* Hold a union meeting on the first Tuesday of October, alternately at Buffalo and Rochester.

*Place and time of meeting fixed by appointment.

---

The Ohio State Board of Dental Examiners will meet in Columbus on the first Tuesday of Dec. 1874, at 10 o'clock. *Pres.* J. Taft ; *Sec.* W. P. Horton.

---

### List of Exchanges.

ST. LOUIS MEDICAL AND SURGICAL JOURNAL.
BOSTON MEDICAL AND SURGICAL JOURNAL.
THE PACIFIC MEDICAL AND SURGICAL JOURNAL, SAN FRANCISCO.
BUFFALO MEDICAL AND SURGICAL JOURNAL.
PHIDADELPHIA MEDICAL AND SURGICAL JOURNAL.
CINCINNATI LANCET AND OBSERVER.
DENTAL COSMOS, PHILADELPHIA,
BRAITHWAITE'S RETROSPECT, N. Y.
THE DENTAL TIMES, PHILADELPHIA.
LADIES' REPOSITORY, CINCINNATI.
HALL'S JOURNAL OF HEALTH, N. Y.
CHICAGO MEDICAL EXAMINER,
THE DETROIT REVIEW OF MEDICINE AND PHARMACY.
MEDICAL RECORD, N. Y.
NASHVILLE JOURNAL OF MEDICINE AND SURGERY.
AMERICAN ECLECTIC MEDICAL REVIEW. N. Y.,
AMERICAN JOURNAL OF DENTAL SCIENCE, BALTIMORE.
THE UNITED STATES MEDICAL AND SURGICAL JOURNAL. CHICAGO
NEW ORLEANS JOURNAL OF MEDICINE,
WESTERN JOURNAL OF MEDICINE; INDIANAPOLIS, IND.
AMERICAN AGRICULTURIST, N. Y.
BOSTON JOURNAL OF CHEMISTRY.
JOURNAL OF APPLIED CHEMISTRY, N. Y.
DRUGGISTS' CIRCULAR AND CHEMICAL GAZETTE, N. Y.
CANADA JOURNAL OF DENTAL SCIENCE, MONTREAL.
INDIANA JOURNAL OF MEDICINE,
JOURNAL OF THE GYNÆCOLOGICAL SOCIETY, BOSTON.
MEDICAL AND SURGICAL REPORTER, SALEM, OREGON.
MISSOURI DENTAL JOURNAL, ST. LOUIS
ECLECTIC MEDICAL JOURNAL, CINCINNATI.

### THE POCKET

### DENTAL REGISTER.

Every dentist with anything like a full practice, and who cares to conduct business in a systematic manner, must have a book in which to record his dental operations.

The large "Registers" that have been used for that purpose, are excellent in their place, and may still be used in connection with such a book as this, if desired; but they are open to several important objections.

Many dentists using them have doubtless experienced the inconvenience of going to the desk after each patient's sitting, to make a record, and have preferred trusting to memory till after office hours. By that time, the exact tooth operated upon has often been forgotten; and sometimes a certain operation has slipped from the mind altogether.

The fillings put in to-day are marked on the same diagram with those inserted a month or a year ago; consequently, after a little while, it is impossible to distinguish between old and recent work.

The space reserved for each account being so large, one is unwilling to spoil a blank to record trifling services, such as extracting or treating a tooth for a patient whom he may never see again; although it often happens afterward that he would be glad to have a knowledge of the service.

In recording operations on the temporary teeth, it is very unsatisfactory to cross out the twelve back teeth of the permanent set, and imagine the bicuspids to be temporary molars. In such cases, much confusion generally follows in the course of a few years.

This Pocket Register is presented with the belief that it is free from these objectionable features.

Each operation may be recorded with pen or pencil, right at the chair, in almost the same time that an appointment is made for another sitting.

Every operation bears its own date, and cannot be taken for a previous or later one.

The space for accounts is so economically used, that one will not hesitate to record the most trivial services. It consists of 224 pages, arranged as follows:

120 pages for small account , ten to the page, with blanks for date, name, residence, operation, debit, and credit. Each account is also furnished with a diagram of the teeth for marking operations performed.
24 pages for medium accounts, two to the page, with blanks and diagram, as above.
16 pages for large accounts, one to the page, with blanks and diagram.
16 pages with blanks and diagrams expressly arranged for operations on the temporary teeth, ten accounts to the page.
32 pages for appointments for 64 weeks. These are without date, and may, therefore, be used for any time,
16 pages for index and memoranda.

It will be seen that the "Pocket Register" combines Account-book, Record of Operations on the Permanent and Temporary Teeth, Appointment-book, and Memoranda; and that the whole is offered for about the price of an ordinary Engagement book.

It is printed on fine writing paper, bound handsomely in leather, with tuck, or in English cloth, and is of a suitable size for carrying in the breast pocket.

— PRICE. —

*In Leather, with Tuck.* ........................................ $1 50
*In English Cloth,* ........................................ 1 25

Sent post-paid, on receipt of price. For sale, wholesale and retail, by

SPENCER & MOORE, Ohio Dental Depot,
Cincinnati, O,

AMERICAN DENTAL ASSOCIATION—Meets on the first Tuesday in Aug., 1874, at Detroit. *Pres.* T. L. Buckingham, Philad.; *Cor. Sec.* J. Taft, Cincinnati, O.; *Rec. Sec.* M. S. Dean, Chicago.

*American Dental Convention* Meets at Saratoga Springs, second Tuesday in Aug., 1874. *Pres.* John Allen, New York City; *Cor. Sec.* A. Tees, Philadelphia, Pa., *Rec. Sec.* C. L. Hurlbut, Springfield, Mass.

*List of Societies Represented in the American Dental Association.*

*American Academy of Dental Science* Meets at Boston, Mass. *Pres.* Joshua Tucker, M. D., of Boston; *Sec.* W. L. Tucker, of Boston; *Cor. Sec.*, E. N. Harris.

*Brooklyn Dental Society* Meets semi-monthly. *Pres.* W. T. Shannon,; *Rec. Sec.* C. P. Crandell; *Cor. Sec.* A. N. Chapman.

*Buffalo Dental Society*Meets second Monday of each month at Buffalo. *Pres.* S. A. FREEMAN; *Rec. Sec.* G. C. DABOLL.

*Boston Dental Institute*—Meets first Tuesday of each month. *Pres.* D. G. WILLIAMS; *Cor. Sec.* D. S. DICKERMAN.

*California State Dental Association* Meets annually in June. *Pres.* C. C. Knowles, San Francisco; *Rec. Sec.* H. J. Plomteaux, Woodland.

*Cincinnati Dental Society*—Meets Semi-monthly at Cincinnati. *Pres.* H. R. SMITH; *Rec. Sec.* D. C. HAWXHURST.

*Chicago Dental Society* Meets monthly at Chicago. *Pres.* M. S. Dean; *Rec. Sec.* Edgar D. Swain.

*Connecticut Valley Dental Association.* Meets semi-annually, second Tuesday of June and October. *Pres.* H. M. Miller, Westfield, Mass.; *Rec. Sec.* L. C. Taylor, of Holyoke, Mass.

*Connecticut State Dental Association.* *Pres.* R. C. Dunham, New Britain; *Rec. Sec.* D. E. Lane, Hartford.

*Charleston Dental Association*—Meets second Tuesday of every month; *Pres.* T. F. Chupein; *Sec. and Treas.* B. A. Muckenfuss.

*Delaware Dental Association*—Meets semi-annually. *Pres.* E. W. Haines, Newark; *Sec.* C. R. Jeffries, Wilmington.

*Eastern Indiana Dental Association.*—Meets semi-annually, first Tuesday in May and last Tuesday in October; *Pres.* W. C. Stanley, Dublin, Ind.; *Sec.* J. W. White, Knightstown, Ind. (Place of meeting fixed by appointment.)

*Eastern Ohio Dental Association*—Meets on Tuesday, August 26th, 1873, at Alliance; *Pres.* A. J. Douds; *Sec.* J. M. Porter.

*East Tennessee Dental Association*—Meets annually at Knoxville, Tenn. *Pres.* Wm. W. F. Fowler; *Rec. Sec.* S. M. Prothro.

*Forest City Society of Dental Surgeons*—*Pres.* B. Strickland, Cleveland, *Rec. Sec.* H. L. Ambler, Cleveland; *Cor. Sec.* Chas. Buffett, Cleveland.

*Georgia State Dental Society* Next meeting at Atlanta, July 1873. *Pres.* F. Y. Clark; *Cor. Sec.* J. P. H. Brown, Augusta.

*Harris Dental Association* Meets quarterly on the first Thursday in February, May, August, November. *Pres.* J. A. Martin, Strasburg; *Sec.* Wm. N. Amer, Lancaster,

*Hudson River Association of Dental Surgeons* Meets on the second Wednesdays of May, September and January. *Pres.* L. S. Straw, Newburg, N. Y; *Rec. Sec.* T. W. DuBois, Poughkeepsie, N. Y.

*Hudson Valley Dental Association* Meets monthly at Troy, N. Y. *Pres.* L. C. Wheeler, *Rec. Sec.* S. G. Andres; *Cor. Sec.* S. D. French.

*Illinois State Dental Society* Meets annually. *Pres.* G. S. Miles, Jerseyville, *Sec.* C. R. E. Koch, Chicago. Meets second Tuesday in May, 1874, at Ottowa.

*Indiana State Dental Society*—Meets next year at Indianapolis, on the first Tuesday of June. *Pres.* M. Wells, Indianapolis; *Cor. and Rec. Sec.* P. McDonald, Goshen.

*Iowa State Dental Society*—Meets annually. Next meeting at Davenport, on the second Tuesday of May. *Pres.* L. C. Ingersoll, Keokuk. *Rec. Sec.* E. P. White, of Davenport; *Cor. Sec.* A. J. Redrich, Sioux City.

*Kansas State Dental Society* Meets semi-annually. Next meeting at Topeka, Sept. 12, 1873. *Pres.* Dr. Griswold; *Sec.* A. H. Thompson.

*Kansas and Missouri Valley Dental Association*—Meets first and third Tuesdays of erch month. *Pres.* J. R. Stark; *Sec.* H. C. Massie, Kansas City, Mo.

*Kentucky State Dental Association* Meets annually and semi-monthly, in the City of Louisville. Annnal meeting, first Tuesday in June, at 2 o'clock, P. M. Semi-monthly meetings, second and fourth Tuesday nights. G. W. Priest, *Pres.*; B. O. Doyle. *Sec.*, both at Louisville.

*Lake Erie Dental Association* Meets Annually. *Pres.* C. B. Ansart, Oil City, Pa. *Sec.* C. D. Elliot, Franklin, Pa. Next meeting first Tuesday of May, 1874.

*Mad River Dental Association* Meets at Dayton on the first Tuesdays in April and October. *Pres.* S. B. Tizzard, Dayton; *Sec.* A. J. Grosvenor, Troy.

*Maine Dental Society*—Meets in Aug., at Thomaston. *Pres.* E. J. Roberts, Augusta; *Sec.* C. E. Hussey, Biddeford.

*Maryland Association of Dentists* Meets the last Thursday of every month. *Pres.* J. B. Bean; *Sec.* S. M. Field.

*Massachusetts Dental Society*—Meets monthly. *Pres.* J. H. Batchelder, of Salem; *Sec.* C. Wilson; *Cor. Sec.* L. D. Shepard.

*Michigan State Dental Association*—Meets annually second Tuesday of Oct., at Ypsilanti. *Pres.* D. C. Hawxhurst, Ann Arbor; *Rec. & Cor. Sec.* T. R. Perry, Grand Rapids.

*Michigan Central Dental Association.* Meets annually at Jackson, Secon Wednesday of January, 1873. *Pres.* C. C. Jenkins, Anu Arbor, *Sec.* W. H. Darrance, Jackson.

*Mississippi Valley Dental Association* Meets annually at Cincinnati, on first Wednesday in March, 1874. *Pres.* S. B. Brown, Ft. Wayne Ind.; *Rec. Sec.* F. Hunter Cin.

*Minnesota Dental Associrtion* Meets annually. Next meeting at Faribault, last Wed nesday in June, 1874. *Pres.* J. A. Bowman, Minneapolis; *Sec.* C. D. Montreville, St. Paul.

*Missouri Dental Association* Meets on the first Tuesday of June, in St. Louis. *Pres.* C. W. Rivers, St. Louis; *Sec.* W. H. Evans, St. Louis.

*Missouri Valley Dental Association** Meets on the second Tuesday in July and January. *Pres.* A. S. Billings, Omaha, Neb.; *Sec. and Treas.* J. F. Sanborn, Tabor, Iowa.

*Merrimac Valley Dental Association* Meets semi-annually first Tuesday of Oct. and May.* *Pres.* L. H. Kidder, Mass; *Rec. Sec.* A. M. Dudley, of Lowell; *Cor. Sec.* D. T. Porter, Lawrence, Mass.

*Memphis Dental Society*--Meets on the first Tuesday of each month. *Pres.* W. M. Arrington; *Sec.* V. F. Elliott; *Treas.* C. L. Harris.

*Nashville Dental Association*--Meets on the second Tuesday of each month. *Pres* J. C. Ross; *Sec.* R. R. Freeman; *Cor. Sec.* S. J. Cobb.

*New Jersey State Dental Society*--Meets annually. Next meeting at Mount Holly, on the second Tuesday in July, 1874. *Pres.* J. W. Cosad, Jersey City; *Sec.* J. W. Scarborough, Lambertville.

*New York State Dental Society*--Meets at Albany on the last Wednesday of June next. *Pres.* J. G. Ambler, New Yook; *Sec.* Chas. Barnes, of Syracuse, *Cor. Sec.* S. A. Freeman.

*New Haven Dental Society*--Meets on the first Wednesday of each month at New Haven. *Pres.* J. T. Metcalf, *Rec and Cor. Sec.* C. L. Smith, of New Haven.

*New Orleans Dental Society* Meets monthly. *Pres.*————————; *Rec. Sec.* A. F McLane.

*Northern Ohio Dental Association.*Meets annually, second Tuesday of June, 1873, at Put-in Bay. *Pres.* J. A. Robinson, Cleveland; *Rec. Sec.* A. F. Price, Fremont.

*Northern Iowa Dental Association*—Meets annually, on the second Tuesday in June, 1873. Next meeting at Waterloo; *Pres.* J. N. Abbott, Lancaster, *Rec. Sec.* A. V. Eaton, Anamosa; *Cor. Sec.* A. R. Mason, Waterloo.

*Ohio College Dental Association*--Meets annually Feb. 25. at Cincinnati. *Pres.* Jas. Leslie, Cin'ti.; *Rec. Sec.* H. A. Smith, of Cincinnati.

*Ohio State Dental Association*--Meets annually at Columbus. Next meeting, first Wednesday of December. *Pres.* H. A. Smith, Cincinnati; *Rec. Sec.* J. M. Porter, Massilon; *Cor. Sec.* C. R. Butler, Cleveland, O.

*Odontographic Society of Pennsylvania*--Meets monthly in Philadelphia. Next meeting, Wednesday, Sept. 1st. *Pres.* C. T. Stellwagen; *Rec. Sec.* E. L. Hewitt.

*Odontological Society of New York City* Meets the second Tuesday of each monh. *Pres.* C. E. Francis; *Rec. Sec.* W. E. Hong.

*Old Colony Dental Association* Meets quarterly.* *Pres.* C. G. Davis; *Rec. Sec.* L. W. Puffer, North Bridgewater, Mass; *Cor. Sec.* N. C. Fowler, Yarmouth, Mass.

*Ohio Valley Dental Association* Meets annually, in N. E. Ky; this year in Paris, Ky. Tuesday, Sept. 9th.

*Oregon State Dental Society* Meets annually. *Pres.* J. H. Hatch, Portland; *Rec. Sec.* J. R. Cardwell, Portland.

*Pennsylvania State Dental Society* Meets next at Wilkesbarre, second Tuesday of July, 1874. *Pres.* G. W. Klump, Williamsport; *Rec. Sec.* R. H. Moffit, Harrisburg *Cor. Sec.* S. Welchens, Lancaster.

*Poughkeepsie Dental Society* Meets monthly. *Pres.* J. H. Mann; *Sec.* H. F. Clark

*Pennsylvania Association of Dental Surgery* Meets monthly in Philadelphia. *Pres.* Spencer Roberts; *Sec.* E. R. Pettit.

*San Francisco Dental Association* Meets monthly. *Pres.* C. C. Knowles; *Cor. Sec.* H. E. Knox; *Rec. Sec.* Wm. Dutch.

*St. Louis Dental Society* Meets monthly. *Pres.* W. H. Morrison; *Sec.* A. H. Fuller.

*Susquehanna Dental Association* Meets annually.* *Pres.* J. L. Fordham: *Rec. Sec.* G. W. Klump, Williamsport. Meets Nov. 12, 1873.

*Society of Dental Surgeons of the City of New York* Meets semi-monthly in New York. *Pres.* B. F. Franklin: *Rec. Sec.* J. S. Latimer; *Cor. Sec.* E. H. Burras.

**Southern Dental Association* Meets annually. Next meeting the last Tuesday of July, 1873, at Baltimore, Md. *Pres.* H. M. Grant Virginia.: *Rec. Sec.* J. F. Thompson Va

*South Carolina State Dental Association* Meets semi-annually in May and Nov. *Pres.* W. C. Wardlaw, Abbeville: *Sec.* O. J. Bond, Marion.

*Tennessee Dental Association* Meets semi-annually. Next meeting in Nashville, on the 4th Wednesday in June. 1874. *Pres.* R. Russell, Nashville: *Sec.* A. Hartman, Murfreesboro; *Cor. Sec.* L. G. Noel, Nashville.

*Virginia State Dental Association* Meets in Richmond, first Thursday in Nov. of each year. *Pres.* H. M. Grant, Abington: *Sec.* G. F. Kersee, Richmond.

*Washington, City Dental Society* Meets annually. *Pres.* R. F. Hunt, *Sec.* Dr. Evans.

*Wabash Valley Dental Association* Meets annually. Next meeting at Wayne, third Tuesday in March, 1872. *Pres.* A. B. Cunningham, Attica: *Sec.* S. B. Brown, Fort Wayne.

* *Wisconsin State Dental Association* Meets semi-annually. Next meeting at Watertown. second Tuesday of January, 1872, *Pres.* Edgar Palmer, Le Crook *Sec.* C. C. Chittenden, Madison.

**Place of meeting fixed by appointment,*

### District Dental Societies of the State of New York.

*First District Dental Society* Meets Monday in New York. Annual meeting, second Wednesday in June : *Pres.* E. A. Bogue, New York : *Sec.* J. S. Latimer,. N. Y.

*Second District Dental Society.* Meets quarterly in Brooklyn. Annual meeting second Tuesday in April. *Pres.* W. S. Elliott : *Rec. Sec.* M, E. Elmandorf. Brooklyn : *Cor.* F. W. Dalbare.

*Third District Dental Society* Meets at Albany semi-annually and annually. *Pres.* D. D. French, Troy : *Sec.* S. J. Andress, Albany.

**Fourth District Dental Society* Meets annually in June at Saratoga. *Pres.* C, A. Elmore, Saratoga Springs. *Sec.* E. S. Pearsall, Fort Edwards.

**Fifth District Dental Society* Meets annually at Saratoga. *Pres.* W. W. Perkins *Sec.* F. D. Neelans.

**Sixth District Dental Society* Annual meeting at Binghampton, second Tuesday in June. *Pres.* R. Walker : *Rec. Sec.* P. L. Foote

**Seventh District Dental Society* Annual meeting is held the last Tuesday in June, at Rochester. *Pres.* G. W. Tripp, Auburn ; *Sec.* J. E. Line, Rochester.

*Eighth District Dental Society* Meets annually on the second Tuesday in May, at TBuffalo : *Bres.* J. C. Gifford, *Sec.* S. A. Freeman, Buffalo.

*he Seventh and Eighth District Societies* Hold a union meeting on the first Tues day of October, alternately at Buffalo and Rochester.

*Place and time of meeting fixed by appointment.

---

The Ohio State Board of Dental Examiners will meet in Columbus on the first Tuesday of Dec. 1874, at 10 o'clock. *Pres.* J. Taft; *Sec.* W. P. Horton.

---

### List of Exchanges.

ST. LOUIS MEDICAL AND SURGICAL JOURNAL.
BOSTON MEDICAL AND SURGICAL JOURNAL.
THE PACIFIC MEDICAL AND SURGICAL JOURNAL, SAN FRANCISCO.
BUFFALO MEDICAL AND SURGICAL JOURNAL.
PHIDADELPHIA MEDICAL AND SURGICAL JOURNAL.
CINCINNATI LANCET AND OBSERVER.
DENTAL COSMOS, PHILADELPHIA,
BRAITHWAITE'S RETROSPECT, N. Y.
THE DENTAL TIMES, PHILADELPHIA.
LADIES' REPOSITORY, CINCINNATI.
HALL'S JOURNAL OF HEALTH, N. Y.
CHICAGO MEDICAL EXAMINER,
THE DETROIT REVIEW OF MEDICINE AND PHARMACY.
MEDICAL RECORD, N. Y.
NASHVILLE JOURNAL OF MEDICINE AND SURGERY.
AMERICAN ECLECTIC MEDICAL REVIEW. N. Y..
AMERICAN JOURNAL OF DENTAL SCIENCE, BALTIMORE.
THE UNITED STATES MEDICAL AND SURGICAL JOURNAL. CHICAGO
NEW ORLEANS JOURNAL OF MEDICINE,
WESTERN JOURNAL OF MEDICINE; INDIANAPOLIS, IND.
AMERICAN AGRICULTURIST, N. Y.
BOSTON JOURNAL OF CHEMISTRY.
JOURNAL OF APPLIED CHEMISTRY, N. Y.
DRUGGISTS' CIRCULAR AND CHEMICAL GAZETTE, N. Y.
CANADA JOURNAL OF DENTAL SCIENCE, MONTREAL.
INDIANA JOURNAL OF MEDICINE,
JOURNAL OF THE GYNÆCOLOGICAL SOCIETY, BOSTON.
MEDICAL AND SURGICAL REPORTER, SALEM, OREGON.
MISSOURI DENTAL JOURNAL, ST. LOUIS.
ECLECTIC MEDICAL JOURNAL, CINCINNATI.

### THE POCKET

### DENTAL REGISTER.

Every dentist with anything like a full practice, and who cares to conduct business in a systematic manner, must have a book in which to record his dental operations.

The large "Registers" that have been used for that purpose, are excellent in their place, and may still be used in connection with such a book as this, if desired; but they are open to several important objections.

Many dentists using them have doubtless experienced the inconvenience of going to the desk after each patient's sitting, to make a record, and have preferred trusting to memory till after office hours. By that time, the exact tooth operated upon has often been forgotten; and sometimes a certain operation has slipped from the mind altogether.

The fillings put in to-day are marked on the same diagram with those inserted a month or a year ago; consequently, after a little while, it is impossible to distinguish between old and recent work.

The space reserved for each account being so large, one is unwilling to spoil a blank to record trifling services, such as extracting or treating a tooth for a patient whom he may never see again; although it often happens afterward that he would be glad to have a knowledge of the service.

In recording operations on the temporary teeth, it is very unsatisfactory to cross out the twelve back teeth of the permanent set, and imagine the bicuspids to be temporary molars. In such cases, much confusion generally follows in the course of a few years.

This Pocket Register is presented with the belief that it is free from these objectionable features.

Each operation may be recorded with pen or pencil, right at the chair, in almost the same time that an appointment is made for another sitting.

Every operation bears its own date, and cannot be taken for a previous or later one.

The space for accounts is so economically used, that one will not hesitate to record the most trivial services. It consists of 224 pages, arranged as follows:

120 pages for small accounts, ten to the page, with blanks for date, name, residence, operation, debit, and credit. Each account is also furnished with a diagram of the teeth for marking operations performed.
24 pages for medium accounts, two to the page, with blanks and diagram, as above.
16 pages for large accounts, one to the page, with blanks and diagram.
16 pages with blanks and diagrams expressly arranged for operations on the temporary teeth, ten accounts to the page.
32 pages for appointments for 64 weeks. These are without date, and may, therefore be used for any time,
16 pages for index and memoranda.

It will be seen that the "Pocket Register" combines Account-book, Record of Operations on the Permanent and Temporary Teeth, Appointment-book, and Memoranda; and that the whole is offered for about the price of an ordinary Engagement book.

It is printed on fine writing paper, bound handsomely in leather, with tuck, or in English cloth, and is of a suitable size for carrying in the breast pocket.

—— PRICE. ——

*In Leather, with Tuck.* .......................................... $1 50
*In English Cloth,* .......................................... 1 25

Sent post-paid, on receipt of price. For sale, wholesale and retail, by

SPENCER & MOORE, Ohio Dental Depot,
Cincinnati, O.

AMERICAN DENTAL ASSOCIATION—Meets on the first Tuesday in Aug., 1875, at Niagara Falls. *Pres.* M. S. Dean, Chicago; *Cor. Sec.* Geo. L, Filed, Detroit; *Rec. Sec.* C. Stodard Smith, Springfield, Ill.

*American Dental Convention* Meets at Saratoga Springs, second Tuesday in Aug., 1874. *Pres.* John Allen, New York City; *Cor. Sec.* A. Tees, Philadelphia, Pa., *Rec. Sec.* C. L. Hurlbut, Springfield, Mass.

*List of Societies Represented in the American Dental Association.*

*American Academy of Dental Science* Meets at Boston, Mass. *Pres.* Joshua Tucker, M. D., of Boston; *Sec.* W. L. Tucker, of Boston; *Cor. Sec.*, E. N. Harris.

*Brooklyn Dental Society* Meets semi-monthly. *Pres.* W. T. Shannon,; *Rec. Sec.* C. P. Crandell; *Cor. Sec.* A. N. Chapman.

*Buffalo Dental Society*Meets second Monday of each month at Buffalo. *Pres.* S. A. FREEMAN; *Rec. Sec.* G. C. DABOLL.

*Boston Dental Institute*—Meets first Tuesday of each month. *Pres.* D. G. WILLIAMS, Buffalo; *Cor. Sec.* D. S. Dickerman.

*California State Dental Association* Meets annually in June. *Pres.* C. C. Knowles, San Francisco; *Rec. Sec.* H. J. Plomteaux, Woodland.

*Cincinnati Dental Society*—Meets Semi-monthly at Cincinnati. *Pres.* H. R. SMITH; *Rec. Sec.* D. C. HAWXHURST.

*Chicago Dental Society* Meets monthly at Chicago. *Pres.* M. S. Dean; *Rec. Sec.* Edgar D. Swain.

*Connecticut Valley Dental Association.* Meets semi-annually, second Tuesday of June and October. *Pres.* H. M. Miller, Westfield, Mass.; *Rec. Sec.* L. C. Taylor, of Holyoke, Mass.

*Connecticut State Dental Association. Pres.* R. C. Dunham, New Britain; *Rec. Sec.* D. E. Lane, Hartford.

*Charleston Dental Association*—Meets second Tuesday of every month; *Pres.* T. F. Chupein; *Sec. and Treas.* B. A. Muckenfuss.

*Delaware Dental Association*—Meets semi-annually. *Pres.* E. W. Haines, Newark; *Sec.* C. R. Jeffries, Wilmington.

*Eastern Indiana Dental Association.*—Meets semi-annually, first Tuesday in May and last Tuesday in October; *Pres.* W. C. Stanley, Dublin, Ind.; *Sec.* J. W. White, Knightstown, Ind. (Place of meeting fixed by appointment.)

*Eastern Ohio Dental Association*—Meets on Tuesday, August 26th, 1873, at Alliance; *Pres.* A. J. Douds; *Sec.* J. M. Porter.

*East Tennessee Dental Association*—Meets annually at Knoxville, Tenn. *Pres.* Wm. W. F. Fowler; *Rec. Sec.* S. M. Prothro.

*Forest City Society of Dental Surgeons*—*Pres.* B. Strickland, Cleveland, *Rec. Sec.* H. L. Ambler, Cleveland; *Cor. Sec.* Chas. Buffett, Cleveland.

*Georgia State Dental Society* Next meeting at Atlanta, July 1873. *Pres.* F. Y. Clark; *Cor. Sec.* J. P. H. Brown, Augusta.

*Harris Dental Association* Meets quarterly on the first Thursday in February, May. August, November. *Pres.* J. A. Martin, Strasburg; *Sec.* Wm. N. Amer, Lancaster,

*Hudson River Association of Dental Surgeons* Meets on the second Wednesdays of May, September and January. *Pres.* L. S. Straw, Newburg, N. Y; *Rec. Sec.* T. W. DuBois, Poughkeepsie, N. Y.

*Hudson Valley Dental Association* Meets monthly at Troy, N. Y. *Pres.* L. C. Wheeler, *Rec. Sec.* S. G. Andres; *Cor. Sec.* S. D. French.

*Illinois State Dental Society* Meets annually. *Pres.* G. S. Miles, Jerseyville, *Sec.* C. R. E. Koch, Chicago. Meets second Tuesday in May, 1875, at Ottowa.

*Indiana State Dental Society*—Meets next year at Indianapolis, on the first Tuesday of June. *Pres.* M. Wells, Indianapolis; *Cor. and Rec. Sec.* P. McDonald, Goshen.

*Iowa State Dental Society*—Meets annually. Next meeting at Davenport, on the second Tuesday of May. *Pres.* L. C. Ingersoll, Keokuk. *Rec. Sec.* E. P. White, of Davenport; *Cor. Sec.* A. J. Redrich, Sioux City.

*Kansas State Dental Society* Meets annually. Next meeting at Leavanworth, first Wednesday in May, 1875. *Pres.* L. C. Wasson; *Sec.* J. D Patterson.

*Kansas and Missouri Valley Dental Association*—Meets first and third Tuesdays of erch month. *Pres.* J. R. Stark; *Sec.* H. C. Massie, Kansas City, Mo.

*Kentucky State Dental Association* Meets annually and semi-monthly, in the City of Louisville. Annual meeting, first Tuesday in June, at 2 o'clock, P. M. Semi-monthly meetings, second and fourth Tuesday nights. G. W. Priest, *Pres.*; B. O. Doyle. *Sec.*, both at Louisville.

*Lake Erie Dental Association* Meets Annually. *Pres.* C. B. Ansart, Oil City, Pa. *Sec.* C. D. Elliot, Franklin, Pa. Next meeting first Tuesday of May, 1874.

*Mad River Dental Association* Meets at Dayton on the first Tuesdays in April and October. *Pres.* S. B. Tizzard, Dayton; *Sec.* A. J. Grosvenor, Troy.

*Maine Dental Society*—Meets in Aug., at Thomaston. *Pres.* E. J. Roberts, Augusta; *Sec.* C. E. Hussey, Biddeford.

*Maryland Association of Dentists* Meets the last Thursday of every month. *Pres.* J. B. Bean; *Sec.* S. M. Field.

*Massachusetts Dental Society*—Meets monthly. *Pres.* J. H. Batchelder, of Salem; *Sec.* C. Wilson; *Cor. Sec.* L. D. Shepard.

*Michigan State Dental Association*—Meets annually second Tuesday of Oct., at Ypsilanti. *Pres.* D. C. Hawxhurst, Ann Arbor; *Rec. & Cor. Sec.* T. R. Perry, Grand Rapids

*Michigan Central Dental Association.* Meets annually at Jackson, Secon Wednesday of January, 1873. *Pres.* C. C. Jenkins, Anu Arbor, *Sec.* W. H. Darrance, Jackson.

*Mississippi Valley Dental Association* Meets annually at Cincinnati, on first Wednesday in March, 1874. *Pres.* S. B. Brown, Ft. Wayne Ind.; *Rec. Sec.* F. Hunter Cin.
*Minnesota Dental Associrtion* Meets annually. Next meeting at Faribault, last Wednesday in June, 1874. *Pres.* J. A. Bowman, Minneapolis; *Sec.* C. D. Montreville, St. Paul.
*Missouri Dental Association* Meets on the first Tuesday of June, in St. Louis. *Pres.* C. W. Rivers, St. Louis; *Sec.* W. H. Evans, St. Louis.
*Missouri Valley Dental Association** Meets on the second Tuesday in July and January. *Pres.* A. S. Billings, Omaha, Neb.; *Sec. and Treas.* J. F. Sanborn, Tabor, Iowa.
*Merrimac Valley Dental Association* Meets semi-annually first Tuesday of Oct. and May.* *Pres.* L. H. Kidder, Mass; *Rec. Sec.* A. M. Dudley, of Lowell; *Cor. Sec.* D. T. Porter, Lawrence, Mass.
*Memphis Dental Society*—Meets on the first Tuesday of each month. *Pres.* W. M. Arrington; *Sec.* V. F. Elliott; *Treas.* C. L. Harris.
*Nashville Dental ssociation*--Meets on the second Tuesday of each month. *Pres* J. C. Ross; *Sec.* R. R. Freeman; *Cor. Sec.* S. J. Cobb.
*New Jersey State Dental Society*—Meets annually. Next meeting at Mount Holly, on the second Tuesday in July, 1874. *Pres.* J. W. Cosad, Jersey City; *Sec.* J. W. Scarborough, Lambertville.
*New York State Dental Society*--Meets at Albany on the last Wednesday of June next. *Pres.* W. C. Barret, Warsaw, N. Y.; *Sec.* Chas. Barnes, of Syracuse, *Cor. Sec.* W. S. Elliott, Goshen; *Rec. Sec.* Charles Barnes, Syracuse,
*New Haven Dental Society*--Meets on the first Wednesday of each month at New Haven. *Pres.* J. T. Metcalf, *Rec and Cor. Sec.* C. L. Smith, of New Haven.
*New Orleans Dental Society* Meets monthly. *Pres.* ——————; *Rec. Sec.* A. F McLane.
*Northern Ohio Dental Association.* Meets annually, second Tuesday of June, 1873, at Put-in Bay. *Pres.* J. A. Robinson, Cleveland; *Rec. Sec.* A. F. Price, Fremont.
*Northern Iowa Dental Association*—Meets annually, on the second Tuesday in June, 1873. Next meeting at Waterloo; *Pres.* J. N. Abbott, Lancaster, *Rec. Sec.* A. V. Eaton, Anamosa; *Cor. Sec.* A. R. Mason, Waterloo.
*Ohio College Dental Association*--Meets annually Feb. 25. at Cincinnati. *Pres.* Jas. Leslie, Cin'ti.; *Rec. Sec.* H. A. Smith, of Cincinnati.
*Ohio State Dental Association*--Meets annually at Columbus. Next meeting, first Wednesday of December. *Pres.* H. A. Smith, Cincinnati; *Rec. Sec.* J. M. Porter, Massilon; *Cor. Sec.* C. R. Butler, Cleveland, O.
*Odontographic Society of Pennsylvania*--Meets monthly in Philadelphia. Next meeting, Wednesday, Sept. 1st. *Pres.* C. T. Stellwagen; *Rec. Sec.* E. L. Hewitt.
*Odontological Society of New York City* Meets the second Tuesday of each monh. *Pres.* C. E. Francis; *Rec. Sec.* W. E. Hong.
*Old Colony Dental Association* Meets quarterly.* *Pres.* C. G. Davis; *Rec. Sec.* L. W. Puffer, North Bridgewater, Mass; *Cor. Sec.* N. C. Fowler, Yarmouth, Mass.
*Ohio Valley Dental Association* Meets annually, in N. E. Ky; this year in Paris, Ky. Tuesday, Sept. 9th.
*Oregon State Dental Society* Meets annually. *Pres.* J. H. Hatch, Portland; *Rec. Sec.* J. R. Cardwell, Portland.
*Pennsylvania State Dental Society* Meets next at Wilkesbarre, second Tuesday of July, 1874. *Pres.* G. W. Klump, Williamsport; *Rec. Sec.* R. H. Moffit, Harrisburg *Cor. Sec.* S. Welchens, Lancaster.
*Poughkeepsie Dental Society* Meets monthly. *Pres.* J. H. Mann; *Sec.* H. F. Clark
*Pennsylvania Association of Dental Surgery* Meets monthly in Philadelphia. *Pres.* Spencer Roberts; *Sec.* E. R. Pettit.
*San Francisco Dental Association* Meets monthly. *Pres.* C. C. Knowles; *Cor. Sec.* H. E. Knox; *Rec. Sec.* Wm. Dutch.
*St. Louis Dental Society* Meets monthly. *Pres.* W. H. Morrison; *Sec.* A. H. Fuller.
*Susquehanna Dental Association* Meets annually.* *Pres.* J. L. Fordham: *Rec. Sec.* Wm. Nertz. Meets Nov. 11, 1874, at Lewisburg.
*Society of Dental Surgeons of the City of New York* Meets semi-monthly in New York. *Pres.* B. F. Franklin: *Rec. Sec.* J. S. Latimer; *Cor. Sec.* E. H. Burras.
**Southern Dental Association* Meets annually. Next meeting the last Tuesday of July, 1873, at Baltimore, Md. *Pres.* H. M. Grant Virginia.: *Rec. Sec.* J. F Thompson Va
*South Carolina State Dental Association* Meets semi-annually in May and Nov. *Pres.* T. F. Chupein, Charleston; *Sec.* J. W. Norwood, Greenville; *Cor. Sec.* C. C. Patrick, Charleston, S. C. Next meeting at Columbia.
*Tennessee Dental Association* Meets semi-annually. Next meeting in Nashville, on the 4th Wednesday in June. 1875. *Pres.* E. S. Chisholm; *Sec.* J. S. King; *Cor. Sec.* S. J. Cobb.
*Virginia State Dental Association* Meets in Richmond, first Thursday in Nov. of each year. *Pres.* H. M. Grant, Abington: *Sec.* G. F. Kersee, Richmond.
*Washington, City Dental Society* Meets annually. *Pres.* R. F. Hunt, *Sec.* Dr. Evans.
*Wabash Valley Dental Association* Meets annually. Next meeting at Wayne, third Tuesday in March, 1872. *Pres.* A. B. Cunningham, Attica: *Sec,* S. B. Brown, Fort Wayne.
* *Wisconsin State Dental Association* Meets semi-annually. Next meeting at Watertown. second Tuesday of January, 1872, *Pres.* Edgar Palmer, Le Crock *Sec.* C. C. Chittenden, Madison.

**Place of meeting fixed by appointment,*

### District Dental Societies of the State of New York.

*First District* Meets second and fourth Wednesday evenings of each month. Annual meeting in New York, first Tuesday in June. *Pres.* R. M. Gage; *Rec. Sec.* W. E. Hoag, 331 Madison ave., New York.
*Second District.* Annual meeting in Brooklyn, first Monday in April. *Pres.* W. S. Elliott; *Rec. Sec.* M. E. Elmandorf. Brooklyn; *Cor. Sec.* F. W. Dalbare.
*Third District.* Meets semi-annually on second Tuesday in January and July. Annual meeting at Albany on second Tuesday in January. *Pres.* J. C. Austin, Albany; *Rec. Sec.* S. J. Andress, Troy; *Cor. Sec.* D. H. Young, Troy.
*Fourth District.* Meets semi-annually. Meets annually at Saratoga on second Tuesday in June. *Pres.* C, A. Elmore, Ft. Edward; *Rec. Sec.* E. S. Pearsall, Saratoga.
*Fifth District.* Meets semi-annually. Annual meeting at Syracuse. second Tuesday in June. *Pres.* J. D. Huntington, Watertown; *Rec. Sec.* F. D. Nellis, Syracuse; *Cor. Sec.* J. S. Marshall, Syracuse.
*Sixth District.* Meets semi-annually. Annual meeting at Binghampton on second Tuesday in June. *Pres.* Frank B. Darby, Oswego; *Rec. Sec.* F. E. Perry, Norwich; *Cor. Sec.* A. M. Holmes, Morrisville.
*Seventh District.* Meets semi-annually. Annual meeting at Rochester first Tuesday in June. *Pres.* H. S. Miller; *Rec. Sec.* J. E. Line, Rochester.
*Eighth District.* Meets semi-annually. Annual on second Tuesday in May. *Pres.* G. C. Daboll, *Rec. Sec.* S. A. Freeman, *Cor. Sec.* T. G. Lewis, Buffalo.
*Buffalo Dental Association.* Meets on second Tuesday evening of each month. Annual meeting in June. *Pres.* S. A. Freeman; *Sec.* W. H. Barrows.

The Ohio State Board of Dental Examiners will meet in Columbus on the first Tuesday of Dec. 1874, at 10 o'clock. *Pres.* J. Taft; *Sec.* W. P. Horton.

### List of Exchanges.

ST. LOUIS MEDICAL AND SURGICAL JOURNAL.
BOSTON MEDICAL AND SURGICAL JOURNAL.
THE PACIFIC MEDICAL AND SURGICAL JOURNAL, SAN FRANCISCO.
PHIDADELPHIA MEDICAL AND SURGICAL JOURNAL.
CINCINNATI LANCET AND OBSERVER.
DENTAL COSMOS, PHILADELPHIA.
LADIES' REPOSITORY, CINCINNATI.
HALL'S JOURNAL OF HEALTH, N. Y.
CHICAGO MEDICAL EXAMINER,
THE DETROIT REVIEW OF MEDICINE AND PHARMACY.
MEDICAL RECORD, N. Y.
NASHVILLE JOURNAL OF MEDICINE AND SURGERY.
AMERICAN JOURNAL OF DENTAL SCIENCE, BALTIMORE.
THE UNITED STATES MEDICAL AND SURGICAL JOURNAL, CHICAGO
NEW ORLEANS JOURNAL OF MEDICINE,
AMERICAN AGRICULTURIST, N. Y.
BOSTON JOURNAL OF CHEMISTRY.
JOURNAL OF APPLIED CHEMISTRY, N. Y.
CANADA JOURNAL OF DENTAL SCIENCE, MONTREAL.
INDIANA JOURNAL OF MEDICINE,
MEDICAL AND SURGICAL REPORTER, SALEM, OREGON
MISSOURI DENTAL JOURNAL, ST. LOUIS
ECLECTIC MEDICAL JOURNAL, CINCINNATI.

THE POCKET

### DENTAL REGISTER.

Every dentist with anything like a full practice, and who cares to conduct business in a systematic manner, must have a book in which to record his dental operations.

The large "Registers" that have been used for that purpose, are excellent in their place, and may still be used in connection with such a book as this, if desired; but they are open to several important objections.

Many dentists using them have doubtless experienced the inconvenience of going to the desk after each patient's sitting, to make a record, and have preferred trusting to memory till after office hours. By that time, the exact tooth ope ated upon has often been forgotten; and sometimes a certain operation has slipped from the mind altogether.

The fillings put in to-day are marked on the same diagram with those inserted a month or a year ago; consequently, after a little while, it is impossible to distinguish between old and recent work.

The space reserved for each account being so large, one is unwilling to spoil a blank to record trifling services, such as extracting or treating a tooth for a patient whom he may never see again; although it often happens afterward that he would be glad to have a knowledge of the service.

In recording operations on the temporary teeth, it is very unsatisfactory to cross out the twelve back teeth of the permanent set, and imagine the bicuspids to be temporary molars. In such cases, much confusion generally follows in the course of a few years.

This POCKET REGISTER is presented with the belief that it is free from these objec. tionable features.

Each operation may be recorded with pen or pencil, right at the chair, in almost the same time that an appointment is made for another sitting.

Every operation bears its own date, and cannot be taken for a previous or later one.

The space for accounts is so economically used, that one will not hesitate to record the most trivial services. It consists of 224 pages, arranged as follows:

120 pages for small account , ten to the page, with blanks for date, name, residence, operation, debit, and credit. Each account is also furnished with a diagram of the teeth for marking operations performed.
24 pages for medium accounts, two to the page, with blanks and diagram, as above.
16 pages for large accounts, one to the page, with blanks and diagram.
16 pages with blanks and diagrams expressly arranged for operations on the temporary teeth, ten accounts to the page.
32 pages for appointments for 64 weeks. These are without date, and may, therefore, be used for any time,
16 pages for index and memoranda.

It will be seen that the "Pocket Register" combines Account-book, Record of Operations on the Permanent and Temporary Teeth, Appointment-book, and Memoranda; and that the whole is offered for about the price of an ordinary Engagement book.

It is printed on fine writing paper, bound handsomely in leather, with tuck, or in English cloth, and is of a suitable size for carrying in the breast pocket,

—— PRICE. ——

*In Leather, with Tuck.* .................................................. $1 50
*In English Cloth,* .................................................. 1 25

Sent post-paid, on receipt of price. For sale, wholesale and retail, by

SPENCER & MOORE, Ohio Dental Depot,
Cincinnati, O,

AMERICAN DENTAL ASSOCIATION—Meets on the first Tuesday in Aug., 1874, at Detroit. *Pres.* T. L. Buckingham, Philad.; *Cor. Sec.* J. Taft, Cincinnati, O.; *Rec. Sec.* M. S. Dean, Chicago.

*American Dental Convention* Meets at Saratoga Springs, second Tuesday in Aug., 1874. *Pres.* John Allen, New York City; *Cor. Sec.* A. Tees, Philadelphia, Pa., *Rec. Sec.* C. L. Hurlbut, Springfield, Mass.

*List of Societies Represented in the American Dental Association.*

*American Academy of Dental Science* Meets at Boston, Mass. *Pres.* Joshua Tucker, M. D., of Boston; *Sec.* W. L. Tucker, of Boston; *Cor. Sec.*, E. N. Harris.

*Brooklyn Dental Society* Meets semi-monthly. *Pres.* W. T. Shannon, ; *Rec. Sec.* C. P. Crandell; *Cor. Sec.* A. N. Chapman.

*Buffalo Dental Society* Meets second Monday of each month at Buffalo. *Pres.* S. A. FREEMAN; *Rec. Sec.* G. C. DABOLL.

*Boston Dental Institute*—Meets first Tuesday of each month. *Pres.* D. G. WILLIAMS, Buffalo; *Cor. Sec.* D. S. Dickerman.

*California State Dental Association* Meets annually in June. *Pres.* C. C. Knowles, San Francisco; *Rec. Sec.* H. J. Plomteaux, Woodland.

*Cincinnati Dental Society*—Meets Semi-monthly at Cincinnati. *Pres.* H. R. SMITH; *Rec. Sec.* D. C. HAWXHURST.

*Chicago Dental Society* Meets monthly at Chicago. *Pres.* M. S. Dean; *Rec. Sec.* Edgar D. Swain.

*Connecticut Valley Dental Association.* Meets semi-annually, second Tuesday of June and October. *Pres.* H. M. Miller, Westfield, Mass.; *Rec. Sec.* L. C. Taylor, of Holyoke, Mass.

*Connecticut State Dental Association.* *Pres.* R. C. Dunham, New Britain; *Rec. Sec.* D. E. Lane, Hartford.

*Charleston Dental Association*—Meets second Tuesday of every month; *Pres.* T. F. Chupein; *Sec. and Treas.* B. A. Muckenfuss.

*Delaware Dental Association*—Meets semi-annually. *Pres.* E. W. Haines, Newark; *Sec.* C. R. Jeffries, Wilmington.

*Eastern Indiana Dental Association.*—Meets semi-annually, first Tuesday in May and last Tuesday in October; *Pres.* W. C. Stanley, Dublin, Ind.; *Sec.* J. W. White, Knightstown, Ind. (Place of meeting fixed by appointment.)

*Eastern Ohio Dental Association*—Meets on Tuesday, August 26th, 1873, at Alliance; *Pres.* A. J. Douds; *Sec.* J. M. Porter.

*East Tennessee Dental Association*—Meets annually at Knoxville, Tenn. *Pres.* Wm. W. F. Fowler; *Rec. Sec.* S. M. Prothro.

*Forest City Society of Dental Surgeons*—*Pres.* B. Strickland, Cleveland, *Rec. Sec.* H. L. Ambler, Cleveland; *Cor. Sec.* Chas. Buffett, Cleveland.

*Georgia State Dental Society* Next meeting at Atlanta, July 1873. *Pres.* F. Y. Clark; *Cor. Sec.* J. P. H. Brown, Augusta.

*Harris Dental Association* Meets quarterly on the first Thursday in February, May, August, November. *Pres.* J. A. Martin, Strasburg; *Sec.* Wm. N. Amer, Lancaster.

*Hudson River Association of Dental Surgeons* Meets on the second Wednesdays of May, September and January. *Pres.* L. S. Straw, Newburg, N. Y; *Rec. Sec.* T. W. DuBois, Poughkeepsie, N. Y.

*Hudson Valley Dental Association* Meets monthly at Troy, N. Y. *Pres.* L. C. Wheeler, *Rec. Sec.* S. G. Andres; *Cor. Sec.* S. D. French.

*Illinois State Dental Society* Meets annually. *Pres.* G. S. Miles, Jerseyville, *Sec.* C. R. E. Koch, Chicago. Meets second Tuesday in May, 1875, at Ottowa.

*Indiana State Dental Society*—Meets next year at Indianapolis, on the first Tuesday of June. *Pres.* M. Wells, Indianapolis; *Cor. and Rec. Sec.* P. McDonald, Goshen.

*Iowa State Dental Society*—Meets annually. Next meeting at Davenport, on the second Tuesday of May. *Pres.* L. C. Ingersoll, Keokuk. *Rec. Sec.* E. P. White, of Davenport; *Cor. Sec.* A. J. Redrich, Sioux City.

*Kansas State Dental Society* Meets annually. Next meeting at Leavanworth, first Wednesday in May, 1875. *Pres.* L. C. Wasson; *Sec.* J. D Patterson.

*Kansas and Missouri Valley Dental Association*—Meets first and third Tuesdays of erch month. *Pres.* J. R. Stark; *Sec.* H. C. Massie, Kansas City, Mo.

*Kentucky State Dental Association* Meets annually and semi-monthly, in the City of Louisville. Annual meeting, first Tuesday in June, at 2 o'clock, P. M. Semi-monthly meetings, second and fourth Tuesday nights. G. W. Priest, *Pres.*; B. O. Doyle. *Sec.*, both at Louisville.

*Lake Erie Dental Association* Meets Annually. *Pres.* C. B. Ansart, Oil City, Pa. *Sec.* C. D. Elliot, Franklin, Pa. Next meeting first Tuesday of May, 1874.

*Mad River Dental Association* Meets at Dayton on the first Tuesdays in April and October. *Pres.* S. B. Tizzard, Dayton; *Sec.* A. J. Grosvenor, Troy.

*Maine Dental Society*—Meets in Aug., at Thomaston. *Pres.* E. J. Roberts, Augusta; *Sec.* C. E. Hussey, Biddeford.

*Maryland Association of Dentists* Meets the last Thursday of every month. *Pres.* J. B. Bean; *Sec.* S. M. Field.

*Massachusetts Dental Society*—Meets monthly. *Pres.* J. H. Batchelder, of Salem; *Sec.* C. Wilson; *Cor. Sec.* L. D. Shepard.

*Michigan State Dental Association*—Meets annually second Tuesday of Oct., at Ypsilanti. *Pres.* D. C. Hawxhurst, Ann Arbor; *Rec. & Cor. Sec.* T. R. Perry, Grand Rapids

*Michigan Central Dental Association.* Meets annually at Jackson, Secon Wednesday of January, 1873. *Pres.* C. C. Jenkins, Ann Arbor, *Sec.* W. H. Darrance, Jackson.

*Mississippi Valley Dental Association* Meets annually at Cincinnati, on first Wednesday in March, 1874. *Pres.* S. B. Brown, Ft. Wayne Ind.; *Rec. Sec.* F. Hunter Cin.
*Minnesota Dental Associrtion* Meets annually. Next meeting at Faribault, last Wednesday in June, 1874. *Pres.* J. A. Bowman, Minneapolis; *Sec.* C. D. Montreville, St. Paul.
*Missouri Dental Association* Meets on the first Tuesday of June, in St. Louis. *Pres.* C. W. Rivers, St. Louis; *Sec.* W. H. Evans, St. Louis.
*Missouri Valley Dental Association** Meets on the second Tuesday in July and January. *Pres.* A. S. Billings, Omaha, Neb.; *Sec. and Treas.* J. F. Sanborn, Tabor, Iowa.
*Merrimac Valley Dental Association* Meets semi-annually first Tuesday of Oct. and May.* *Pres.* L. H. Kidder, Mass; *Rec. Sec.* A. M. Dudley, of Lowell; *Cor. Sec.* D. T. Porter, Lawrence, Mass.
*Memphis Dental Society*—Meets on the first Tuesday of each month. *Pres.* W. M. Arrington; *Sec.* V. F. Elliott; *Treas.* C. L. Harris.
*Nashville Dental ssociation*--Meets on the second Tuesday of each month. *Pres* J. C. Ross; *Sec.* R. R. Freeman; *Cor. Sec.* S. J. Cobb.
*New Jersey State Dental Society*—Meets annually. Next meeting at Mount Holly, on the second Tuesday in July, 1874. *Pres.* J. W. Cosad, Jersey City; *Sec.* J. W. Scarborough, Lambertville.
*New York State Dental Society*--Meets at Albany on the last Wednesday of June next. *Pres.* W. C. Barret, Warsaw, N. Y.; *Sec.* Chas. Barnes, of Syracuse, *Cor. Sec.* W. S. Elliott, Goshen; *Rec. Sec.* Charles Barnes, Syracuse,
*New Haven Dental Society*--Meets on the first Wednesday of each month at New Haven. *Pres.* J. T. Metcalf, *Rec and Cor. Sec.* C. L. Smith, of New Haven.
*New Orleans Dental Society* Meets monthly. *Pres.* ————————; *Rec. Sec.* A. F McLane.
*Northern Ohio Dental Association.* Meets annually, second Tuesday of June, 1873, at Put-in Bay. *Pres.* J. A. Robinson, Cleveland; *Rec. Sec.* A. F. Price, Fremont.
*Northern Iowa Dental Association*—Meets annually, on the second Tuesday in June, 1873. Next meeting at Waterloo; *Pres.* J. N. Abbott, Lancaster, *Rec. Sec.* A. V. Eaton, Anamosa; *Cor. Sec.* A. R. Mason, Waterloo.
*Ohio College Dental Association*—Meets annually Feb. 25. at Cincinnati. *Pres.* Jas. Leslie, Cin'ti.; *Rec. Sec.* H. A. Smith, of Cincinnati.
*Ohio State Dental Association*—Meets annually at Columbus. Next meeting, first Wednesday of December. *Pres.* H. A. Smith, Cincinnati; *Rec. Sec.* J. M. Porter, Massilon; *Cor. Sec.* C. R. Butler, Cleveland, O.
*Odontographic Society of Pennsylvania*—Meets monthly in Philadelphia. Next meeting, Wednesday, Sept. 1st. *Pres.* C. T. Stellwagen; *Rec. Sec.* E. L. Hewitt.
*Odontological Society of New York City* Meets the second Tuesday of each monh. *Pres.* C. E. Francis; *Rec. Sec.* W. E. Hong.
*Old Colony Dental Association* Meets quarterly.* *Pres.* C. G. Davis; *Rec. Sec.* L. W. Puffer, North Bridgewater, Mass; *Cor. Sec.* N. C. Fowler, Yarmouth, Mass.
*Ohio Valley Dental Association* Meets annually, in N. E. Ky; this year in Paris, Ky. Tuesday, Sept. 9th.
*Oregon State Dental Society* Meets annually. *Pres.* J. H. Hatch, Portland; *Rec. Sec.* J. R. Cardwell, Portland.
*Pennsylvania State Dental Society* Meets next at Wilkesbarre, second Tuesday of July, 1874. *Pres.* G. W. Klump, Williamsport; *Rec. Sec.* R. H. Moffit, Harrisburg *Cor. Sec.* S. Welchens, Lancaster.
*Poughkeepsie Dental Society* Meets monthly. *Pres.* J. H. Mann; *Sec.* H. F. Clark
*Pennsylvania Association of Dental Surgery* Meets monthly in Philadelphia. *Pres.* Spencer Roberts; *Sec.* E. R. Pettit.
*San Francisco Dental Association* Meets monthly. *Pres.* C. C. Knowles; *Cor. Sec.* H. E. Knox; *Rec. Sec.* Wm. Dutch.
*St. Louis Dental Society* Meets monthly. *Pres.* W. H. Morrison; *Sec.* A. H. Fuller.
*Susquehanna Dental Association* Meets annually.* *Pres.* J. L. Fordham: *Rec. Sec.* G. W. Klump, Williamsport. Meets Nov. 12, 1873.
*Society of Dental Surgeons of the City of New York* Meets semi-monthly in New York. *Pres.* B. F. Franklin: *Rec. Sec.* J. S. Latimer; *Cor. Sec.* E. H. Burras.
**Southern Dental Association* Meets annually. Next meeting the last Tuesday of July, 1873, at Baltimore, Md. *Pres.* H. M. Grant Virginia.: *Rec. Sec.* J. F Thompson Va.
*South Carolina State Dental Association* Meets semi-annually in May and Nov. *Pres.* T. F. Chupein, Charleston; *Sec.* J. W. Norwood, Greenville.
*Tennessee Dental Association* Meets semi-annually. Next meeting in Nashville, on the 4th Wednesday in June. 1875. *Pres.* E. S. Chisholm; *Sec.* J. S. King; *Cor. Sec.* S. J. Cobb.
*Virginia State Dental Association* Meets in Richmond, first Thursday in Nov. of each year. *Pres.* H. M. Grant, Abington: *Sec.* G. F. Kersee, Richmond.
*Washington, City Dental Society* Meets annually. *Pres.* R. F. Hunt, *Sec.* Dr. Evans.
*Wabash Valley Dental Association* Meets annually. Next meeting at Wayne, third Tuesday in March, 1872. *Pres.* A. B. Cunningham, Attica: *Sec.* S. B. Brown, Fort Wayne.
* *Wisconsin State Dental Association* Meets semi-annually. Next meeting at Watertown. second Tuesday of January, 1872, *Pres.* Edgar Palmer, Le Crook *Sec.* C. C. Chittenden, Madison.

**Place of meeting fixed by appointment,*

### District Dental Societies of the State of New York.

*First District Dental Society* Meets Monday in New York. Annual meeting, second Wednesday in June: *Pres.* E. A. Bogue, New York: *Sec.* J. S. Latimer, N. Y.

*Second District Dental Society.* Meets quarterly in Brooklyn. Annual meeting second Tuesday in April. *Pres.* W. S. Elliott: *Rec. Sec.* M. E. Elmandorf. Brooklyn: *Cor.* F. W. Dalbare.

*Third District Dental Society* Meets at Albany semi-annually and annually. *Pres.* D. D. French, Troy: *Sec.* S. J. Andress, Albany.

**Fourth District Dental Society* Meets annually in June at Saratoga. *Pres.* C. A. Elmore, Saratoga Springs. *Sec.* E. S. Pearsall, Fort Edwards.

**Fifth District Dental Society* Meets annually at Saratoga. *Pres.* W. W. Perkins *Sec.* F. D. Neelans.

**Sixth District Dental Society* Annual meeting at Binghampton, second Tuesday in June. *Pres.* R. Walker: *Rec. Sec.* P. L. Foote

**Seventh District Dental Society* Annual meeting is held the last Tuesday in June, at Rochester. *Pres.* G. W. Tripp, Auburn; *Sec.* J. E. Line, Rochester.

*Eighth District Dental Society* Meets annually on the second Tuesday in May, at Buffalo: *Pres.* G. C. Daboll, *Sec.* S. A. Freeman, Buffalo.

*The Seventh and Eighth District Societies* Hold a union meeting on the first Tuesday of October, alternately at Buffalo and Rochester.

*Place and time of meeting fixed by appointment.

---

The Ohio State Board of Dental Examiners will meet in Columbus on the first Tuesday of Dec. 1874, at 10 o'clock. *Pres.* J. Taft; *Sec.* W. P. Horton.

---

### List of Exchanges.

ST. LOUIS MEDICAL AND SURGICAL JOURNAL.
BOSTON MEDICAL AND SURGICAL JOURNAL.
THE PACIFIC MEDICAL AND SURGICAL JOURNAL, SAN FRANCISCO.
PHIDADELPHIA MEDICAL AND SURGICAL JOURNAL.
CINCINNATI LANCET AND OBSERVER.
DENTAL COSMOS, PHILADELPHIA.
LADIES' REPOSITORY, CINCINNATI.
HALL'S JOURNAL OF HEALTH, N. Y.
CHICAGO MEDICAL EXAMINER.
THE DETROIT REVIEW OF MEDICINE AND PHARMACY.
MEDICAL RECORD, N. Y.
NASHVILLE JOURNAL OF MEDICINE AND SURGERY.
AMERICAN JOURNAL OF DENTAL SCIENCE, BALTIMORE.
THE UNITED STATES MEDICAL AND SURGICAL JOURNAL, CHICAGO
NEW ORLEANS JOURNAL OF MEDICINE.
AMERICAN AGRICULTURIST, N. Y.
BOSTON JOURNAL OF CHEMISTRY.
JOURNAL OF APPLIED CHEMISTRY, N. Y.
CANADA JOURNAL OF DENTAL SCIENCE, MONTREAL.
INDIANA JOURNAL OF MEDICINE.
MEDICAL AND SURGICAL REPORTER, SALEM, OREGON.
MISSOURI DENTAL JOURNAL, ST. LOUIS.
ECLECTIC MEDICAL JOURNAL, CINCINNATI.

THE POCKET

### DENTAL REGISTER,

Every dentist with anything like a full practice, and who cares to conduct business in a systematic manner, must have a book in which to record his dental operations.

The large "Registers" that have been used for that purpose, are excellent in their place, and may still be used in connection with such a book as this, if desired; but they are open to several important objections.

Many dentists using them have doubtless experienced the inconvenience of going to the desk after each patient's sitting, to make a record, and have preferred trusting to memory till after office hours. By that time, the exact tooth ope ated upon has often been forgotten; and sometimes a certain operation has slipped from the mind altogether.

The fillings put in to-day are marked on the same diagram with those inserted a month or a year ago; consequently, after a little while, it is impossible to distinguish between old and recent work.

The space reserved for each account being so large, one is unwilling to spoil a blank to record trifling services, such as extracting or treating a tooth for a patient whcm he may never see again; although it often happens afterward that he would be glad to have a knowledge of the service.

In recording operations on the temporary teeth, it is very unsatisfactory to cross out the twelve back teeth of the permanent set, and imagine the bicuspids to be temporary molars. In such cases, much confusion generally follows in the course of a few years.

This Pocket Register is presented with the belief that it is free from these objec. tionable features.

Each operation may be recorded with pen or pencil, right at the chair, in almost the same time that an appointment is made for another sitting.

Every operation bears its own date, and cannot be taken for a previous or later one.

The space for accounts is so economically used, that one will not hesitate to record the most trivial services. It consists of 224 pages, arranged as follows:

120 pages for small account , ten to the page, with blanks for date, name, residence. operation, debit, and credit. Each account is also furnished with a diagram of the teeth for marking operations performed.
24 pages for medium accounts, two to the page, with blanks and diagram, as above.
16 pages for large accounts, one to the page, with blanks and diagram.
16 pages with blanks and diagrams expressly arranged for operations on the temporary teeth, ten accounts to the page.
32 pages for appointments for 64 weeks. These are without date, and may, therefore be used for any time,
16 pages for index and memoranda.

It will be seen that the "Pocket Register" combines Account-book, Record of Operations on the Permanent and Temporary Teeth, Appointment-book, and Memoranda; and that the whole is offered for about the price of an ordinary Engagement book.

It is printed on fine writing paper, bound handsomely in leather, with tuck, or in English cloth, and is of a suitable size for carrying in the breast pocket.

—— PRICE. ——

*In Leather, with Tuck.* .............................................. $1 50
*In English Cloth,* .............................................. 1 25

Sent post-paid, on receipt of price. For sale, wholesale and retail, by

SPENCER & MOORE, Ohio Dental Depot,
Cincinnati, O,

AMERICAN DENTAL ASSOCIATION—Meets on the first Tuesday in Aug., 1874, at Detroit. *Pres.* T. L. Buckingham, Philad.; *Cor. Sec.* J. Taft, Cincinnati, O.; *Rec. Sec.* M. S. Dean, Chicago.

*American Dental Convention* Meets at Saratoga Springs, second Tuesday in Aug., 1874. *Pres.* John Allen, New York City; *Cor. Sec.* A. Tees, Philadelphia, Pa., *Rec. Sec.* C. L. Hurlbut, Springfield, Mass.

*List of Societies Represented in the American Dental Association.*

*American Academy of Dental Science* Meets at Boston, Mass. *Pres.* Joshua Tucker, M. D., of Boston; *Sec.* W. L. Tucker, of Boston; *Cor. Sec.*, E. N. Harris.

*Brooklyn Dental Society* Meets semi-monthly. *Pres.* W. T. Shannon,; *Rec. Sec.* C. P. Crandell; *Cor. Sec.* A. N. Chapman.

*Buffalo Dental Society* Meets second Monday of each month at Buffalo. *Pres.* S. A. FREEMAN; *Rec. Sec.* G. C. DABOLL.

*Boston Dental Institute*—Meets first Tuesday of each month. *Pres.* D. G. WILLIAMS; *Cor. Sec.* D. S. DICKERMAN.

*California State Dental Association* Meets annually in June. *Pres.* C. C. Knowles, San Francisco; *Rec. Sec.* H. J. Plomteaux, Woodland.

*Cincinnati Dental Society*—Meets Semi-monthly at Cincinnati. *Pres.* H. R. SMITH; *Rec. Sec.* D. C. HAWXHURST.

*Chicago Dental Society* Meets monthly at Chicago. *Pres.* M. S. Dean; *Rec. Sec.* Edgar D. Swain.

*Connecticut Valley Dental Association.* Meets semi-annually, second Tuesday of June and October. *Pres.* H. M. Miller, Westfield, Mass.; *Rec. Sec.* L. C. Taylor, of Holyoke, Mass.

*Connecticut State Dental Association.* *Pres.* R. C. Dunham, New Britain; *Rec. Sec.* D. E. Lane, Hartford.

*Charleston Dental Association*—Meets second Tuesday of every month; *Pres.* T. F. Chupein; *Sec. and Treas.* B. A. Muckenfuss.

*Delaware Dental Association*—Meets semi-annually. *Pres.* E. W. Haines, Newark; *Sec.* C. R. Jeffries, Wilmington.

*Eastern Ohio Dental Association*—Meets on Tuesday, August 26th, 1873, at Alliance; *Pres.* A. J. Douds; *Sec.* J. M. Porter.

*East Tennessee Dental Association*—Meets annually at Knoxville, Tenn. *Pres.* Wm. W. F. Fowler; *Rec. Sec.* S. M. Prothro.

*Forest City Society of Dental Surgeons*—*Pres.* B. Strickland, Cleveland, *Rec. Sec.* H. L. Ambler, Cleveland; *Cor. Sec.* Chas. Buffett, Cleveland.

*Georgia State Dental Society* Next meeting at Atlanta, July 1873. *Pres.* F. Y. Clark; *Cor. Sec.* J. P. H. Brown, Augusta.

*Harris Dental Association* Meets quarterly on the first Thursday in February, May, August, November. *Pres.* J. A. Martin, Strasburg; *Sec.* Wm. N. Amer, Lancaster.

*Hudson River Association of Dental Surgeons* Meets on the second Wednesdays of May, September and January. *Pres.* L. S. Straw, Newburg, N. Y; *Rec. Sec.* T. W. DuBois, Poughkeepsie, N. Y.

*Hudson Valley Dental Association* Meets monthly at Troy, N. Y. *Pres.* L. C. Wheeler, *Rec. Sec.* S. G. Andres; *Cor. Sec.* S. D. French.

*Illinois State Dental Society* Meets annually. *Pres.* C. Stoddard Smith, Springfield; *Sec.* C. R. E. Koch, Chicago. Meets second Tuesday in May, 1874, at Jacksonville.

*Indiana State Dental Society*—Meets next year at Indianapolis, on the first Tuesday of June. *Pres.* M. Wells, Indianapolis; *Cor. and Rec. Sec.* P. McDonald, Goshen.

*Iowa State Dental Society*—Meets annually. Next meeting at Davenport, on the second Tuesday of May. *Pres.* L. C. Ingersoll, Keokuk. *Rec. Sec.* E. P. White, of Davenport; *Cor. Sec.* A. J. Redrich, Sioux City.

*Kansas State Dental Society* Meets semi-annually. Next meeting at Topeka, Sept. 12, 1873. *Pres.* Dr. Griswold; *Sec.* A. H. Thompson.

*Kansas and Missouri Valley Dental Association*—Meets first and third Tuesdays of erch month. *Pres.* J. R. Stark; *Sec.* H. C. Massie, Kansas City, Mo.

*Kentucky State Dental Association* Meets annually and semi-monthly, in the City of Louisville. Annual meeting, first Tuesday in June, at 2 o'clock, P. M. Semi-monthly meetings, second and fourth Tuesday nights. G. W. Priest, *Pres.*; B. O. Doyle. *Sec.*, both at Louisville.

*Lake Erie Dental Association* Meets Annually. *Pres.* C. B. Ansart, Oil City, Pa. *Sec.* C. D. Elliot, Franklin, Pa. Next meeting first Tuesday of May, 1874.

*Mad River Dental Association* Meets at Dayton on the first Tuesdays in April and October. *Pres.* S. B. Tizzard, Dayton; *Sec.* A. J. Grosvenor, Troy.

*Maine Dental Society*—Meets in Aug., at Thomaston. *Pres.* E. J. Roberts, Augusta; *Sec.* C. E. Hussey, Biddeford.

*Maryland Association of Dentists* Meets the last Thursday of every month. *Pres.* J. B. Bean; *Sec.* S. M. Field.

*Massachusetts Dental Society*—Meets monthly. *Pres.* J. H. Batchelder, of Salem; *Sec.* C. Wilson; *Cor. Sec.* L. D. Shepard.

*Michigan State Dental Association*—Meets annually second Tuesday of Oct., at Ypsilanti. *Pres.* D. C. Hawxhurst, Ann Arbor; *Rec. & Cor. Sec.* T. R. Perry, Grand Rapids

*Michigan Central Dental Association.* Meets annually at Jackson, Secon Wednesday of January, 1873. *Pres.* C. C. Jenkins, Anu Arbor, *Sec.* W. H. Darrance, Jackson.

*Mississippi Valley Dental Association* Meets annually at Cincinnati, on first Wednesday in March, 1874. *Pres.* S. B. Brown, Ft. Wayne Ind.; *Rec. Sec.* F. Hunter Cin.

*Minnesota Dental Associrtion* Meets annually. Next meeting at Faribault, last Wednesday in June, 1874. *Pres.* J. A. Bowman, Minneapolis; *Sec.* C. D. Montreville, St. Paul.

*Missouri Dental Association* Meets on the first Tuesday of June, in St. Louis. *Pres.* C. W. Rivers, St. Louis; *Sec.* W. H. Evans, St. Louis.

*Missouri Valley Dental Association** Meets on the second Tuesday in July and January. *Pres.* A. S. Billings, Omaha, Neb.; *Sec. and Treas.* J. F. Sanborn, Tabor, Iowa.

*Merrimac Valley Dental Association* Meets semi-annually first Tuesday of Oct. and May.* *Pres.* L. H. Kidder, Mass; *Rec. Sec.* A. M. Dudley, of Lowell; *Cor. Sec.* D. T. Porter, Lawrence, Mass.

*Memphis Dental Society*—Meets on the first Tuesday of each month. *Pres.* W. M. Arrington; *Sec.* V. F. Elliott; *Treas.* C. L. Harris.

*Nashville Dental Association*—Meets on the second Tuesday of each month. *Pres* J. C. Ross; *Sec.* R. R. Freeman; *Cor. Sec.* S. J. Cobb.

*New Jersey State Dental Society*—Meets annually. Next meeting at Mount Holly, on the second Tuesday in July, 1874. *Pres.* J. W. Cosad, Jersey City; *Sec.* J. W. Scarborough, Lambertville.

*New York State Dental Society*--Meets at Albany on the last Wednesday of June next. *Pres.* J. G. Ambler, New Yook; *Sec.* Chas. Barnes, of Syracuse, *Cor. Sec.* S. A. Freeman.

*New Haven Dental Society*—Meets on the first Wednesday of each month at New Haven. *Pres.* J. T. Metcalf, *Rec and Cor. Sec.* C. L. Smith, of New Haven.

*New Orleans Dental Society* Meets monthly. *Pres.* ——————; *Rec. Sec.* A. F McLane.

*Northern Ohio Dental Association.* Meets annually, second Tuesday of June, 1873, at Put-in Bay. *Pres.* J. A. Robinson, Cleveland; *Rec. Sec.* A. F. Price, Fremont.

*Northern Iowa Dental Association*—Meets annually, on the second Tuesday in June, 1873. Next meeting at Waterloo; *Pres.* J. N. Abbott, Lancaster, *Rec. Sec.* A. V. Eaton, Anamosa; *Cor. Sec.* A. R. Mason, Waterloo.

*Ohio College Dental Association*—Meets annually Feb. 25. at Cincinnati. *Pres.* Jas. Leslie, Cin'ti.; *Rec. Sec.* H. A. Smith, of Cincinnati.

*Ohio State Dental Association*--Meets annually at Columbus. Next meeting, first Wednesday of December. *Pres.* H. A. Smith, Cincinnati; *Rec. Sec.* J. M. Porter, Massilon; *Cor. Sec.* C. R. Butler, Cleveland, O.

*Odontographic Society of Pennsylvania*--Meets monthly in Philadelphia. Next meeting, Wednesday, Sept. 1st. *Pres.* C. T. Stellwagen; *Rec. Sec.* A. Boice; *Cor. Sec.* E. L. Hewitt.

*Odontological Society of New York City* Meets the second Tuesday of each monh. *Pres.* C. E. Francis; *Rec. Sec.* W. E. Hong.

*Old Colony Dental Association* Meets quarterly.* *Pres.* C. G. Davis; *Rec. Sec.* L. W. Puffer, North Bridgewater, Mass; *Cor. Sec.* N. C. Fowler, Yarmouth, Mass.

*Ohio Valley Dental Association* Meets annually, in N. E. Ky; this year in Paris, Ky. Tuesday, Sept. 9th.

*Oregon State Dental Society* Meets annually. *Pres.* J. H. Hatch, Portland; *Rec. Sec.* J. R. Cardwell, Portland.

*Pennsylvania State Dental Society* Meets next at Wilkesbarre, second Tuesday of July, 1874. *Pres.* G. W. Klump, Williamsport; *Rec. Sec.* R. H. Moffit, Harrisburg *Cor. Sec.* S. Welchens, Lancaster.

*Poughkeepsie Dental Society* Meets monthly. *Pres.* J. H. Mann; *Sec.* H. F. Clark

*Pennsylvania Association of Dental Surgery* Meets monthly in Philadelphia. *Pres.* Spencer Roberts; *Sec.* E. R. Pettit.

*San Francisco Dental Association* Meets monthly. *Pres.* C. C. Knowles; *Cor. Sec.* H. E. Knox; *Rec. Sec.* Wm. Dutch.

*St. Louis Dental Society* Meets monthly. *Pres.* W. H. Morrison; *Sec.* A. H. Fuller.

*Susquehanna Dental Association* Meets annually.* *Pres.* J. L. Fordham: *Rec. Sec.* G. W. Klump, Williamsport. Meets Nov. 12, 1873.

*Society of Dental Surgeons of the City of New York* Meets semi-monthly in New York. *Pres.* B. F. Franklin: *Rec. Sec.* J. S. Latimer; *Cor. Sec.* E. H. Burras.

**Southern Dental Association* Meets annually. Next meeting the last Tuesday of July, 1873, at Baltimore, Md. *Pres.* H. M. Grant Virginia.: *Rec. Sec.* J. F. Thompson Va

*South Carolina State Dental Association* Meets semi-annually in May and Nov. *Pres.* W. C. Wardlaw, Abbeville: *Sec.* O. J. Bond, Marion.

*Tennessee Dental Association* Meets semi-annually. Next meeting in Nashville, on the 4th Wednesday in June. 1874. *Pres.* R. Russell, Nashville: *Sec.* A. Hartman, Murfreesboro; *Cor. Sec.* L. G. Noel, Nashville.

*Virginia State Dental Association* Meets in Richmond, first Thursday in Nov. of each year. *Pres.* H. M. Grant, Abington: *Sec.* G. F. Kersee, Richmond.

*Washington, City Dental Society* Meets annually. *Pres.* R. F. Hunt, *Sec.* Dr. Evans.

*Wabash Valley Dental Association* Meets annually. Next meeting at Wayne, third Tuesday in March, 1872. *Pres.* A. B. Cunningham, Attica: *Sec.* S. B. Brown, Fort Wayne.

* *Wisconsin State Dental Association* Meets semi-annually. Next meeting at Watertown. second Tuesday of January, 1872. *Pres.* Edgar Palmer, Le Crook *Sec.* C. C. Chittenden, Madison.

**Place of meeting fixed by appointment,*

### District Dental Societies of the State of New York.

*First District Dental Society* Meets Monday in New York. Annual meeting, second Wednesday in June : *Pres.* E. A. Bogue, New York : *Sec.* J. S. Latimer,. N. Y.

*Second District Dental Society.* Meets quarterly in Brooklyn. Annual meeting second Tuesday in April. *Pres.* W. S. Elliott : *Rec. Sec.* M. E. Elmandorf. Brooklyn : *Cor.* F. W. Dalbare.

*Third District Dental Society* Meets at Albany semi-annually and annually. *Pres.* D. D. French, Troy : *Sec.* S. J. Andress, Albany.

**Fourth District Dental Society* Meets annually in June at Saratoga. *Pres.* C, A. Elmore, Saratoga Springs. *Sec.* E. S. Pearsall, Fort Edwards.

**Fifth District Dental Society* Meets annually at Saratoga. *Pres.* W. W. Perkins *Sec.* F. D. Neelans.

**Sixth District Dental Society* Annual meeting at Binghampton, second Tuesday in June. *Pres.* R. Walker : *Rec. Sec.* P. L. Foote

**Seventh District Dental Society* Annual meeting is held the last Tuesday in June, at Rochester. *Pres.* G. W. Tripp, Auburn ; *Sec.* J. E. Line, Rochester.

*Eighth District Dental Society* Meets annually on the second Tuesday in May, at Buffalo : *Bres.* J. C. Gifford, *Sec.* S. A. Freeman, Buffalo.

T*he Seventh and Eighth District Societies* Hold a union meeting on the first Tuesday of October, alternately at Buffalo and Rochester

*Place and time of meeting fixed by appointment.

---

The Ohio State Board of Dental Examiners will meet in Columbus on the first Tuesday of Dec. 1874, at 10 o'clock. *Pres.* J. Taft; *Sec.* W. P. Horton.

---

### List of Exchanges.

ST. LOUIS MEDICAL AND SURGICAL JOURNAL.
BOSTON MEDICAL AND SURGICAL JOURNAL.
THE PACIFIC MEDICAL AND SURGICAL JOURNAL, SAN FRANCISCO.
BUFFALO MEDICAL AND SURGICAL JOURNAL.
PHIDADELPHIA MEDICAL AND SURGICAL JOURNAL.
CINCINNATI LANCET AND OBSERVER.
DENTAL COSMOS, PHILADELPHIA,
BRAITHWAITE'S RETROSPECT, N. Y.
THE DENTAL TIMES, PHILADELPHIA.
LADIES' REPOSITORY, CINCINNATI.
HALL'S JOURNAL OF HEALTH, N. Y.
CHICAGO MEDICAL EXAMINER,
THE DETROIT REVIEW OF MEDICINE AND PHARMACY.
MEDICAL RECORD, N. Y.
NASHVILLE JOURNAL OF MEDICINE AND SURGERY.
AMERICAN ECLECTIC MEDICAL REVIEW. N. Y..
AMERICAN JOURNAL OF DENTAL SCIENCE, BALTIMORE.
THE UNITED STATES MEDICAL AND SURGICAL JOURNAL. CHICAGO
NEW ORLEANS JOURNAL OF MEDICINE,
WESTERN JOURNAL OF MEDICINE, INDIANAPOLIS, IND.
AMERICAN AGRICULTURIST, N. Y.
BOSTON JOURNAL OF CHEMISTRY.
JOURNAL OF APPLIED CHEMISTRY, N. Y.
DRUGGISTS' CIRCULAR AND CHEMICAL GAZETTE, N. Y.
CANADA JOURNAL OF DENTAL SCIENCE, MONTREAL.
INDIANA JOURNAL OF MEDICINE,
JOURNAL OF THE GYNÆCOLOGICAL SOCIETY, BOSTON.
MEDICAL AND SURGICAL REPORTER. SALEM, OREGON.
MISSOURI DENTAL JOURNAL, ST. LOUIS
ECLECTIC MEDICAL JOURNAL, CINCINNATI.

AMERICAN DENTAL ASSOCIATION—Meets on the first Tuesday in Aug., 1875, at Niagara Falls. *Pres.* M. S. Dean. Chicago; *Cor. Sec.* Geo. L, Filed, Detroit; *Rec. Sec.* C. Stodard Smith, Springfield, Ill.

*American Dental Convention* Meets at Saratoga Springs, second Tuesday in Aug., 1874. *Pres.* John Allen, New York City ; *Cor. Sec.* A. Tees, Philadelphia, Pa., *Rec. Sec.* C. L. Hurlbut, Springfield, Mass.

*List of Societies Represented in the American Dental Association.*

*American Academy of Dental Science* Meets at Boston, Mass. *Pres.* Joshua Tucker M. D , of Boston ; *Sec.* W. L. Tucker, of Boston ; *Cor. Sec.*, E. N. Harris.

*Brooklyn Dental Society* Meets semi-monthly. *Pres.* W. T. Shannon, ; *Rec. Sec.* C. P. Crandell ; *Cor. Sec.* A. N. Chapman.

*Buffalo Dental Society* Meets second Monday of each month at Buffalo. *Pres.* S. A. FREEMAN ; *Rec. Sec.* G. C. DABOLL.

*Boston Dental Institute*—Meets first Tuesday of each month. [*Pres.* D. G. WILLIAMS, Buffalo; *Cor. Sec.* D. S. Dickerman.

*California State Dental Association* Meets annually in June. *Pres.* C. C. Knowles, San Francisco ; *Rec. Sec.* H. J. Plomteaux, Woodland.

*Cincinnati Dental Society*—Meets Semi-monthly at Cincinnati. *Pres.* H. R. SMITH ; *Rec. Sec.* D. C. HAWXHURST.

*Chicago Dental Society* Meets monthly at Chicago. *Pres.* M. S. Dean ; *Rec. Sec.* Edgar D. Swain.

*Connecticut Valley Dental Association.* Meets semi-annually, second Tuesday of June and October. *Pres.* H. M. Miller, Westfield, Mass.; *Rec. Sec.* L. C. Taylor, of Holyoke, Mass.

*Connecticut State Dental Association. Pres.* R. C. Dunham, New Britain ; *Rec. Sec.* D. E. Lane, Hartford.

*Charleston Dental Association*—Meets second Tuesday of every month ; *Pres.* T. F. Chupein ; *Sec. and Treas.* B. A. Muckenfuss.

*Delaware Dental Association*- -Meets semi-annually. *Pres.* E. W. Haines, Newark ; *Sec.* C. R. Jeffries, Wilmington.

*Eastern Indiana Dental Association.*—Meets semi-annually, first Tuesday in May and last Tuesday in October; *Pres.* W. C. Stanley, Dublin, Ind.; *Sec.* J. W. White, Knightstown, Ind. (Place of meeting fixed by appointment.)

*Eastern Ohio Dental Association*- Meets on Tuesday, August 26th, 1873, at Alliance ; *Pres.* A. J. Douds ; *Sec.* J. M. Porter.

*East Tennessee Dental Association*—Meets annually at Knoxville, Tenn. *Pres.* Wm. W. F. Fowler; *Rec. Sec.* S. M. Prothro.

*Forest City Society of Dental Surgeons*- *Pres.* B. Strickland, Cleveland, *Rec. Sec.* H. L. Ambler, Cleveland ; *Cor. Sec.* Chas. Buffett, Cleveland.

*Georgia State Dental Society* Next meeting at Atlanta, July 1873. *Pres.* F. Y. Clark ; *Cor. Sec.* J. P. H. Brown, Augusta.

*Harris Dental Association* Meets quarterly on the first Thursday in February, May. August, November. *Pres.* J. A. Martin, Strasburg ; *Sec.* Wm. N. Amer, Lancaster,

*Hudson River Association of Dental Surgeons* Meets on the second Wednesdays of May, September and January. *Pres.* L. S. Straw, Newburg, N. Y ; *Rec. Sec.* T. W. DuBois, Poughkeepsie, N. Y.

*Hudson Valley Dental Association* Meets monthly at Troy, N. Y. *Pres.* L. C. Wheeler, *Rec. Sec.* S. G. Andres ; *Cor. Sec.* S. D. French.

*Illinois State Dental Society* Meets annually. *Pres.* G. S. Miles, Jerseyville, *Sec.* C. R. E. Koch, Chicago. Meets second Tuesday in May, 1875, at Ottowa.

*Indiana State Dental Society*—Meets next year at Indianapolis, on the first Tuesday of June. *Pres.* M. Wells, Indianapolis ; *Cor. and Rec. Sec.* P. McDonald, Goshen.

*Iowa State Dental Society*- -Meets annually. Next meeting at Davenport, on the second Tuesday of May. *Pres.* L. C. Ingersoll, Keokuk. *Rec. Sec.* E. P. White, of Davenport ; *Cor. Sec.* A. J. Redrich, Sioux City.

*Kansas State Dental Society* Meets annually. Next meeting at Leavanworth, first Wednesday in May, 1875. *Pres.* L. C. Wasson; *Sec.* J. D Patterson.

*Kansas and Missouri Valley Dental Association*—Meets first and third Tuesdays of erch month. *Pres.* J. R.Stark ; *Sec.* H. C. Massie, Kansas City, Mo.

*Kentucky State Dental Association* Meets annually and semi-monthly, in the City of Louisville. Annual meeting, first Tuesday in June, at 2 o'clock, P. M. Semi-monthly meetings, second and fourth Tuesday nights. G. W. Priest, *Pres.* ; B. O. Doyle. *Sec.*, both at Louisville.

*Lake Erie Dental Association* Meets Annually. *Pres.* C. B. Ansart, Oil City, Pa. *Sec.* C. D. Elliot, Franklin, Pa. Next meeting first Tuesday of May, 1874.

*Mad River Dental Association* Meets at Dayton on the first Tuesdays in April and October. *Pres.* S. B. Tizzard, Dayton ; *Sec.* A. J. Grosvenor, Troy.

*Maine Dental Society*—Meets in Aug., at Thomaston. *Pres.* E. J. Roberts, Augusta ; *Sec.* C. E. Hussey, Biddeford.

*Maryland Association of Dentists* Meets the last Thursday of every month. *Pres.* J. B. Bean ; *Sec.* S. M. Field.

*Massachusetts Dental Society*—Meets monthly. *Pres.* J. H. Batchelder, of Salem ; *Sec.* C. Wilson ; *Cor. Sec.* L. D. Shepard.

*Michigan State Dental Association*—Meets annually second Tuesday of Oct., at Ypsilanti. *Pres.* D. C. Hawxhurst, Ann Arbor; *Rec. & Cor. Sec.* T. R. Perry, Grand Rapids

*Michigan Central Dental Association.* Meets annually at Jackson, Secon Wednesday of January, 1873. *Pres.* C. C. Jenkins, Anu Arbor, *Sec.* W. H. Darrance, Jackson.

*Mississippi Valley Dental Association* Meets annually at Cincinnati, on first Wednesday in March, 1874. *Pres.* S. B. Brown, Ft. Wayne Ind.; *Rec. Sec.* F. Hunter Cin.
*Minnesota Dental Associrtion* Meets annually. Next meeting at Faribault, last Wednesday in June, 1874. *Pres.* J. A. Bowman, Minneapolis; *Sec.* C. D. Montreville, St. Paul.
*Missouri Dental Association* Meets on the first Tuesday of June, in St. Louis. *Pres.* C. W. Rivers, St. Louis; *Sec.* W. H. Evans, St. Louis.
*Missouri Valley Dental Association** Meets on the second Tuesday in July and January. *Pres.* A. S. Billings, Omaha, Neb.; *Sec. and Treas.* J. F. Sanborn, Tabor, Iowa.
*Merrimac Valley Dental Association* Meets semi-annually first Tuesday of Oct. and May.* *Pres.* L. H. Kidder, Mass.; *Rec. Sec.* A. M. Dudley, of Lowell; *Cor. Sec.* D. T. Porter, Lawrence, Mass.
*Memphis Dental Society*—Meets on the first Tuesday of each month. *Pres.* W. M. Arrington; *Sec.* V. F. Elliott; *Treas.* C. L. Harris.

*Nashville Dental* [illegible]—Meets on the second Tuesday of each month. *Pres* J. C. Ross; *Sec.* R. R. Freeman; *Cor. Sec.* S. J. Cobb.
*New Jersey State Dental Society*—Meets annually. Next meeting at Mount Holly, on the second Tuesday in July, 1874. *Pres.* J. W. Cosad, Jersey City; *Sec.* J. W. Scarborough, Lambertville.
*New York State Dental Society*--Meets at Albany on the last Wednesday of June next. *Pres.* W. C. Barret, Warsaw, N. Y.; *Sec.* Chas. Barnes, of Syracuse, *Cor. Sec.* W. S. Elliott, Goshen; *Rec. Sec.* Charles Barnes, Syracuse,
*New Haven Dental Society*--Meets on the first Wednesday of each month at New Haven. *Pres.* J. T. Metcalf, *Rec and Cor. Sec.* C. L. Smith, of New Haven.
*New Orleans Dental Society* Meets monthly. *Pres.* ——————; *Rec. Sec.* A. F McLane.
*Northern Ohio Dental Association.* Meets annually, second Tuesday of June, 1873, at Put-in Bay. *Pres.* J. A. Robinson, Cleveland; *Rec. Sec.* A. F. Price, Fremont.
*Northern Iowa Dental Association*—Meets annually, on the second Tuesday in June, 1873. Next meeting at Waterloo; *Pres.* J. N. Abbott, Lancaster, *Rec. Sec.* A. V. Eaton, Anamosa; *Cor. Sec.* A. R. Mason, Waterloo.

*Ohio College Dental Association*—Meets annually Feb. 25. at Cincinnati. *Pres.* Jas. Leslie, Cin'ti.; *Rec. Sec.* H. A. Smith, of Cincinnati.
*Ohio State Dental Association*—Meets annually at Columbus. Next meeting, first Wednesday of December. *Pres.* H. A. Smith, Cincinnati; *Rec. Sec.* J. M. Porter, Massilon; *Cor. Sec.* C. R. Butler, Cleveland, O.
*Odontographic Society of Pennsylvania*—Meets monthly in Philadelphia. Next meeting, Wednesday, Sept. 1st. *Pres.* C. T. Stellwagen; *Rec. Sec.* E. L. Hewitt.
*Odontological Society of New York City* Meets the second Tuesday of each monh. *Pres.* C. E. Francis; *Rec. Sec.* W. E. Hong.
*Old Colony Dental Association* Meets quarterly.* *Pres.* C. G. Davis; *Rec. Sec.* L. W. Puffer, North Bridgewater, Mass; *Cor. Sec.* N. C. Fowler, Yarmouth, Mass.
*Ohio Valley Dental Association* Meets annually, in N. E. Ky; this year in Paris, Ky. Tuesday, Sept. 9th.
*Oregon State Dental Society* Meets annually. *Pres.* J. H. Hatch, Portland; *Rec. Sec.* J. R. Cardwell, Portland.

*Pennsylvania State Dental Society* Meets next at Wilkesbarre, second Tuesday of July, 1874. *Pres.* G. W. Klump, Williamsport; *Rec. Sec.* R. H. Moffit, Harrisburg *Cor. Sec.* S. Welchens, Lancaster.
*Poughkeepsie Dental Society* Meets monthly. *Pres.* J H. Mann; *Sec.* H. F. Clark
*Pennsylvania Association of Dental Surgery* Meets monthly in Philadelphia. *Pres.* Spencer Roberts; *Sec.* E. R. Pettit.

*San Francisco Dental Association* Meets monthly. *Pres.* C. C. Knowles; *Cor. Sec.* H. E. Knox; *Rec. Sec.* Wm. Dutch.
*St. Louis Dental Society* Meets monthly. *Pres.* W. H. Morrison; *Sec.* A. H. Fuller.
*Susquehanna Dental Association* Meets annually.* *Pres.* J. L. Fordham: *Rec. Sec.* Wm. Nertz. Meets Nov. 11, 1874, at Lewisburg.
*Society of Dental Surgeons of the City of New York* Meets semi-monthly in New York. *Pres.* B. F. Franklin: *Rec. Sec.* J. S. Latimer; *Cor. Sec.* E. H. Burras.
**Southern Dental Association* Meets annually. Next meeting the last Tuesday of July, 1873, at Baltimore, Md. *Pres.* H. M. Grant Virginia.: *Rec. Sec.* J. F Thompson Va
*South Carolina State Dental Association* Meets semi-annually in May and Nov. *Pres.* T. F. Chupein, Charleston; *Sec.* J. W. Norwood, Greenville; *Cor. Sec.* C. C. Patrick, Charleston, S. C. Next meeting at Columbia.
*Tennessee Dental Association* Meets semi-annually. Next meeting in Nashville, on the 4th Wednesday in June. 1875. *Pres.* E. S. Chisholm; *Sec.* J. S. King; *Cor. Sec.* S. J. Cobb.
*Virginia State Dental Association* Meets in Richmond, first Thursday in Nov. of each year. *Pres.* H. M. Grant, Abington: *Sec.* G. F. Kersee, Richmond.

*Washington, City Dental Society* Meets annually. *Pres.* R. F. Hunt, *Sec.* Dr. Evans.
*Wabash Valley Dental Association* Meets annually. Next meeting at Wayne, third Tuesday in March, 1872. *Pres.* A. B. Cunningham, Attica: *Sec.* S. B. Brown, Fort Wayne.
* *Wisconsin State Dental Association* Meets semi-annually. Next meeting at Watertown. second Tuesday of January, 1872. *Pres.* Edgar Palmer, Le Crook *Sec.* C. C. Chittenden, Madison.

**Place of meeting fixed by appointment*

### District Dental Societies of the State of New York.

*First District* Meets second and fourth Wednesday evenings of each month. Annual meeting in New York, first Tuesday in June. *Pres.* R. M. Gage; *Rec. Sec.* W. E. Hoag, 331 Madison ave., New York.
*Second District.* Annual meeting in Brooklyn, first Monday in April. *Pres.* W. S. Elliott; *Rec. Sec.* M. E. Elmandorf. Brooklyn; *Cor. Sec.* F. W. Dalbare.
*Third District.* Meets semi-annually on second Tuesday in January and July. Annual meeting at Albany on second Tuesday in January. *Pres.* J. C. Austin. Albany; *Rec. Sec.* S. J. Andress, Troy; *Cor. Sec.* D. H. Young, Troy.
*Fourth District.* Meets semi-annually. Meets annually at Saratoga on second Tuesday in June. *Pres.* C, A. Elmore, Ft. Edward; *Rec. Sec.* E. S. Pearsall, Saratoga.
*Fifth District.* Meets semi-annually. Annual meeting at Syracuse. second Tuesday in June. *Pres.* J. D. Huntington, Watertown; *Rec. Sec.* F. D. Nellis, Syracuse; *Cor. Sec.* J. S. Marshall, Syracuse.
*Sixth District.* Meets semi-annually. Annual meeting at Binghampton on second Tuesday in June. *Pres.* Frank B. Darby, Oswego; *Rec. Sec.* F. E. Perry, Norwich; *Cor. Sec.* A. M. Holmes, Morrisville.
*Seventh District.* Meets semi-annually. Annual meeting at Rochester first Tuesday in June. *Pres.* H. S. Miller; *Rec. Sec.* J. E. Line, Rochester.
*Eighth District.* Meets semi-annually. Annual on second Tuesday in May. *Pres* G. C. Daboll, *Rec. Sec.* S. A. Freeman, *Cor. Sec.* T. G. Lewis, Buffalo.
*Buffalo Dental Association.* Meets on second Tuesday evening of each month. Annual meeting in June. *Pres.* S. A. Freeman; *Sec.* W. H. Barrows.

The Ohio State Board of Dental Examiners will meet in Columbus on the first Tuesday of Dec. 1874, at 10 o'clock. *Pres.* J. Taft; *Sec.* W. P. Horton.

### List of Exchanges.

ST. LOUIS MEDICAL AND SURGICAL JOURNAL.
BOSTON MEDICAL AND SURGICAL JOURNAL.
THE PACIFIC MEDICAL AND SURGICAL JOURNAL, SAN FRANCISCO.
PHIDADELPHIA MEDICAL AND SURGICAL JOURNAL.
CINCINNATI LANCET AND OBSERVER.
DENTAL COSMOS, PHILADELPHIA.
LADIES' REPOSITORY, CINCINNATI.
HALL'S JOURNAL OF HEALTH, N. Y.
CHICAGO MEDICAL EXAMINER.
THE DETROIT REVIEW OF MEDICINE AND PHARMACY.
MEDICAL RECORD, N. Y.
NASHVILLE JOURNAL OF MEDICINE AND SURGERY.
AMERICAN JOURNAL OF DENTAL SCIENCE, BALTIMORE.
THE UNITED STATES MEDICAL AND SURGICAL JOURNAL, CHICAGO
NEW ORLEANS JOURNAL OF MEDICINE.
AMERICAN AGRICULTURIST, N. Y.
BOSTON JOURNAL OF CHEMISTRY.
JOURNAL OF APPLIED CHEMISTRY, N. Y.
CANADA JOURNAL OF DENTAL SCIENCE, MONTREAL.
INDIANA JOURNAL OF MEDICINE.
MEDICAL AND SURGICAL REPORTER. SALEM, OREGON
MISSOURI DENTAL JOURNAL, ST. LOUIS
ECLECTIC MEDICAL JOURNAL, CINCINNATI.

### OHIO COLLEGE OF DENTAL SURGERY.

The Twenty-ninth Annual Session of the Ohio College of Dental Surgery will commence on the 15th of October next, and continue until March following.

### FACULTY.

JAMES TAYLOR, M. D., D. D. S.
Emeritus, Professor of Institutes of Dental Science.
WM. CLENDENIN, M. D.
Professor of Anatomy.
J. S. CASSIDY, D. D. S.
Professor of Chemistry and Therapeutics.
J. L. CILLEY, M. D.
Professor of Physiology and Histology.
E. BRUNNING, M. D.
Professor of Pathology and Therapeutics.
J. TAFT, D. D. S.
Professor of Operative Dentistry and Dental Hygiene.
Wm. VAN ANTWERP, D. D. S.
Professor of Mechanical Dentistry.
JAMES I. TAYLOR, JR., D. D. S.
Professor of Clinical Dentistry.

### TEXT BOOKS.

Gray's and Wilson's Anatomy, Dalton's or Carpenter's Phisiology; William's Pathology, Fowne's, Turner's, Graham's, or Brandt and Taylor's Chemistry; Taft's Operative Dentistry; Richardson's Mechanical Dentistry; Harris' Principles and Practice of Dental Science; Tomes' Dental Surgery; Watt's Chemical Essays; Harris' Dental Dictionary.

### TERMS OF ADMISSION.

Tickets must be procured at the beginning of the session.

| | |
|---|---|
| Tickets for the Course - - - - - - | $100.00 |
| Demonstrator's Ticket for Anatomy - - - | 5.00 |
| Matriculation Fee, but once - - - - | 5.00 |
| Diploma Fee - - - - - - - - | 30.00 |

### TERMS OF GRADUATION.

Two courses (including one of the dissections,) with a satisfactory dental pupilage. Eight years reputable practice, or a satisfactory examination upon the branches of the Junior Course in this Institution, will be received as equal to one course.

An acceptable Thesis upon some subject in Dental Science.

Must possess a good moral character, and be twenty-one years old.

For further information, address J. TAFT, DEAN,
117 W. Fourth Street, Cincinnati, O.

### PENNSYLVANIA COLLEGE OF DENTAL SURGERY,

**S. E. COR. ARCH & TENTH STS., PHILADELPHIA.**

THE NINETEENTH ANNUAL SESSION, 1874-'75.

### FACULTY:

T. L. BUCKINGHAM, D. D. S.,
*Professor of Chemistry.*
E. WILDMAN, M. D., D. D. S..
*Professor of Mechanical Dentistry and Metallurgy.*
G. T. BAKER, D. D. S.,
*Professor of Dental Pathology and Therapeutics.*
JAMES TRUMAN, D. D. S.,
*Professor of Dental Histology and Operative Dentistry.*
JAMES TYSON, M. D.,
*Professor of Physiology and Microscopic Anatomy.*
J. EWING MEARS. M. D.,
*Professor of Anatomy and Surgery.*
E. R. PETTITT, D. D. S.,
*Demonstrator of Operative Dentistry.*
A. B. ABELL, JR., D. D. S.,
*Demonstrator of Mechanical Dentistry.*
J. L. SAXTON, D. D. S.,
*Assistant Demonstrator of Operative Dentistry.*
A. H HENDERSON, D. D. S.,
*Assistant Demonstrator of Mechanical Dentistry.*

### CLINICAL INSTRUCTORS:

A. LAWRENCE, M D., D. D. S.,

A. L. NORTHRUP, D. D. S.,

C. A. MARION, D. D. S.,

C. PALMER, D. D. S.,

E. T. DARBY, D. D. S.,

R. HUSY, D. D. S.,

W. H. TRUEMAN, D. D. S.

The College will be open for instruction during the entire year, except the last weeks in August and the first week in September. In the Dispensary and Laboratory, ample opportunities will be afforded the student for the prosecution of the practical parts of the profession, under the guidance and supervision of Demonstrators of known integrity and capacity. A Clinical Lecture will be given and operations performed by one of the Professors every Saturday afternoon during the sessions.

### *SPRING AND FALL SESSIONS.*

The Spring Course of Lectures will commence on the first Monday in April, and continue until the last of June. Fee for the Course $50 00.

The Fall Course will commence on the second Monday in September, and continue until the first of October, and will be free to those who matriculate for the Regular Session.

During the Spring and Fall Sessions there will be one Lecture a day, from 5 to 6 o'clock P. M.. and with the exception of Saturday, the Dispensary and Laboratory will be open daily for six hours.

The attendance of the entire Spring and Fall Courses will be deemed equivalent to the term of pupilage under a private preceptor during the recess of the regular sessions.

### *THE REGULAR SESSION*

Will commence on the first Monday of November, and continue until the first of March ensuing, The course is so arranged that eighteen lectures will be delivered each week on the various branches taught in the College.

FEES.

| | |
|---|---|
| Matriculation, (paid but once) | $ 5 00 |
| For the Course, (Demonstrator's Ticket included) | 100 00 |
| Diploma, | 30 00 |

For further information, address,

**E. WILDMAN, Dean,**

**No. 1205 Arch Street, Philadelphia.**

Board can be obtained at from $5 00 to $8 00 per week.

All the Instruments and Tools required can be obtained for from $20 00 to $25 00.

jjason.

American Dental Association—Meets on the first Tuesday in Aug., 1875, at Niagara Falls. *Pres.* M. S. Dean, Chicago; *Cor. Sec.* Geo. L, Filed, Detroit; *Rec. Sec.* C. Stodard Smith, Springfield, Ill.

*American Dental Convention* Meets at Saratoga Springs, second Tuesday in Aug., 1874. *Pres.* John Allen, New York City; *Cor. Sec.* A. Tees, Philadelphia, Pa., *Rec. Sec.* C. L. Hurlbut, Springfield, Mass.

*List of Societies Represented in the American Dental Association.*

*American Academy of Dental Science* Meets at Boston, Mass. *Pres.* Joshua Tucker M. D., of Boston; *Sec.* W. L. Tucker, of Boston; *Cor. Sec.*, E. N. Harris.

*Brooklyn Dental Society* Meets semi-monthly. *Pres.* W. T. Shannon,; *Rec. Sec.* C. P. Crandell; *Cor. Sec.* A. N. Chapman.

*Buffalo Dental Society* Meets second Monday of each month at Buffalo. *Pres.* S. A. Freeman; *Rec. Sec.* G. C. Daboll.

*Boston Dental Institute*—Meets first Tuesday of each month. *Pres.* D. G. Williams, Buffalo; *Cor. Sec.* D. S. Dickerman.

*California State Dental Association* Meets annually in June. *Pres.* C. C. Knowles, San Francisco; *Rec. Sec.* H. J. Plomteaux, Woodland.

*Cincinnati Dental Society*—Meets Semi-monthly at Cincinnati. *Pres.* H. R. Smith; *Rec. Sec.* D. C. Hawxhurst.

*Chicago Dental Society* Meets monthly at Chicago. *Pres.* M. S. Dean; *Rec. Sec.* Edgar D. Swain.

*Connecticut Valley Dental Association.* Meets semi-annually, second Tuesday of June and October. *Pres.* H. M. Miller, Westfield, Mass.; *Rec. Sec.* L. C. Taylor, of Holyoke, Mass.

*Connecticut State Dental Association.* *Pres.* R. C. Dunham, New Britain; *Rec. Sec.* D. E. Lane, Hartford.

*Charleston Dental Association*—Meets second Tuesday of every month; *Pres.* T. F. Chupein; *Sec. and Treas.* B. A. Muckenfuss.

*Delaware Dental Association*—Meets semi-annually. *Pres.* E. W. Haines, Newark; *Sec.* C. R. Jeffries, Wilmington.

*Eastern Indiana Dental Association.*—Meets semi-annually, first Tuesday in May and last Tuesday in October; *Pres.* W. C. Stanley, Dublin, Ind.; *Sec.* J. W. White, Knightstown, Ind. (Place of meeting fixed by appointment.)

*Eastern Ohio Dental Association*—Meets on Tuesday, August 26th, 1873, at Alliance; *Pres.* A. J. Douds; *Sec.* J. M. Porter.

*East Tennessee Dental Association*—Meets annually at Knoxville, Tenn. *Pres.* Wm. W. F. Fowler; *Rec. Sec.* S. M. Prothro.

*Forest City Society of Dental Surgeons*—*Pres.* B. Strickland, Cleveland, *Rec. Sec.* H. L. Ambler, Cleveland; *Cor. Sec.* Chas. Buffett, Cleveland.

*Georgia State Dental Society* Next meeting at Atlanta, July 1873. *Pres.* F. Y. Clark; *Cor. Sec.* J. P. H. Brown, Augusta.

*Harris Dental Association* Meets quarterly on the first Thursday in February, May. August, November. *Pres.* J. A. Martin, Strasburg; *Sec.* Wm. N. Amer, Lancaster.

*Hudson River Association of Dental Surgeons* Meets on the second Wednesdays of May, September and January. *Pres.* L. S. Straw, Newburg, N. Y; *Rec. Sec.* T. W. DuBois, Poughkeepsie, N. Y.

*Hudson Valley Dental Association* Meets monthly at Troy, N. Y. *Pres.* L. C. Wheeler, *Rec. Sec.* S. G. Andres; *Cor. Sec.* S. D. French.

*Illinois State Dental Soeiety* Meets annually. *Pres.* G. S. Miles, Jerseyville, *Sec.* C. R. E. Koch, Chicago. Meets second Tuesday in May, 1875, at Ottowa.

*Indiana State Dental Society*—Meets next year at Indianapolis, on the first Tuesday of June. *Pres.* M. Wells, Indianapolis; *Cor. and Rec. Sec.* P. McDonald, Goshen.

*Iowa State Dental Society*—Meets annually. Next meeting at Davenport, on the second Tuesday of May. *Pres.* L. C. Ingersoll, Keokuk. *Rec. Sec.* E. P. White, of Davenport; *Cor. Sec.* A. J. Redrich, Sioux City.

*Kansas State Dental Society* Meets annually. Next meeting at Leavanworth, first Wednesday in May, 1875. *Pres.* L. C. Wasson; *Sec.* J. D Patterson.

*Kansas and Missouri Valley Dental Association*—Meets first and third Tuesdays of erch month. *Pres.* J. R. Stark; *Sec.* H. C. Massie, Kansas City, Mo.

*Kentucky State Dental Association* Meets annually first Tuesday in June, next meeting in Lexington. *Pres.*, B. O. Doyle; *Sec.*, Chas. E Dunn; both of Louisville.

*Lake Erie Dental Association* Meets Annually. *Pres.* C. B. Ansart, Oil City, Pa. *Sec.* C. D. Elliot, Franklin, Pa. Next meeting first Tuesday of May, 1874.

*Mad River Dental Association* Meets at Dayton on the first Tuesdays in April and October. *Pres.* S. B. Tizzard, Dayton; *Sec.* A. J. Grosvenor, Troy.

*Maine Dental Society*—Meets in Aug., at Thomaston. *Pres.* E. J. Roberts, Augusta; *Sec.* C. E. Hussey, Biddeford.

*Maryland Association of Dentists* Meets the last Thursday of every month. *Pres.* J. B. Bean; *Sec.* S. M. Field.

*Massachusetts Dental Society*—Meets monthly. *Pres.* J. H. Batchelder, of Salem; *Sec.* C. Wilson; *Cor. Sec.* L. D. Shepard.

*Michigan State Dental Association*—Meets annually second Tuesday of Oct., at Ypsilanti. *Pres.* D. C. Hawxhurst, Ann Arbor; *Rec. & Cor. Sec.* T. R. Perry, Grand Rapids

*Michigan Central Dental Association.* Meets annually at Jackson, Secon Wednesday of January, 1873. *Pres.* C. C. Jenkins, Anu Arbor, *Sec.* W. H. Darrance, Jackson.

*Mississippi Valley Dental Association* Meets annually at Cincinnati, on first Wednesday in March, 1874. *Pres.* S. B. Brown, Ft. Wayne Ind.; *Rec. Sec.* F. Hunter Cin.

*Minnesota Dental Associrtion* Meets annually. Next meeting at Faribault, last Wed nesday in June, 1874. *Pres.* J. A. Bowman, Minneapolis; *Sec.* C. D. Montreville, St. Paul.

*Missouri Dental Association* Meets on the first Tuesday of June, in St. Louis. *Pres.* C. W. Rivers, St. Louis; *Sec.* W. H. Evans, St. Louis.

*Missouri Valley Dental Association** Meets on the second Tuesday in July and January. *Pres.* A. S. Billings, Omaha, Neb.; *Sec. and Treas.* J. F. Sanborn, Tabor, Iowa.

*Merrimac Valley Dental Association* Meets semi-annually first Tuesday of Oct. and May.* *Pres.* L. H. Kidder, Mass; *Rec. Sec.* A. M. Dudley, of Lowell; *Cor. Sec.* D. T. Porter, Lawrence, Mass.

*Memphis Dental Society*—Meets on the first Tuesday of each month. *Pres.* W. M. Arrington; *Sec.* V. F. Elliott; *Treas.* C. L. Harris.

*Nashville Dental ssociation*--Meets on the second Tuesday of each month. *Pres.* J. C. Ross; *Sec.* R. R. Freeman; *Cor. Sec.* S. J. Cobb.

*New Jersey State Dental Society*—Meets annually. Next meeting at Long Branch, on the first Tuesday in July, 1875. *Pres.* Geo. C. Brown, Mount Holly; *Sec.* J. W. Scarborough, Lambertville.

*New York State Dental Society*--Meets at Albany on the last Wednesday of June next. *Pres.* W. C. Barret, Warsaw, N. Y.; *Sec.* Chas. Barnes, of Syracuse, *Cor. Sec.* W. S. Elliott, Goshen; *Rec. Sec.* Charles Barnes, Syracuse,

*New Haven Dental Society*--Meets on the first Wednesday of each month at New Haven. *Pres.* J. T. Metcalf, *Rec and Cor. Sec.* C. L. Smith, of New Haven.

*New Orleans Dental Society* Meets monthly. *Pres.* ————————; *Rec. Sec.* A. F McLane.

*Northern Ohio Dental Association.* Meets annually, second Tuesday of June, 1873, at Put-in Bay. *Pres.* J. A. Robinson, Cleveland; *Rec. Sec.* A. F. Price, Fremont.

*Northern Iowa Dental Association*—Meets annually, on the second Tuesday in June, 1873. Next meeting at Waterloo; *Pres.* J. N. Abbott, Lancaster, *Rec. Sec.* A. V. Eaton, Anamosa; *Cor. Sec.* A. R. Mason, Waterloo.

*Ohio College Dental Association*--Meets annually Feb. 25. at Cincinnati. *Pres.* Jas. Leslie, Cin'ti.; *Rec. Sec.* H. A. Smith, of Cincinnati.

*Ohio State Dental Association*--Meets annually at Columbus. Next meeting, first Wednesday of December. *Pres.* H. A. Smith, Cincinnati; *Rec. Sec.* J. M. Porter, Massilon; *Cor. Sec.* C. R. Butler, Cleveland, O.

*Odontographic Society of Pennsylvania*—Meets monthly in Philadelphia. Next meeting, Wednesday, Sept. 1st. *Pres.* C. T. Stellwagen; *Rec. Sec.* E. L. Hewitt.

*Odontological Society of New York City* Meets the second Tuesday of each monh. *Pres.* C. E. Francis; *Rec. Sec.* W. E. Hong.

*Old Colony Dental Association* Meets quarterly.* *Pres.* C. G. Davis; *Rec. Sec.* L. W. Puffer, North Bridgewater, Mass; *Cor. Sec.* N. C. Fowler, Yarmouth, Mass.

*Ohio Valley Dental Association* Meets annually, in N. E. Ky; this year in Paris, Ky. Tuesday, Sept. 9th.

*Oregon State Dental Socvety* Meets annually. *Pres.* J. H. Hatch, Portland; *Rec. Sec.* J. R. Cardwell, Portland.

*Pennsylvania State Dental Society* Meets next at Wilkesbarre, second Tuesday of July, 1874. *Pres.* G. W. Klump, Williamsport; *Rec. Sec.* R. H. Moffit, Harrisburg *Cor. Sec.* S. Welchens, Lancaster.

*Poughkeepsie Dental Society* Meets monthly. *Pres.* J. H. Mann; *Sec.* H. F. Clark

*Pennsylvania Association of Dental Surgery* Meets monthly in Philadelphia. *Pres.* Spencer Roberts; *Sec.* E. R. Pettit.

*San Francisco Dental Association* Meets monthly. *Pres.* C. C. Knowles; *Cor. Sec.* H. E. Knox; *Rec. Sec.* Wm. Dutch.

*St. Louis Dental Society* Meets monthly. *Pres.* W. H. Morrison; *Sec.* A. H. Fuller.

*Susquehanna Dental Association* Meets annually.* *Pres.* J. L. Fordham: *Rec. Sec.* Wm. Nertz. Meets Nov. 11, 1874, at Lewisburg.

*Society of Dental Surgeons of the City of New York* Meets semi-monthly in New York. *Pres.* B. F. Franklin: *Rec. Sec.* J. S. Latimer; *Cor. Sec.* E. H. Burras.

**Southern Dental Association* Meets annually. Next meeting the last Tuesday of July, 1873, at Baltimore, Md. *Pres.* H. M. Grant Virginia.: *Rec, Sec.* J. F Thompson Va

*South Carolina State Dental Association* Meets semi-annually in May and Nov. *Pres.* T. F. Chupein, Charleston; *Sec.* J. W. Norwood, Greenville; *Cor. Sec.* C. C. Patrick, Charleston, S. C. Next meeting at Columbia.

*Tennessee Dental Association* Meets semi-annually. Next meeting in Nashville, on the 4th Wednesday in June. 1875. *Pres.* E. S. Chisholm; *Sec.* J. S. King; *Cor. Sec.* S. J. Cobb.

*Virginia State Dental Association* Meets in Richmond, first Thursday in Nov. of each year. *Pres.* H. M. Grant, Abington: *Sec.* G. F. Kersee, Richmond,

*Washington, City Dental Society* Meets annually. *Pres.* R. F. Hunt, *Sec.* Dr. Evans.

*Wabash Valley Dental Association* Meets annually. Next meeting at Wayne, third Tuesday in March, 1872. *Pres.* A. B. Cunningham, Attica: *Sec,* S. B. Brown, Fort Wayne.

**Wisconsin State Dental Association* Meets semi-annually. Next meeting at Watertown. second Tuesday of January, 1872, *Pres.* Edgar Palmer, Le Crook *Sec.* C. C. Chittenden, Madison.

**Place of meeting fixed by appointment*

### District Dental Societies of the State of New York.

*First District* Meets second and fourth Wednesday evenings of each month. Annual meeting in New York, first Tuesday in June. *Pres.* R. M, Gage; *Rec. Sec.* W. E. Hoag, 331 Madison ave., New York.

*Second District.* Annual meeting in Brooklyn, first Monday in April. *Pres.* W. S. Elliott; *Rec. Sec.* M. E. Elmandorf. Brooklyn; *Cor. Sec.* F. W. Dalbare,

*Third District.* Meets semi-annually on second Tuesday in January and July. Annual meeting at Albany on second Tuesday in January. *Pres.* J. C. Austin, Albany; *Rec. Sec.* S. J. Andress, Troy; *Cor. Sec.* D. H. Young, Troy.

*Fourth District.* Meets semi-annually. Meets annually at Saratoga on second Tuesday in June. *Pres.* C, A. Elmore, Ft. Edward; *Rec. Sec.* E. S. Pearsall, Saratoga.

*Fifth District.* Meets semi-annually. Annual meeting at Syracuse. second Tuesday in June. *Pres.* J. D. Huntington, Watertown; *Rec. Sec.* F. D. Nellis, Syracuse; *Cor. Sec.* J. S. Marshall, Syracuse.

*Sixth District.* Meets semi-annually. Annual meeting at Binghampton on second Tuesday in June. *Pres.* Frank B. Darby, Oswego; *Rec. Sec.* F. E. Perry, Norwich; *Cor. Sec.* A. M. Holmes, Morrisville.

*Seventh District.* Meets semi-annually. Annual meeting at Rochester first Tuesday in June. *Pres.* H. S. Miller; *Rec. Sec.* J. E. Line, Rochester.

*Eighth District.* Meets semi-annually. Annual on second Tuesday in May. *Pres* G. C. Daboll, *Rec. Sec.* S. A. Freeman, *Cor. Sec.* T. G. Lewis, Buffalo.

*Buffalo Dental Association.* Meets on second Tuesday evening of each month. Annual meeting in June. *Pres.* S. A, Freeman; *Sec.* W. H. Barrows.

The Ohio State Board of Dental Examiners will meet in Columbus on the first Tuesday of Dec. 1874, at 10 o'clock. *Pres.* J. Taft; *Sec.* W. P. Horton,

### List of Exchanges.

ST. LOUIS MEDICAL AND SURGICAL JOURNAL.
BOSTON MEDICAL AND SURGICAL JOURNAL.
THE PACIFIC MEDICAL AND SURGICAL JOURNAL, SAN FRANCISCO.
PHIDADELPHIA MEDICAL AND SURGICAL JOURNAL.
CINCINNATI LANCET AND OBSERVER.
DENTAL COSMOS, PHILADELPHIA,
LADIES' REPOSITORY, CINCINNATI,
HALL'S JOURNAL OF HEALTH, N. Y.
CHICAGO MEDICAL EXAMINER,
THE DETROIT REVIEW OF MEDICINE AND PHARMACY.
MEDICAL RECORD, N. Y.
NASHVILLE JOURNAL OF MEDICINE AND SURGERY.
AMERICAN JOURNAL OF DENTAL SCIENCE, BALTIMORE.
THE UNITED STATES MEDICAL AND SURGICAL JOURNAL, CHICAGO
NEW ORLEANS JOURNAL OF MEDICINE,
AMERICAN AGRICULTURIST, N. Y.
BOSTON JOURNAL OF CHEMISTRY.
JOURNAL OF APPLIED CHEMISTRY, N. Y.
CANADA JOURNAL OF DENTAL SCIENCE, MONTREAL.
INDIANA JOURNAL OF MEDICINE,
MEDICAL AND SURGICAL REPORTER, SALEM, OREGON
MISSOURI DENTAL JOURNAL, ST. LOUIS.
ECLECTIC MEDICAL JOURNAL, CINCINNATI.

### OHIO COLLEGE OF DENTAL SURGERY

The Twenty-ninth Annual Session of the Ohio College of Dental Surgery will commence on the 15th of October next, and continue until March following.

### FACULTY.

JAMES TAYLOR, M. D., D. D. S.
Emeritus, Professor of Institutes of Dental Science.
WM. CLENDENIN, M. D.
Professor of Anatomy.
J. S. CASSIDY, D. D. S.
Professor of Chemistry and Therapeutics.
J. L. CILLEY, M. D.
Professor of Physiology and Histology.
F. BRUNNING, M. D.
Professor of Pathology and Therapeutics.
J. TAFT, D. D. S.
Professor of Operative Dentistry and Dental Hygiene.
Wm. VAN ANTWERP, D. D. S.
Professor of Mechanical Dentistry.
JAMES I. TAYLOR, JR., D. D. S.
Professor of Clinical Dentistry.
H. L. LEWIS, D. D. S.,
Demonstrator of Anatomy.

### TEXT BOOKS.

Gray's and Wilson's Anatomy, Dalton's or Carpenter's Phisiology; William's Pathology, Fowne's, Turner's, Graham's, or Brandt and Taylor's Chemistry; Taft's Operative Dentistry; Richardson's Mechanical Dentistry; Harris' Principles and Practice of Dental Science; Tomes' Dental Surgery; Watt's Chemical Essays; Harris' Dental Dictionary.

### TERMS OF ADMISSION.

Tickets must be procured at the beginning of the session.

| | |
|---|---|
| Tickets for the Course - - - - - - | $100.00 |
| Demonstrator's Ticket for Anatomy - - - | 5.00 |
| Matriculation Fee, but once - - - - | 5.00 |
| Diploma Fee - - - - - - - - | 30.00 |

### TERMS OF GRADUATION.

Two courses (including one of the dissections,) with a satisfactory dental pupilage. Eight years reputable practice, or a satisfactory examination upon the branches of the Junior Course in this Institu tion will be received as equal to one course.

An acceptable Thesis upon some subject in Dental Science.

Must possess a good moral character, and be twenty-one years old.

For further information, address J. TAFT, DEAN,

F. BRUNNING, M. D., Sec. 117 W. Fourth Street, Cincinnati, O

149 Broadway, Cincinnati, O. Jul-4

### PENNSYLVANIA COLLEGE OF DENTAL SURGERY,

**S. E. COR. ARCH & TENTH STS., PHILADELPHIA.**

THE NINETEENTH ANNUAL SESSION, 1874-'75.

### FACULTY:

T. L. BUCKINGHAM, D. D. S.,
*Professor of Chemistry.*
E. WILDMAN, M. D., D. D. S.,
*Professor of Mechanical Dentistry and Metallurgy.*
G. T. BAKER, D. D. S.,
*Professor of Dental Pathology and Therapeutics.*
JAMES TRUMAN, D. D. S.,
*Professor of Dental Histology and Operative Dentistry.*
JAMES TYSON, M. D.,
*Professor of Physiology and Microscopic Anatomy.*
J. EWING MEARS, M. D.,
*Professor of Anatomy and Surgery.*
E. R. PETTITT, D. D. S.,
*Demonstrator of Operative Dentistry.*
A. B. ABELL, JR., D. D. S.,
*Demonstrator of Mechanical Dentistry.*
J. L. SAXTON, D. D. S.,
*Assistant Demonstrator of Operative Dentistry.*
A. H HENDERSON, D. D. S.,
*Assistant Demonstrator of Mechanical Dentistry.*

### CLINICAL INSTRUCTORS:

A. LAWRENCE, M D., D. D. S.,

A. L. NORTHRUP, D. D. S.,

C. A. MARION, D. D. S.,

C. PALMER, D. D. S.,

E. T. DARBY, D. D. S.,

R. HUSY, D. D. S.,

W. H. TRUEMAN, D. D. S.

The College will be open for instruction during the entire year, except the last weeks in August and the first week in September. In the Dispensary and Laboratory, ample opportunities will be afforded the student for the prosecution of the practical parts of the profession, under the guidance and supervision of Demonstrators of known integrity and capacity. A Clinical Lecture will be given and operations performed by one of the Professors every Saturday afternoon during the sessions.

### *SPRING AND FALL SESSIONS.*

The Spring Course of Lectures will commence on the first Monday in April, and continue until the last of June. Fee for the Course $50 00.

The Fall Course will commence on the second Monday in September, and continue until the first of October, and will be free to those who matriculate for the Regular Session.

During the Spring and Fall Sessions there will be one Lecture a day, from 5 to 6 o'clock P. M., and with the exception of Saturday, the Dispensary and Laboratory will be open daily for six hours.

The attendance of the entire Spring and Fall Courses will be deemed equivalent to the term of pupilage under a private preceptor during the recess of the regular sessions.

### *THE REGULAR SESSION*

Will commence on the first Monday of November, and continue until the first of March ensuing, The course is so arranged that eighteen lectures will be delivered each week on the various branches taught in the College.

FEES.

| | |
|---|---|
| Matriculation, (paid but once) - - - - - - - - - - | $ 5 00 |
| For the Course, (Demonstrator's Ticket included) - - - - - - | 100 00 |
| Diploma, - - - - - - - - - - - - - - - - - - | 30 00 |

For further information, address,

**E. WILDMAN, Dean,**

**No. 1205 Arch Street, Philadelphia.**

Board can be obtained at from $5 00 to $8 00 per week.

All the Instruments and Tools required can be obtained for from $20 00 to $25 00.

jjason.

### OHIO COLLEGE OF DENTAL SURGERY.

The Twenty-ninth Annual Session of the Ohio College of Dental Surgery will commence on the 15th of October next, and continue until March following.

### FACULTY.

JAMES TAYLOR, M. D., D. D. S.
Emeritus, Professor of Institutes of Dental Science.
Wm. CLENDENIN, M. D.
Professor of Anatomy.
J. S. CASSIDY, D. D. S.
Professor of Chemistry and Therapeutics.
J. L. CILLEY, M. D.
Professor of Physiology and Histology.
F. BRUNNING, M. D.
Professor of Pathology and Therapeutics.
J. TAFT, D. D. S.
Professor of Operative Dentistry and Dental Hygiene.
Wm. VAN ANTWERP, D. D. S.
Professor of Mechanical Dentistry.
JAMES I. TAYLOR, Jr., D. D. S.
Professor of Clinical Dentistry.
H. L. LEWIS, D. D. S.,
Demonstrator of Anatomy.

### TEXT BOOKS.

Gray's and Wilson's Anatomy, Dalton's or Carpenter's Phisiology; William's Pathology, Fowne's, Turner's, Graham's, or Brandt and Taylor's Chemistry; Taft's Operative Dentistry; Richardson's Mechanical Dentistry; Harris' Principles and Practice of Dental Science; Tomes' Dental Surgery; Watt's Chemical Essays; Harris' Dental Dictionary.

### TERMS OF ADMISSION.

Tickets must be procured at the beginning of the session.

| | |
|---|---|
| Tickets for the Course - - - - - | $100.00 |
| Demonstrator's Ticket for Anatomy - - - | 5.00 |
| Matriculation Fee, but once - - - - | 5.00 |
| Diploma Fee - - - - - - - - | 30.00 |

### TERMS OF GRADUATION.

Two courses (including one of the dissections,) with a satisfactory dental pupilage. Eight years reputable practice, or a satisfactory examination upon the branches of the Junior Course in this Institution, will be received as equal to one course.

An acceptable Thesis upon some subject in Dental Science.

Must possess a good moral character, and be twenty-one years old.

For further information, address J. TAFT, Dean,
117 W. Fourth Street, Cincinnati, O.

F. BRUNNING, M. D., Sec.

149 Broadway, Cincinnati, O.

Jul-1

### PENNSYLVANIA COLLEGE OF DENTAL SURGERY,

**S. E. COR. ARCH & TENTH STS., PHILADELPHIA.**

THE NINETEENTH ANNUAL SESSION, 1874-'75.

### FACULTY:

T. L. BUCKINGHAM, D. D. S.,
*Professor of Chemistry.*
E. WILDMAN, M. D., D. D. S.,
*Professor of Mechanical Dentistry and Metallurgy.*
G. T. BAKER, D. D. S.,
*Professor of Dental Pathology and Therapeutics.*
JAMES TRUMAN, D. D. S.,
*Professor of Dental Histology and Operative Dentistry.*
JAMES TYSON, M. D.,
*Professor of Physiology and Microscopic Anatomy.*
J. EWING MEARS. M. D.,
*Professor of Anatomy and Surgery.*
E. R. PETTITT, D. D. S.,
*Demonstrator of Operative Dentistry.*
A. B. ABELL, JR., D. D. S.,
*Demonstrator of Mechanical Dentistry.*
J. L. SAXTON, D. D. S.,
*Assistant Demonstrator of Operative Dentistry.*
A. H HENDERSON, D. D. S.,
*Assistant Demonstrator of Mechanical Dentistry.*

### CLINICAL INSTRUCTORS:

A. LAWRENCE, M D., D. D. S.,

A. L. NORTHRUP, D. D. S.,

C. A. MARION, D. D. S.,

C. PALMER, D. D. S.,

E. T. DARBY, D. D. S.,

R. HUSY, D. D. S.,

W. H. TRUEMAN, D. D. S.

The College will be open for instruction during the entire year, except the last weeks in August and the first week in September. In the Dispensary and Laboratory, ample opportunities will be afforded the student for the prosecution of the practical parts of the profession, under the guidance and supervision of Demonstrators of known integrity and capacity. A Clinical Lecture will be given and operations performed by one of the Professors every Saturday afternoon during the sessions.

### *SPRING AND FALL SESSIONS.*

The Spring Course of Lectures will commence on the first Monday in April, and continue until the last of June. Fee for the Course $50 00.

The Fall Course will commence on the second Monday in September, and continue until the first of October, and will be free to those who matriculate for the Regular Session.

During the Spring and Fall Sessions there will be one Lecture a day, from 5 to 6 o'clock P. M.. and with the exception of Saturday, the Dispensary and Laboratory will be open daily for six hours.

The attendance of the entire Spring and Fall Courses will be deemed equivalent to the term of pupilage under a private preceptor during the recess of the regular sessions.

### *THE REGULAR SESSION*

Will commence on the first Monday of November, and continue until the first of March ensuing, The course is so arranged that eighteen lectures will be delivered each week on the various branches taught in the College.

FEES.

| | |
|---|---|
| Matriculation, (paid but once) | $ 5 00 |
| For the Course, (Demonstrator's Ticket included) | 100 00 |
| Diploma, | 30 00 |

For further information, address,

**E. WILDMAN, Dean,**

**No. 1205 Arch Street, Philadelphia.**

Board can be obtained at from $5 00 to $8 00 per week.

All the Instruments and Tools required can be obtained for from $20 00 to $25 00. jjason.

### PENNSYLVANIA COLLEGE OF DENTAL SURGERY,

**S. E. COR. ARCH & TENTH STS., PHILADELPHIA.**

THE NINETEENTH ANNUAL SESSION, 1874-'75.

### FACULTY:

T. L. BUCKINGHAM, D. D. S.,
*Professor of Chemistry.*
E. WILDMAN, M. D., D. D. S..
*Professor of Mechanical Dentistry and Metallurgy.*
G. T. BAKER, D. D. S.,
*Professor of Dental Pathology and Therapeutics.*
JAMES TRUMAN, D. D. S.,
*Professor of Dental Histology and Operative Dentistry.*
JAMES TYSON, M. D.,
*Professor of Physiology and Microscopic Anatomy.*
J. EWING MEARS. M. D.,
*Professor of Anatomy and Surgery.*
E. R. PETTITT, D. D. S.,
*Demonstrator of Operative Dentistry.*
A. B. ABELL, JR., D. D. S.,
*Demonstrator of Mechanical Dentistry.*
J. L. SAXTON, D. D. S.,
*Assistant Demonstrator of Operative Dentistry.*
A. H. HENDERSON, D. D. S.,
*Assistant Demonstrator of Mechanical Dentistry.*

### CLINICAL INSTRUCTORS:

A. LAWRENCE, M D., D. D. S., C. PALMER, D. D. S.,
A. L. NORTHRUP, D. D. S., E. T. DARBY, D. D. S.,
C. A. MARION, D. D. S., R. HUSY, D. D. S.,
W. H. TRUEMAN, D. D. S.

The College will be open for instruction during the entire year, except the last weeks in August and the first week in September. In the Dispensary and Laboratory, ample opportunities will be afforded the student for the prosecution of the practical parts of the profession, under the guidance and supervision of Demonstrators of known integrity and capacity. A Clinical Lecture will be given and operations performed by one of the Professors every Saturday afternoon during the sessions.

### *SPRING AND FALL SESSIONS.*

The Spring Course of Lectures will commence on the first Monday in April, and continue until the last of June. Fee for the Course $50 00.

The Fall Course will commence on the second Monday in September, and continue until the first of October, and will be free to those who matriculate for the Regular Session.

During the Spring and Fall Sessions there will be one Lecture a day, from 5 to 6 o'clock P. M.. and with the exception of Saturday, the Dispensary and Laboratory will be open daily for six hours.

The attendance of the entire Spring and Fall Courses will be deemed equivalent to the term of pupilage under a private preceptor during the recess of the regular sessions.

### *THE REGULAR SESSION*

Will commence on the first Monday of November, and continue until the first of March ensuing, The course is so arranged that eighteen lectures will be delivered each week on the various branches taught in the College.

**FEES.**

| | |
|---|---|
| Matriculation, (paid but once) - - - - - - - - - - | $ 5 00 |
| For the Course, (Demonstrator's Ticket included) - - - - - - | 100 00 |
| Diploma, - - - - - - - - - - - - - - - - - | 30 00 |

For further information, address,

**E. WILDMAN, Dean,**

**No. 1205 Arch Street, Philadelphia.**

Board can be obtained at from $5 00 to $8 00 per week.

All the Instruments and Tools required can be obtained for from $20 00 to $25 00. jjason.

### OHIO COLLEGE OF DENTAL SURGERY.

The Twenty-ninth Annual Session of the Ohio College of Dental Surgery will commence on the 15th of October next, and continue until March following.

### FACULTY.

JAMES TAYLOR, M. D., D. D. S.
Emeritus, Professor of Institutes of Dental Science.
WM. CLENDENIN, M. D.
Professor of Anatomy.
J. S. CASSIDY, D. D. S.
Professor of Chemistry and Therapeutics.
J. L. CILLEY, M. D.
Professor of Physiology and Histology.
F. BRUNNING, M. D.
Professor of Pathology and Therapeutics.
J. TAFT, D. D. S.
Professor of Operative Dentistry and Dental Hygiene.
Wm. VAN ANTWERP, D. D. S.
Professor of Mechanical Dentistry.
JAMES I. TAYLOR, JR., D. D. S.
Professor of Clinical Dentistry.
H. L. LEWIS, D. D. S.,
Demonstrator of Anatomy.

### TEXT BOOKS.

Gray's and Wilson's Anatomy, Dalton's or Carpenter's Phisiology; William's Pathology, Fowne's, Turner's, Graham's, or Brandt and Taylor's Chemistry; Taft's Operative Dentistry; Richardson's Mechanical Dentistry; Harris' Principles and Practice of Dental Science; Tomes' Dental Surgery; Watt's Chemical Essays; Harris' Dental Dictionary.

### TERMS OF ADMISSION.

Tickets must be procured at the beginning of the session.

| | |
|---|---|
| Tickets for the Course - - - - - | $100.00 |
| Demonstrator's Ticket for Anatomy - - - | 5.00 |
| Matriculation Fee, but once - - - - | 5.00 |
| Diploma Fee - - - - - - - - | 30.00 |

### TERMS OF GRADUATION.

Two courses (including one of the dissections,) with a satisfactory dental pupilage. Eight years reputable practice, or a satisfactory examination upon the branches of the Junior Course in this Institution, will be received as equal to one course.

An acceptable Thesis upon some subject in Dental Science.

Must possess a good moral character, and be twenty-one years old.

For further information, address J. TAFT, DEAN,
F. BRUNNING, M. D., Sec. 117 W. Fourth Street, Cincinnati, O.
149 Broadway, Cincinnati, O.

Jul-4

American Dental Association—Meets on the first Tuesday in Aug., 1874, at Detroit. *Pres.* T. L. Buckingham, Philad.; *Cor. Sec.* J. Taft, Cincinnati, O.; *Rec. Sec.* M. S. Dean, Chicago.

*American Dental Convention* Meets at Saratoga Springs, second Tuesday in Aug., 1874. *Pres.* John Allen, New York City; *Cor. Sec.* A. Tees, Philadelphia, Pa., *Rec. Sec.* C. L. Hurlbut, Springfield, Mass.

*List of Societies Represented in the American Dental Association.*

*American Academy of Dental Science* Meets at Boston, Mass. *Pres.* Joshua Tucker, M. D., of Boston; *Sec.* W. L. Tucker, of Boston; *Cor. Sec.*, E. N. Harris.

*Brooklyn Dental Society* Meets semi-monthly. *Pres.* W. T. Shannon,; *Rec. Sec.* C. P. Crandell; *Cor. Sec.* A. N. Chapman.

*Buffalo Dental Society* Meets second Monday of each month at Buffalo. *Pres.* S. A. Freeman; *Rec. Sec.* G. C. Daboll.

*Boston Dental Institute*—Meets first Tuesday of each month. *Pres.* D. G. Williams; *Cor. Sec.* D. S. Dickerman.

*California State Dental Association* Meets annually in June. *Pres.* C. C. Knowles, San Francisco; *Rec. Sec.* H. J. Plomteaux, Woodland.

*Cincinnati Dental Society*—Meets Semi-monthly at Cincinnati. *Pres.* H. R. Smith; *Rec. Sec.* D. C. Hawxhurst.

*Chicago Dental Society* Meets monthly at Chicago. *Pres.* M. S. Dean; *Rec. Sec.* Edgar D. Swain.

*Connecticut Valley Dental Association.* Meets semi-annually, second Tuesday of June and October. *Pres.* H. M. Miller, Westfield, Mass.; *Rec. Sec.* L. C. Taylor, of Holyoke, Mass.

*Connecticut State Dental Association.* *Pres.* R. C. Dunham, New Britain; *Rec. Sec.* D. E. Lane, Hartford.

*Charleston Dental Association*—Meets second Tuesday of every month; *Pres.* T. F. Chupein; *Sec. and Treas.* B. A. Muckenfuss.

*Delaware Dental Association*- -Meets semi-annually. *Pres.* E. W. Haines, Newark; *Sec.* C. R. Jeffries, Wilmington.

*Eastern Ohio Dental Association*- Meets on Tuesday, August 26th, 1873, at Alliance; *Pres.* A. J. Douds; *Sec.* J. M. Porter.

*East Tennessee Dental Association*—Meets annually at Knoxville, Tenn. *Pres.* Wm. W. F. Fowler; *Rec. Sec.* S. M. Prothro.

*Forest City Society of Dental Surgeons*- -*Pres.* B. Strickland, Cleveland, *Rec. Sec.* H. L. Ambler, Cleveland; *Cor. Sec.* Chas. Buffett, Cleveland.

*Georgia State Dental Society* Next meeting at Atlanta, July 1873. *Pres.* F. Y. Clark; *Cor. Sec.* J. P. H. Brown, Augusta.

*Harris Dental Association* Meets quarterly on the first Thursday in February, May. August, November. *Pres.* J. A. Martin, Strasburg; *Sec.* Wm. N. Amer, Lancaster,

*Hudson River Association of Dental Surgeons* Meets on the second Wednesdays of May, September and January. *Pres.* L. S. Straw, Newburg, N. Y; *Rec. Sec.* T. W. DuBois, Poughkeepsie, N. Y.

*Hudson Valley Dental Association* Meets monthly at Troy, N. Y. *Pres.* L. C. Wheeler, *Rec. Sec.* S. G. Andres; *Cor. Sec.* S. D. French.

*Illinois State Dental Society* Meets annually. *Pres.* C. Stoddard Smith, Springfield; *Sec.* C. R. E. Koch, Chicago. Meets second Tuesday in May, 1874, at Jacksonville.

*Indiana State Dental Society*—Meets next year at Indianapolis, on the first Tuesday of June. *Pres.* M. Wells, Indianapolis; *Cor. and Rec. Sec.* P. McDonald, Goshen.

*Iowa State Dental Society*- -Meets annually. Next meeting at Davenport, on the second Tuesday of May. *Pres.* L. C. Ingersoll, Keokuk. *Rec. Sec.* E. P. White, of Davenport; *Cor. Sec.* A. J. Redrich, Sioux City.

*Kansas State Dental Society* Meets semi-annually. Next meeting at Topeka, Sept. 12, 1873. *Pres.* Dr. Griswold; *Sec.* A. H. Thompson.

*Kansas and Missouri Valley Dental Association*—Meets first and third Tuesdays of erch month. *Pres.* J. R. Stark; *Sec.* H. C. Massie, Kansas City, Mo.

*Kentucky State Dental Association* Meets annually and semi-monthly, in the City of Louisville. Annnal meeting, first Tuesday in June, at 2 o'clock, P. M. Semi-monthly meetings, second and fourth Tuesday nights. G. W. Priest, *Pres.*; B. O. Doyle. *Sec.*, both at Louisville.

*Lake Erie Dental Association* Meets Annually. *Pres.* C. B. Ansart, Oil City, Pa. *Sec.* C. D. Elliot, Franklin, Pa. Next meeting first Tuesday of May, 1874.

*Mad River Dental Association* Meets at Dayton on the first Tuesdays in April and October. *Pres.* S. B. Tizzard, Dayton; *Sec.* A. J. Grosvenor, Troy.

*Maine Dental Society*—Meets in Aug., at Thomaston. *Pres.* E. J. Roberts, Augusta; *Sec.* C. E. Hussey, Biddeford.

*Maryland Association of Dentists* Meets the last Thursday of every month. *Pres.* J. B. Bean; *Sec.* S. M. Field.

*Massachusetts Dental Society*—Meets monthly. *Pres.* J. H. Batchelder, of Salem; *Sec.* C. Wilson; *Cor. Sec.* L. D. Shepard.

*Michigan State Dental Association*—Meets annually second Tuesday of Oct., at Ypsilanti. *Pres.* D. C. Hawxhurst, Ann Arbor; *Rec. & Cor. Sec.* T. R. Perry, Grand Rapids

*Michigan Central Dental Association.* Meets annually at Jackson, Secon Wednesdav of January, 1873. *Pres.* C. C. Jenkins, Anu Arbor, *Sec.* W. H. Darrance, Jackson.

*Mississippi Valley Dental Association* Meets annually at Cincinnati, on first Wednesday in March, 1874. *Pres.* S. B. Brown, Ft. Wayne Ind.; *Rec. Sec.* F. Hunter Cin.
*Minnesota Dental Associrtion* Meets annually. Next meeting at Faribault, last Wednesday in June, 1874. *Pres.* J. A. Bowman, Minneapolis; *Sec.* C. D. Montreville, St. Paul.
*Missouri Dental Association* Meets on the first Tuesday of June, in St. Louis. *Pres.* C. W. Rivers, St. Louis; *Sec.* W. H. Evans, St. Louis.
*Missouri Valley Dental Association** Meets on the second Tuesday in July and January. *Pres.* A. S. Billings, Omaha, Neb.; *Sec. and Treas.* J. F. Sanborn, Tabor, Iowa.
*Merrimac Valley Dental Association* Meets semi-annually first Tuesday of Oct. and May.* *Pres.* L. H. Kidder, Mass; *Rec. Sec.* A. M. Dudley, of Lowell; *Cor. Sec.* D. T. Porter, Lawrence, Mass.
*Memphis Dental Society*—Meets on the first Tuesday of each month. *Pres.* W. M. Arrington; *Sec.* V. F. Elliott; *Treas.* C. L. Harris.

*Nashville Dental Association*—Meets on the second Tuesday of each month. *Pres* J. C. Ross; *Sec.* R. R. Freeman; *Cor. Sec.* S. J. Cobb.
*New Jersey State Dental Society*—Meets annually. Next meeting at Mount Holly, on the second Tuesday in July, 1874. *Pres.* J. W. Cosad, Jersey City; *Sec.* J. W. Scarborough, Lambertville.
*New York State Dental Society*--Meets at Albany on the last Wednesday of June next. *Pres.* J. G. Ambler, New Yook; *Sec.* Chas. Barnes, of Syracuse, *Cor. Sec.* S. A. Freeman.
*New Haven Dental Society*--Meets on the first Wednesday of each month at New Haven. *Pres.* J. T. Metcalf, *Rec and Cor. Sec.* C. L. Smith, of New Haven.
*New Orleans Dental Society* Meets monthly. *Pres.*————————; *Rec. Sec.* A. F McLane.
*Northern Ohio Dental Association.* Meets annually, second Tuesday of June, 1873, at Put-in Bay. *Pres.* J. A. Robinson, Cleveland; *Rec. Sec.* A. F. Price, Fremont.
*Northern Iowa Dental Association*—Meets annually, on the second Tuesday in June, 1873. Next meeting at Waterloo; *Pres.* J. N. Abbott, Lancaster, *Rec. Sec.* A. V. Eaton, Anamosa; *Cor. Sec.* A. R. Mason, Waterloo.

*Ohio College Dental Association*--Meets annually Feb. 25. at Cincinnati. *Pres.* Jas. Leslie, Cin'ti.; *Rec. Sec.* H. A. Smith, of Cincinnati.
*Ohio State Dental Association*--Meets annually at Columbus. Next meeting, first Wednesday of December. *Pres.* H. A. Smith, Cincinnati; *Rec. Sec.* J. M. Porter, Massilon; *Cor. Sec.* C. R. Butler, Cleveland, O.
*Odontographic Society of Pennsylvania*--Meets monthly in Philadelphia. Next meeting, Wednesday, Sept. 1st. *Pres.* C. T. Stellwagen; *Rec. Sec.* A. Boice; *Cor. Sec.* E. L. Hewitt.
*Odontological Society of New York City* Meets the second Tuesday of each monh. *Pres.* C. E. Francis; *Rec. Sec.* W. E. Hong.
*Old Colony Dental Association* Meets quarterly.* *Pres.* C. G. Davis; *Rec. Sec.* L. W. Puffer, North Bridgewater, Mass; *Cor. Sec.* N. C. Fowler, Yarmouth, Mass.
*Ohio Valley Dental Association* Meets annually, in N. E. Ky; this year in Paris, Ky. Tuesday, Sept. 9th.
*Oregon State Dental Society* Meets annually. *Pres.* J. H. Hatch, Portland; *Rec. Sec.* J. R. Cardwell, Portland.

*Pennsylvania State Dental Society* Meets next at Wilkesbarre, second Tuesday of July, 1874. *Pres.* G. W. Klump, Williamsport; *Rec. Sec.* R. H. Moffit, Harrisburg *Cor. Sec.* S. Welchens, Lancaster.
*Poughkeepsie Dental Society* Meets monthly. *Pres.* J. H. Mann; *Sec.* H. F. Clark
*Pennsylvania Association of Dental Surgery* Meets monthly in Philadelphia. *Pres.* Spencer Roberts; *Sec.* E. R. Pettit.

*San Francisco Dental Association* Meets monthly. *Pres.* C. C. Knowles; *Cor. Sec.* H. E. Knox; *Rec. Sec.* Wm. Dutch.
*St. Louis Dental Society* Meets monthly. *Pres.* W. H. Morrison; *Sec.* A. H. Fuller.
*Susquehanna Dental Association* Meets annually.* *Pres.* J. L. Fordham: *Rec. Sec.* G. W. Klump, Williamsport. Meets Nov. 12, 1873.
*Society of Dental Surgeons of the City of New York* Meets semi-monthly in New York. *Pres.* B. F. Franklin: *Rec. Sec.* J. S. Latimer; *Cor. Sec.* E. H. Burras.
**Southern Dental Association* Meets annually. Next meeting the last Tuesday of July, 1873, at Baltimore, Md. *Pres.* H. M. Grant Virginia.: *Rec. Sec.* J. F. Thompson Va
*South Carolina State Dental Association* Meets semi-annually in May and Nov. *Pres.* W. C. Wardlaw, Abbeville: *Sec.* O. J. Bond, Marion.

*Tennessee Dental Association* Meets semi-annually. Next meeting in Nashville, on the 4th Wednesday in June. 1874. *Pres.* R. Russell, Nashville: *Sec.* A. Hartman, Murfreesboro; *Cor. Sec.* L. G. Noel, Nashville.
*Virginia State Dental Association* Meets in Richmond, first Thursday in Nov. of each year. *Pres.* H. M. Grant, Abington: *Sec.* G. F. Kersee, Richmond.

*Washington, City Dental Society* Meets annually. *Pres.* R. F. Hunt, *Sec.* Dr. Evans.
*Wabash Valley Dental Association* Meets annually. Next meeting at Wayne, third Tuesday in March, 1872. *Pres.* A. B. Cunningham, Attica: *Sec.* S. B. Brown, Fort Wayne.
* *Wisconsin State Dental Association* Meets semi-annually. Next meeting at Watertown, second Tuesday of January, 1872, *Pres.* Edgar Palmer, Le Crook *Sec.* C. C. Chittenden, Madison.

**Place of meeting fixed by appointment,*

### District Dental Societies of the State of New York.

*First District Dental Society* Meets Monday in New York. Annual meeting, second Wednesday in June: *Pres.* E. A. Bogue, New York: *Sec.* J. S. Latimer,. N. Y.

*Second District Dental Society.* Meets quarterly in Brooklyn. Annual meeting second Tuesday in April. *Pres.* W. S. Elliott: *Rec. Sec.* M. E. Elmandorf. Brooklyn: *Cor.* F. W. Dalbare.

*Third District Dental Society* Meets at Albany semi-annually and annually. *Pres.* D. D. French, Troy: *Sec.* S. J. Andress, Albany.

**Fourth District Dental Society* Meets annually in June at Saratoga. *Pres.* C, A. Elmore, Saratoga Springs. *Sec.* E. S. Pearsall, Fort Edwards.

**Fifth District Dental Society* Meets annually at Saratoga. *Pres.* W. W. Perkins *Sec.* F. D. Neelans.

**Sixth District Dental Society* Annual meeting at Binghampton, second Tuesday in June. *Pres.* R. Walker: *Rec. Sec.* P. L. Foote

**Seventh District Dental Society* Annual meeting is held the last Tuesday in June, at Rochester. *Pres.* G. W. Tripp, Auburn; *Sec.* J. E. Line, Rochester.

*Eighth District Dental Society* Meets annually on the second Tuesday in May, at Buffalo: *Bres.* J. C. Gifford, *Sec.* S. A. Freeman, Buffalo.

T*he Seventh and Eighth District Societies* Hold a union meeting on the first Tuesday of October, alternately at Buffalo and Rochester

*Place and time of meeting fixed by appointment.

---

The Ohio State Board of Dental Examiners will meet in Columbus on the first Tuesday of Dec. 1874, at 10 o'clock. *Pres.* J. Taft; *Sec.* W. P. Horton.

---

### List of Exchanges.

ST. LOUIS MEDICAL AND SURGICAL JOURNAL.
BOSTON MEDICAL AND SURGICAL JOURNAL.
THE PACIFIC MEDICAL AND SURGICAL JOURNAL, SAN FRANCISCO.
BUFFALO MEDICAL AND SURGICAL JOURNAL.
PHIDADELPHIA MEDICAL AND SURGICAL JOURNAL.
CINCINNATI LANCET AND OBSERVER.
DENTAL COSMOS, PHILADELPHIA,
BRAITHWAITE'S RETROSPECT, N. Y.
THE DENTAL TIMES, PHILADELPHIA.
LADIES' REPOSITORY, CINCINNATI.
HALL'S JOURNAL OF HEALTH, N. Y.
CHICAGO MEDICAL EXAMINER,
THE DETROIT REVIEW OF MEDICINE AND PHARMACY.
MEDICAL RECORD, N. Y.
NASHVILLE JOURNAL OF MEDICINE AND SURGERY.
AMERICAN ECLECTIC MEDICAL REVIEW. N. Y..
AMERICAN JOURNAL OF DENTAL SCIENCE, BALTIMORE.
THE UNITED STATES MEDICAL AND SURGICAL JOURNAL. CHICAGO
NEW ORLEANS JOURNAL OF MEDICINE,
WESTERN JOURNAL OF MEDICINE, INDIANAPOLIS, IND·
AMERICAN AGRICULTURIST, N. Y.
BOSTON JOURNAL OF CHEMISTRY.
JOURNAL OF APPLIED CHEMISTRY, N. Y.
DRUGGISTS' CIRCULAR AND CHEMICAL GAZETTE, N. Y.
CANADA JOURNAL OF DENTAL SCIENCE, MONTREAL.
INDIANA JOURNAL OF MEDICINE,
JOURNAL OF THE GYNÆCOLOGICAL SOCIETY, BOSTON.
MEDICAL AND SURGICAL REPORTER, SALEM, OREGON.
MISSOURI DENTAL JOURNAL, ST. LOUIS
ECLECTIC MEDICAL JOURNAL, CINCINNATI.

### PENNSYLVANIA COLLEGE OF DENTAL SURGERY,

**S. E. COR. ARCH & TENTH STS., PHILADELPHIA.**

THE NINETEENTH ANNUAL SESSION, 1874-'75.

**FACULTY:**

T. L. BUCKINGHAM, D. D. S.,
*Professor of Chemistry.*
E. WILDMAN, M. D., D. D. S.,
*Professor of Mechanical Dentistry and Metallurgy.*
G. T. BAKER, D. D. S.,
*Professor of Dental Pathology and Therapeutics.*
JAMES TRUMAN, D. D. S.,
*Professor of Dental Histology and Operative Dentistry,*
JAMES TYSON, M. D.,
*Professor of Physiology and Microscopic Anatomy.*
J. EWING MEARS. M. D.,
*Professor of Anatomy and Surgery.*
E. R. PETTITT, D. D. S.,
*Demonstrator of Operative Dentistry.*
A. B. ABELL, JR., D. D. S.,
*Demonstrator of Mechanical Dentistry.*
J. L. SAXTON, D. D. S.,
*Assistant Demonstrator of Operative Dentistry.*
A. H HENDERSON, D. D. S.,
*Assistant Demonstrator of Mechanical Dentistry.*

**CLINICAL INSTRUCTORS:**

A. LAWRENCE, M D., D. D. S., C. PALMER, D. D. S.,
A. L. NORTHRUP, D. D. S., E. T. DARBY, D. D. S.,
C. A. MARION, D. D. S., R. HUSY, D. D. S.,
W. H. TRUEMAN, D. D. S.

The College will be open for instruction during the entire year, except the last weeks in August and the first week in September. In the Dispensary and Laboratory, ample opportunities will be afforded the student for the prosecution of the practical parts of the profession, under the guidance and supervision of Demonstrators of known integrity and capacity. A Clinical Lecture will be given and operations performed by one of the Professors every Saturday afternoon during the sessions.

### *SPRING AND FALL SESSIONS.*

The Spring Course of Lectures will commence on the first Monday in April, and continue until the last of June. Fee for the Course $50 00.

The Fall Course will commence on the second Monday in September, and continue until the first of October, and will be free to those who matriculate for the Regular Session.

During the Spring and Fall Sessions there will be one Lecture a day, from 5 to 6 o'clock P. M., and with the exception of Saturday, the Dispensary and Laboratory will be open daily for six hours.

The attendance of the entire Spring and Fall Courses will be deemed equivalent to the term of pupilage under a private preceptor during the recess of the regular sessions.

### *THE REGULAR SESSION*

Will commence on the first Monday of November, and continue until the first of March ensuing. The course is so arranged that eighteen lectures will be delivered each week on the various branches taught in the College.

FEES.

| | |
|---|---|
| Matriculation, (paid but once) - - - - - - - - - | $ 5 00 |
| For the Course, (Demonstrator's Ticket included) - - - - - - | 100 00 |
| Diploma, - - - - - - - - - - - - - - - - - - | 30 00 |

For further information, address,

**E. WILDMAN, Dean,**

**No. 1205 Arch Street, Philadelphia.**

Board can be obtained at from $5 00 to $8 00 per week.

All the Instruments and Tools required can be obtained for from $20 00 to $25 00.

jjason.

AMERICAN DENTAL ASSOCIATION—Meets on the first Tuesday in Aug., 1874, at Detroit. *Pres.* T. L. Buckingham, Philad.; *Cor. Sec.* J. Taft, Cincinnati, O.; *Rec. Sec.* M. S. Dean, Chicago.

*American Dental Convention* Meets at Saratoga Springs, second Tuesday in Aug., 1874. *Pres.* John Allen, New York City; *Cor. Sec.* A. Tees, Philadelphia, Pa., *Rec. Sec.* C. L. Hurlbut, Springfield, Mass.

*List of Societies Represented in the American Dental Association.*

*American Academy of Dental Science* Meets at Boston, Mass. *Pres.* Joshua Tucker, M. D., of Boston; *Sec.* W. L. Tucker, of Boston; *Cor. Sec.*, E. N. Harris.

*Brooklyn Dental Society* Meets semi-monthly. *Pres.* W. T. Shannon,; *Rec. Sec.* C. P. Crandell; *Cor. Sec.* A. N. Chapman.

*Buffalo Dental Society* Meets second Monday of each month at Buffalo. *Pres.* S. A. FREEMAN; *Rec. Sec.* G. C. DABOLL.

*Boston Dental Institute*—Meets first Tuesday of each month. *Pres.* D. G. WILLIAMS; *Cor. Sec.* D. S. DICKERMAN.

*California State Dental Association* Meets annually in June. *Pres.* C. C. Knowles, San Francisco; *Rec. Sec.* H. J. Plomteaux, Woodland.

*Cincinnati Dental Society*—Meets Semi-monthly at Cincinnati. *Pres.* H. R. SMITH; *Rec. Sec.* D. C. HAWXHURST.

*Chicago Dental Society* Meets monthly at Chicago. *Pres.* M. S. Dean; *Rec. Sec.* Edgar D. Swain.

*Connecticut Valley Dental Association.* Meets semi-annually, second Tuesday of June and October. *Pres.* H. M. Miller, Westfield, Mass.; *Rec. Sec.* L. C. Taylor, of Holyoke, Mass.

*Connecticut State Dental Association.* *Pres.* R. C. Dunham, New Britain; *Rec. Sec.* D. E. Lane, Hartford.

*Charleston Dental Association*—Meets second Tuesday of every month; *Pres.* T. F. Chupein; *Sec. and Treas.* B. A. Muckenfuss.

*Delaware Dental Association*—Meets semi-annually. *Pres.* E. W. Haines, Newark; *Sec.* C. R. Jeffries, Wilmington.

*Eastern Ohio Dental Association*—Meets on Tuesday, August 26th, 1873, at Alliance; *Pres.* A. J. Douds; *Sec.* J. M. Porter.

*East Tennessee Dental Association*—Meets annually at Knoxville, Tenn. *Pres.* Wm. W. F. Fowler; *Rec. Sec.* S. M. Prothro.

*Forest City Society of Dental Surgeons*—*Pres.* B. Strickland, Cleveland, *Rec. Sec.* H. L. Ambler, Cleveland; *Cor. Sec.* Chas. Buffett, Cleveland.

*Georgia State Dental Society* Next meeting at Atlanta, July 1873. *Pres.* F. Y. Clark; *Cor. Sec.* J. P. H. Brown, Augusta.

*Harris Dental Association* Meets quarterly on the first Thursday in February, May, August, November. *Pres.* J. A. Martin, Strasburg; *Sec.* Wm. N. Amer, Lancaster.

*Hudson River Association of Dental Surgeons* Meets on the second Wednesdays of May, September and January. *Pres.* L. S. Straw, Newburg, N. Y; *Rec. Sec.* T. W. DuBois, Poughkeepsie, N. Y.

*Hudson Valley Dental Association* Meets monthly at Troy, N. Y. *Pres.* L. C. Wheeler, *Rec. Sec.* S. G. Andres; *Cor. Sec.* S. D. French.

*Illinois State Dental Society* Meets annually. *Pres.* C. Stoddard Smith, Springfield; *Sec.* C. R. E. Koch, Chicago. Meets second Tuesday in May, 1874, at Jacksonville.

*Indiana State Dental Society*—Meets next year at Indianapolis, on the first Tuesday of June. *Pres.* M. Wells, Indianapolis; *Cor. and Rec. Sec.* P. McDonald, Goshen.

*Iowa State Dental Society*—Meets annually. Next meeting at Davenport, on the second Tuesday of May. *Pres.* L. C. Ingersoll, Keokuk. *Rec. Sec.* E. P. White, of Davenport; *Cor. Sec.* A. J. Redrich, Sioux City.

*Kansas State Dental Society* Meets semi-annually. Next meeting at Topeka, Sept. 12, 1873. *Pres.* Dr. Griswold; *Sec.* A. H. Thompson.

*Kansas and Missouri Valley Dental Association*—Meets first and third Tuesdays of erch month. *Pres.* J. R. Stark; *Sec.* H. C. Massie, Kansas City, Mo.

*Kentucky State Dental Association* Meets annually and semi-monthly, in the City of Louisville. Annnal meeting, first Tuesday in June, at 2 o'clock, P. M. Semi-monthly meetings, second and fourth Tuesday nights. G. W. Priest, *Pres.*; B. O. Doyle. *Sec.*, both at Louisville.

*Lake Erie Dental Association* Meets Annually. *Pres.* C. B. Ansart, Oil City, Pa. *Sec.* C. D. Elliot, Franklin, Pa. Next meeting first Tuesday of May, 1874.

*Mad River Dental Association* Meets at Dayton on the first Tuesdays in April and October. *Pres.* G. W. Keely, Oxford; *Sec.* J. A Stipp, Sidney.

*Maine Dental Society*—Meets in Aug., at Thomaston. *Pres.* E. J. Roberts, Augusta; *Sec.* C. E. Hussey, Biddeford.

*Maryland Association of Dentists* Meets the last Thurs day of every month. *Pres.* J. B. Bean; *Sec.* S. M. Field.

*Massachusetts Dental Society*—Meets monthly. *Pres.* J. H. Batchelder, of Salem; *Sec.* C. Wilson; *Cor. Sec.* L. D. Shepard.

*Michigan State Dental Association*—Meets annually second Tuesday of Oct., at Ypsilanti. *Pres.* D. C. Hawxhurst, Ann Arbor; *Rec. & Cor. Sec.* T. R. Perry, Grand Rapids

*Michigan Central Dental Association.* Meets annually at Jackson, Secon Wednesday of January, 1873. *Pres.* C. C. Jenkins, Anu Arbor, *Sec.* W. H. Darrance, Jackson.

*Mississippi Valley Dental Association* Meets annually at Cincinnati, on first Wednesday in March, 1874. *Pres.* S. B. Brown, Ft. Wayne Ind.; *Rec. Sec.* F. Hunter Cin.

*Minnesota Dental Associrtion* Meets annually. Next meeting at Faribault, last Wed nesday in June, 1874. *Pres.* J. A. Bowman, Minneapolis; *Sec.* C. D. Montreville, St. Paul.

*Missouri Dental Association* Meets on the first Tuesday of June, in St. Louis. *Pres.* C. W. Rivers, St. Louis; *Sec.* W. H. Evans, St. Louis.

*Missouri Valley Dental Association** Meets on the second Tuesday in July and January. *Pres.* A. S. Billings, Omaha, Neb.; *Sec. and Treas.* J. F. Sanborn, Tabor, Iowa.

*Merrimac Valley Dental Association* Meets semi-annually first Tuesday of Oct. and May.* *Pres.* L. H. Kidder, Mass; *Rec. Sec.* A. M. Dudley, of Lowell; *Cor. Sec.* D. T. Porter, Lawrence, Mass.

*Memphis Dental Society*—Meets on the first Tuesday of each month. *Pres.* W. M. Arrington; *Sec.* V. F. Elliott; *Treas.* C. L. Harris.

*Nashville Dental Association*—Meets on the second Tuesday of each month. *Pres* J. C. Ross; *Sec.* R. R. Freeman; *Cor. Sec.* S. J. Cobb.

*New Jersey State Dental Society*—Meets annually. Next meeting at Mount Holly, on the second Tuesday in July, 1874. *Pres.* J. W. Cosad, Jersey City; *Sec.* J. W. Scarborough, Lambertville.

*New York State Dental Society*--Meets at Albany on the last Wednesday of June next. *Pres.* J. G. Ambler, New Yook; *Sec.* Chas. Barnes, of Syracuse, *Cor. Sec.* S. A. Freeman.

*New Haven Dental Society*--Meets on the first Wednesday of each month at New Haven. *Pres.* J. T. Metcalf, *Rec and Cor. Sec.* C. L. Smith, of New Haven.

*New Orleans Dental Society* Meets monthly. *Pres.* ——————; *Rec. Sec.* A. F McLane.

*Northern Ohio Dental Association.* Meets annually, second Tuesday of June, 1873, at Put-in Bay. *Pres.* J. A. Robinson, Cleveland; *Rec. Sec.* A. F. Price, Fremont.

*Northern Iowa Dental Association*—Meets annually, on the second Tuesday in June, 1873. Next meeting at Waterloo; *Pres.* J. N. Abbott, Lancaster, *Rec. Sec.* A. V. Eaton, Anamosa; *Cor. Sec.* A. R. Mason, Waterloo.

*Ohio College Dental Association*—Meets annually Feb. 25. at Cincinnati. *Pres.* Jas. Leslie, Cin'ti.; *Rec. Sec.* H. A. Smith, of Cincinnati.

*Ohio State Dental Association*—Meets annually at Columbus. Next meeting, first Wednesday of December. *Pres.* H. A. Smith, Cincinnati; *Rec. Sec.* J. M. Porter, Massilon; *Cor. Sec.* C. R. Butler, Cleveland, O.

*Odontographic Society of Pennsylvania*—Meets monthly in Philadelphia. Next meeting, Wednesday, Sept. 1st. *Pres.* C. T. Stellwagen; *Rec. Sec.* A. Boice; *Cor. Sec.* E. L. Hewitt.

*Odontological Society of New York City* Meets the second Tuesday of each monh. *Pres.* C. E. Francis; *Rec. Sec.* W. E. Hong.

*Old Colony Dental Association* Meets quarterly.* *Pres.* C. G. Davis; *Rec. Sec.* L. W. Puffer, North Bridgewater, Mass; *Cor. Sec.* N. C. Fowler, Yarmouth, Mass.

*Ohio Valley Dental Association* Meets annually, in N. E. Ky; this year in Paris, Ky. Tuesday, Sept. 9th.

*Oregon State Dental Society* Meets annually. *Pres.* J. H. Hatch, Portland; *Rec. Sec.* J. R. Cardwell, Portland.

*Pennsylvania State Dental Society* Meets next at Wilkesbarre, second Tuesday of July, 1874. *Pres.* G. W. Klump, Williamsport; *Rec. Sec.* R. H. Moffit, Harrisburg *Cor. Sec.* S. Welchens, Lancaster.

*Poughkeepsie Dental Society* Meets monthly. *Pres.* J H. Mann; *Sec.* H. F. Clark

*Pennsylvania Association of Dental Surgery* Meets monthly in Philadelphia. *Pres.* Spencer Roberts; *Sec.* E. R. Pettit.

*San Francisco Dental Association* Meets monthly. *Pres.* C. C. Knowles; *Cor. Sec.* H. E. Knox; *Rec. Sec.* Wm. Dutch.

*St. Louis Dental Society* Meets monthly. *Pres.* W. H. Morrison; *Sec.* A. H. Fuller.

*Susquehanna Dental Association* Meets annually.* *Pres.* J. L. Fordham: *Rec. Sec.* G. W. Klump, Williamsport. Meets Nov. 12, 1873.

*Society of Dental Surgeons of the City of New York* Meets semi-monthly in New York. *Pres.* B. F. Franklin: *Rec. Sec.* J. S. Latimer; *Cor. Sec.* E. H. Burras.

**Southern Dental Association* Meets annually. Next meeting the last Tuesday of July, 1873, at Baltimore, Md. *Pres.* H. M. Grant Virginia.: *Rec. Sec.* J. F. Thompson Va

*South Carolina State Dental Association* Meets semi-annually in May and Nov. *Pres.* W. C. Wardlaw, Abbeville: *Sec.* O. J. Bond, Marion.

*Tennessee Dental Association* Meets semi-annually. Next meeting in Nashville, on the 4th Wednesday in June. 1874. *Pres.* R. Russell, Nashville: *Sec.* A. Hartman, Murfreesboro; *Cor. Sec.* L. G. Noel, Nashville.

*Virginia State Dental Association* Meets in Richmond, first Thursday in Nov. of each year. *Pres.* H. M. Grant, Abington: *Sec.* G. F. Kersee, Richmond.

*Washington, City Dental Society* Meets annually. *Pres.* R. F. Hunt, *Sec.* Dr. Evans.

*Wabash Valley Dental Association* Meets annually. Next meeting at Wayne, third Tuesday in March, 1872. *Pres.* A. B. Cunningham, Attica: *Sec.* S. B. Brown, Fort Wayne.

* *Wisconsin State Dental Association* Meets semi-annually. Next meeting at Watertown. second Tuesday of January, 1872. *Pres.* Edgar Palmer, Le Crook *Sec.* C. C. Chittenden, Madison.

**Place of meeting fixed by appointment.*

### District Dental Societies of the State of New York.

*First District Dental Society* Meets Monday in New York. Annual meeting, second Wednesday in June : *Pres.* E. A. Bogue, New York : *Sec.* J. S. Latimer,. N. Y.

*Second District Dental Society.* Meets quarterly in Brooklyn. Annual meeting second Tuesday in April. *Pres.* W. S. Elliott : *Rec. Sec.* M. E. Elmandorf. Brooklyn : *Cor.* F. W. Dalbare.

*Third District Dental Society* Meets at Albany semi-annually and annually. *Pres.* D. D. French, Troy : *Sec.* S. J. Andress, Albany.

**Fourth District Dental Society* Meets annually in June at Saratoga. *Pres.* C, A. Elmore, Saratoga Springs. *Sec.* E. S. Pearsall, Fort Edwards.

**Fifth District Dental Society* Meets annually at Saratoga. *Pres.* W. W. Perkins *Sec.* F. D. Neelans.

**Sixth District Dental Society* Annual meeting at Binghampton, second Tuesday in June. *Pres.* R. Walker : *Rec. Sec.* P. L. Foote

**Seventh District Dental Society* Annual meeting is held the last Tuesday in June, at Rochester. *Pres.* G. W. Tripp, Auburn ; *Sec.* J. E. Line, Rochester.

*Eighth District Dental Society* Meets annually on the second Tuesday in May, at TBuffalo : *Bres.* J. C. Gifford, *Sec.* S. A. Freeman, Buffalo.

*he Seventh and Eighth District Societies* Hold a union meeting on the first Tues day of October, alternately at Buffalo and Rochester.

*Place and time of meeting fixed by appointment.

---

The Ohio State Board of Dental Examiners will meet in Columbus on the first Tuesday of Dec. 1874, at 10 o'clock. *Pres.* J. Taft; *Sec.* W. P. Horton.

---

### List of Exchanges.

ST. LOUIS MEDICAL AND SURGICAL JOURNAL.
BOSTON MEDICAL AND SURGICAL JOURNAL.
THE PACIFIC MEDICAL AND SURGICAL JOURNAL, SAN FRANCISCO.
BUFFALO MEDICAL AND SURGICAL JOURNAL.
PHIDADELPHIA MEDICAL AND SURGICAL JOURNAL.
CINCINNATI LANCET AND OBSERVER.
DENTAL COSMOS, PHILADELPHIA.
BRAITHWAITE'S RETROSPECT, N. Y.
THE DENTAL TIMES, PHILADELPHIA.
LADIES' REPOSITORY, CINCINNATI.
HALL'S JOURNAL OF HEALTH, N. Y.
CHICAGO MEDICAL EXAMINER.
THE DETROIT REVIEW OF MEDICINE AND PHARMACY.
MEDICAL RECORD, N. Y.
NASHVILLE JOURNAL OF MEDICINE AND SURGERY.
AMERICAN ECLECTIC MEDICAL REVIEW. N. Y..
AMERICAN JOURNAL OF DENTAL SCIENCE, BALTIMORE.
THE UNITED STATES MEDICAL AND SURGICAL JOURNAL, CHICAGO
NEW ORLEANS JOURNAL OF MEDICINE.
WESTERN JOURNAL OF MEDICINE, INDIANAPOLIS, IND.
AMERICAN AGRICULTURIST, N. Y.
BOSTON JOURNAL OF CHEMISTRY.
JOURNAL OF APPLIED CHEMISTRY, N. Y.
DRUGGISTS' CIRCULAR AND CHEMICAL GAZETTE, N. Y.
CANADA JOURNAL OF DENTAL SCIENCE, MONTREAL.
INDIANA JOURNAL OF MEDICINE.
JOURNAL OF THE GYNÆCOLOGICAL SOCIETY, BOSTON.
MEDICAL AND SURGICAL REPORTER, SALEM, OREGON.
MISSOURI DENTAL JOURNAL, ST. LOUIS
ECLECTIC MEDICAL JOURNAL, CINCINNATI.

AMERICAN DENTAL ASSOCIATION—Meets on the first Tuesday in Aug., 1875, at Niagara Falls. *Pres.* M. S. Dean, Chicago; *Cor. Sec.* Geo. L, Filed, Detroit; *Rec. Sec.* C. Stodard Smith, Springfield, Ill.

*American Dental Convention* Meets at Long Branch, second Tuesday in August, 1875. *Pres.* B. F. Coy, Baltimore: *Cor. Sec.* D. Roberts, Philadelphia, Pa.; *Rec. Sec.* A. Tees, Philadelphia, Pa.

*List of Societies Represented in the American Dental Association.*

*American Academy of Dental Science* Meets at Boston, Mass. *Pres.* Joshua Tucker M. D., of Boston ; *Sec.* W. L. Tucker, of Boston ; *Cor. Sec.*, E. N. Harris.

*Brooklyn Dental Society* Meets semi-monthly. *Pres.* W. T. Shannon, ; *Rec. Sec.* C. P. Crandell ; *Cor. Sec.* A. N. Chapman.

*Buffalo Dental Society* Meets second Monday of each month at Buffalo. *Pres.* S. A. FREEMAN ; *Rec. Sec.* G. C. DABOLL.

*Boston Dental Institute*—Meets first Tuesday of each month. *Pres.* D. G. WILLIAMS, Buffalo; *Cor. Sec.* D. S. Dickerman.

*California State Dental Association* Meets annually in June. *Pres.* C. C. Knowles, San Francisco ; *Rec. Sec.* H. J. Plomteaux, Woodland.

*Cincinnati Dental Society*—Meets Semi-monthly at Cincinnati. *Pres.* H. R. SMITH ; *Rec. Sec.* D. C. HAWXHURST.

*Chicago Dental Society* Meets monthly at Chicago. *Pres.* M. S. Dean ; *Rec. Sec.* Edgar D. Swain.

*Connecticut Valley Dental Association.* Meets semi-annually, second Tuesday of June and October. *Pres.* H. M. Miller, Westfield, Mass.; *Rec. Sec.* L. C. Taylor, of Holyoke, Mass.

*Connecticut State Dental Association.* *Pres.* R. C. Dunham, New Britain ; *Rec. Sec.* D. E. Lane, Hartford.

*Charleston Dental Association*—Meets second Tuesday of every month ; *Pres.* T. F. Chupein ; *Sec. and Treas.* B. A. Muckenfuss.

*Delaware Dental Association*—Meets semi-annually. *Pres.* E. W. Haines, Newark ; *Sec.* C. R. Jeffries, Wilmington.

*Eastern Indiana Dental Association.*—Meets semi-annually, first Tuesday in May and last Tuesday in October; *Pres.* J. K. Jameson, Connersville; *Sec.* J. W. White, Knightstown, Ind. (Place of meeting fixed by appointment.)

*Eastern Ohio Dental Association*—Meets on Tuesday, August 26th, 1873, at Alliance ; *Pres.* A. J. Douds ; *Sec.* J. M. Porter.

*East Tennessee Dental Association*—Meets annually at Knoxville, Tenn. *Pres.* Wm. W. F. Fowler; *Rec. Sec.* S. M. Prothro.

*Forest City Society of Dental Surgeons*—*Pres.* B. Strickland, Cleveland, *Rec. Sec.* H. L. Ambler, Cleveland ; *Cor. Sec.* Chas. Buffett, Cleveland.

*Georgia State Dental Society* Next meeting at Atlanta, July 1873. *Pres.* F. Y. Clark ; *Cor. Sec.* J. P. H. Brown, Augusta.

*Harris Dental Association* Meets quarterly on the first Thursday in February, May. August, November. *Pres.* J. A. Martin, Strasburg ; *Sec.* Wm. N. Amer, Lancaster.

*Hudson River Association of Dental Surgeons* Meets on the second Wednesdays of May, September and January. *Pres.* L. S. Straw, Newburg, N. Y ; *Rec. Sec.* T. W. DuBois, Poughkeepsie, N. Y.

*Hudson Valley Dental Association* Meets monthly at Troy, N. Y. *Pres.* L. C. Wheeler, *Rec. Sec.* S. G. Andres ; *Cor. Sec.* S. D. French.

*Illinois State Dental Society* Meets annually. *Pres.* G. S. Miles, Jerseyville, *Sec.* C. R. E. Koch, Chicago. Meets second Tuesday in May, 1875, at Ottowa.

*Indiana State Dental Society*—Meets next year at Indianapolis, on the first Tuesday of June. *Pres.* M. Wells, Indianapolis ; *Cor. and Rec. Sec.* P. McDonald, Goshen.

*Iowa State Dental Society*—Meets annually. Next meeting at Davenport, on the second Tuesday of May. *Pres.* L. C. Ingersoll, Keokuk. *Rec. Sec.* E. P. White, of Davenport ; *Cor. Sec.* A. J. Redrich, Sioux City.

*Kansas State Dental Society* Meets annually. Next meeting at Leavanworth, first Wednesday in May, 1875. *Pres.* L. C. Wasson; *Sec.* J. D Patterson.

*Kansas and Missouri Valley Dental Association*—Meets first and third Tuesdays of erch month. *Pres.* J. R.Stark ; *Sec.* H. C. Massie, Kansas City, Mo.

*Kentucky State Dental Association* Meets annually first Tuesday in June, next meeting in Lexington. *Pres.*, B. O. Doyle; *Sec.*, Chas. E Dunn; both of Louisville.

*Lake Erie Dental Association* Meets Annually. *Pres.* C. B. Ansart, Oil City, Pa. *Sec.* C. D. Elliot, Franklin, Pa. Next meeting first Tuesday of May, 1874.

*Mad River Dental Association* Meets at Dayton on the first Tuesdays in April and October. *Pres.* S. B. Tizzard, Dayton ; *Sec.* A. J. Grosvenor, Troy.

*Maine Dental Society*—Meets in Aug., at Thomaston. *Pres.* E. J. Roberts, Augusta ; *Sec.* C. E. Hussey, Biddeford.

*Maryland Association of Dentists* Meets the last Thursday of every month. *Pres.* J. B. Bean ; *Sec.* S. M. Field.

*Massachusetts Dental Society*—Meets monthly. *Pres.* J. H. Batchelder, of Salem *Sec.* C. Wilson ; *Cor. Sec.* L. D. Shepard.

*Michigan State Dental Association*—Meets annually second Tuesday of Oct., at Ypsilanti. *Pres.* D. C. Hawxhurst, Ann Arbor; *Rec. & Cor. Sec.* T. R. Perry, Grand Rapids

*Michigan Central Dental Association.* Meets annually at Jackson, Secon Wednesday of January, 1873. *Pres.* C. C. Jenkins, Anu Arbor , *Sec.* W. H. Darrance, Jackson.

*Mississippi Valley Dental Association* Meets annually at Cincinnati, on first Wednesday in March, 1874. *Pres.* S. B. Brown, Ft. Wayne Ind.; *Rec. Sec.* F. Hunter Cin.

*Minnesota Dental Associrtion* Meets annually. Next meeting at Faribault, last Wednesday in June, 1874. *Pres.* J. A. Bowman, Minneapolis; *Sec.* C. D. Montreville, St. Paul.

*Missouri Dental Association* Meets on the first Tuesday of June, in St. Louis. *Pres.* C. W. Rivers, St. Louis; *Sec.* W. H. Evans, St. Louis.

*Missouri Valley Dental Association** Meets on the fourth Tuesday in July, in Nebraska City. *Pres.* A. S. Billings, Omaha, Neb.; *Sec. and Treas.* J. F. Sanborn, Tabor, Iowa.

*Merrimac Valley Dental Association* Meets semi-annually first Tuesday of Oct. and May.* *Pres.* L. H. Kidder, Mass; *Rec. Sec.* A. M. Dudley, of Lowell; *Cor. Sec.* D. T. Porter, Lawrence, Mass.

*Memphis Dental Society*—Meets on the first Tuesday of each month. *Pres.* W. M. Arrington; *Sec.* V. F. Elliott; *Treas.* C. L. Harris.

*Nashville Dental ssociation*—Meets on the second Tuesday of each month. *Pres.* J. C. Ross; *Sec.* R. R. Freeman; *Cor. Sec.* S. J. Cobb.

*New Jersey State Dental Society*—Meets annually. Next meeting at Long Branch, on the first Tuesday in July, 1875. *Pres.* Geo. C. Brown, Mount Holly; *Sec.* J. W. Scarborough, Lambertville.

*New York State Dental Society*--Meets at Albany on the last Wednesday of June next. *Pres.* W. C. Barret, Warsaw, N. Y.; *Sec.* Chas. Barnes, of Syracuse, *Cor. Sec.* W. S. Elliott, Goshen; *Rec. Sec.* Charles Barnes, Syracuse,

*New Haven Dental Society*--Meets on the first Wednesday of each month at New Haven. *Pres.* J. T. Metcalf, *Rec and Cor. Sec.* C. L. Smith, of New Haven.

*New Orleans Dental Society* Meets monthly. *Pres.* ——————; *Rec. Sec.* A. F McLane.

*Northern Ohio Dental Association.* Meets annually, second Tuesday of June, 1873, at Put-in Bay. *Pres.* J. A. Robinson, Cleveland; *Rec. Sec.* A. F. Price, Fremont.

*Northern Iowa Dental Association*—Meets annually, on the second Tuesday in June, 1873. Next meeting at Waterloo; *Pres.* J. N. Abbott, Lancaster, *Rec. Sec.* A. V. Eaton, Anamosa; *Cor. Sec.* A. R. Mason, Waterloo.

*Ohio College Dental Association*—Meets annually Feb. 25. at Cincinnati. *Pres.* Jas. Leslie, Cin'ti.; *Rec. Sec.* H. A. Smith, of Cincinnati.

*Ohio State Dental Association*—Meets annually at Columbus. Next meeting, first Wednesday of December. *Pres.* H. A. Smith, Cincinnati; *Rec. Sec.* J. M. Porter, Massilon; *Cor. Sec.* C. R. Butler, Cleveland, O.

*Odontographic Society of Pennsylvania*—Meets monthly in Philadelphia. Next meeting, Wednesday, Sept. 1st. *Pres.* C. T. Stellwagen; *Rec. Sec.* E. L. Hewitt.

*Odontological Society of New York City* Meets the second Tuesday of each monh. *Pres.* C. E. Francis; *Rec. Sec.* W. E. Hong.

*Old Colony Dental Association* Meets quarterly.* *Pres.* C. G. Davis; *Rec. Sec.* L. W. Puffer, North Bridgewater, Mass; *Cor. Sec.* N. C. Fowler, Yarmouth, Mass.

*Ohio Valley Dental Association* Meets annually, in N. E. Ky; this year in Paris, Ky. Tuesday, Sept. 9th.

*Oregon State Dental Society* Meets annually. *Pres.* J. H. Hatch, Portland; *Rec. Sec.* J. R. Cardwell, Portland.

*Pennsylvania State Dental Society* Meets next at Wilkesbarre, second Tuesday of July, 1874. *Pres.* G. W. Klump, Williamsport; *Rec. Sec.* R. H. Moffit, Harrisburg *Cor. Sec.* S. Welchens, Lancaster.

*Poughkeepsie Dental Society* Meets monthly. *Pres.* J. H. Mann; *Sec.* H. F. Clark

*Pennsylvania Association of Dental Surgery* Meets monthly in Philadelphia. *Pres.* Spencer Roberts; *Sec.* E. R. Pettit.

*San Francisco Dental Association* Meets monthly. *Pres.* C. C. Knowles; *Cor. Sec.* H. E. Knox; *Rec. Sec.* Wm. Dutch.

*St. Louis Dental Society* Meets monthly. *Pres.* W. H. Morrison; *Sec.* A. H. Fuller.

*Susquehanna Dental Association* Meets annually.* *Pres.* J. L. Fordham: *Rec. Sec.* Wm. Nertz. Meets Nov. 11, 1874, at Lewisburg.

*Society of Dental Surgeons of the City of New York* Meets semi-monthly in New York. *Pres.* B. F. Franklin: *Rec. Sec.* J. S. Latimer; *Cor. Sec.* E. H. Burras.

**Southern Dental Association* Meets annually. Next meeting at Memphis, Tenn., at time of Mardi-Gras. *Pres.* J. R. Walker, New Orleans, La.; *Rec. Sec.* J. F Thompson Va.

*South Carolina State Dental Association* Meets semi-annually in May and Nov. *Pres.* T. F. Chupein, Charleston; *Sec.* J. W. Norwood, Greenville; *Cor. Sec.* C. C. Patrick, Charleston, S. C. Next meeting at Columbia.

*Tennessee Dental Association* Meets semi-annually. Next meeting in Nashville, on the 4th Wednesday in June. 1875. *Pres.* E. S. Chisholm; *Sec.* J. S. King; *Cor. Sec.* S. J. Cobb.

*Virginia State Dental Association* Meets in Richmond, first Thursday in Nov. of each year. *Pres.* H. M. Grant, Abington: *Sec.* G. F. Kersee, Richmond.

*Washington, City Dental Society* Meets annually. *Pres.* R. F. Hunt, *Sec.* Dr. Evans.

*Wabash Valley Dental Association* Meets annually. Next meeting at Wayne, third Tuesday in March, 1872. *Pres.* A. B. Cunningham, Attica: *Sec.* S. B. Brown, Fort Wayne.

**Wisconsin State Dental Association* Meets semi-annually. Next meeting at Watertown. second Tuesday of January, 1872, *Pres.* Edgar Palmer, Le Crook *Sec.* C. C. Chittenden, Madison.

**Place of meeting fixed by appointment*

### District Dental Societies of the State of New York.

*First District* Meets second and fourth Wednesday evenings of each month. Annual meeting in New York, first Tuesday in June. *Pres*. R. M, Gage; *Rec. Sec.* W. E. Hoag, 331 Madison ave., New York.
*Second District.* Annual meeting in Brooklyn, first Monday in April. *Pres.* W. S. Elliott; *Rec. Sec.* M. E. Elmandorf. Brooklyn; *Cor. Sec.* F. W. Dalbare.
*Third District.* Meets semi-annually on second Tuesday in January and July. Annual meeting at Albany on second Tuesday in January. *Pres.* J. C. Austin, Albany; *Rec. Sec.* S. J. Andress, Troy; *Cor. Sec.* D. H. Young, Troy.
*Fourth District.* Meets semi-annually. Meets annually at Saratoga on second Tuesday in June. *Pres.* C, A. Elmore, Ft. Edward; *Rec. Sec.* E. S. Pearsall, Saratoga.
*Fifth District.* Meets semi-annually. Annual meeting at Syracuse. second Tuesday in June. *Pres.* J. D. Huntington, Watertown; *Rec. Sec.* F. D. Nellis, Syracuse; *Cor. Sec.* J. S. Marshall, Syracuse.
*Sixth District.* Meets semi-annually. Annual meeting at Binghampton on second Tuesday in June. *Pres.* Frank B. Darby, Oswego; *Rec. Sec.* F. E. Perry, Norwich; *Cor. Sec.* A. M. Holmes, Morrisville.
*Seventh District.* Meets semi-annually. Annual meeting at Rochester first Tuesday in June. *Pres.* H. S. Miller; *Rec. Sec.* J. E. Line, Rochester.
*Eighth District.* Meets semi-annually. Annual on second Tuesday in May. *Pres* G. C. Daboll, *Rec. Sec.* S. A. Freeman, *Cor. Sec.* T. G. Lewis, Buffalo.
*Buffalo Dental Association.* Meets on second Tuesday evening of each month. Annual meeting in June. *Pres.* S. A. Freeman; *Sec.* W. H. Barrows.

The Ohio State Board of Dental Examiners will meet in Columbus on the first Tuesday of Dec. 1874, at 10 o'clock. *Pres.* J. Taft; *Sec.* W. P. Horton.

### List of Exchanges.

### THE POCKET

### DENTAL REGISTER.

Every dentist with anything like a full practice, and who cares to conduct business in a systematic manner, must have a book in which to record his dental operations.

The large "Registers" that have been used for that purpose, are excellent in their place, and may still be used in connection with such a book as this, if desired; but they are open to several important objections.

Many dentists using them have doubtless experienced the inconvenience of going to the desk after each patient's sitting, to make a record, and have preferred trusting to memory till after office hours. By that time, the exact tooth operated upon has often been forgotten; and sometimes a certain operation has slipped from the mind altogether.

The fillings put in to-day are marked on the same diagram with those inserted a month or a year ago; consequently, after a little while, it is impossible to distinguish between old and recent work.

The space reserved for each account being so large, one is unwilling to spoil a blank to record trifling services, such as extracting or treating a tooth for a patient whom he may never see again; although it often happens afterward that he would be glad to have a knowledge of the service.

In recording operations on the temporary teeth, it is very unsatisfactory to cross out the twelve back teeth of the permanent set, and imagine the bicuspids to be temporary molars. In such cases, much confusion generally follows in the course of a few years.

This POCKET REGISTER is presented with the belief that it is free from these objectionable features.

Each operation may be recorded with pen or pencil, right at the chair, in almost the same time that an appointment is made for another sitting.

Every operation bears its own date, and cannot be taken for a previous or later one.

The space for accounts is so economically used, that one will not hesitate to record the most trivial services. It consists of 224 pages, arranged as follows:

120 pages for small account , ten to the page, with blanks for date, name, residence, operation, debit, and credit. Each account is also furnished with a diagram of the teeth for marking operations performed.
24 pages for medium accounts, two to the page, with blanks and diagram, as above.
16 pages for large accounts, one to the page, with blanks and diagram.
16 pages with blanks and diagrams expressly arranged for operations on the temporary teeth, ten accounts to the page.
32 pages for appointments for 64 weeks. These are without date, and may, therefore, be used for any time,
16 pages for index and memoranda.

It will be seen that the "Pocket Register" combines Account-book, Record of Operations on the Permanent and Temporary Teeth, Appointment-book, and Memoranda; and that the whole is offered for about the price of an ordinary Engagement book.

It is printed on fine writing paper, bound handsomely in leather, with tuck, or in English cloth, and is of a suitable size for carrying in the breast pocket.

— PRICE. —

| | |
|---|---|
| *In Leather, with Tuck.* | $1 50 |
| *In English Cloth,* | 1 25 |

Sent post-paid, on receipt of price. For sale, wholesale and retail, by

**For Sale by SPENCER, CROCKER & CO.**

### CONTENTS.

### COMMUNICATIONS:

### EDITORIAL:

Success , . . . . . . . . . . 523
Failures . . . . . . . . . . 525
Dental Vulcanite Controversy . . . . . . 526
Ohio Dental College Laboratory . . . . . 528
For Sensitive Dentine . . . . . . . . 530
Spongoid . . . . . . . . . . . 531
Notice . . . . . . . . . . . . 447
The Physician's Visiting List . . . . . . . 532

### THE DENTAL REGISTER,

### A Monthly Magazine devoted entirely to the Interest of

### THE DENTAL PROFESSION.

**Terms Reduced to $2 50 per Year. Single Numbers 25cts.**

**EDITED BY PROF. J. TAFT,**

**And Published by SPENCER, CROCKER & CO., Ohio Dental Depot.**

The volume commences in January and closes in December.

The REGISTER being issued monthly is always fresh, therefore indispensable to the practitioner who desires to keep posted up with the new inventions and improvements which are daily developed. Neither expense nor effort will be spared to give value and variety to its contents. Articles will be illustrated with well executed engravings whenever they will add to the interest or assist in explanation.

Its extensive circulation and frequency of issue make it one of the BEST DENTAL ADVERTISING MEDIUMS in the United States.

All subscriptions must begin with January or July.

**RATES OF ADVERTISING.**

| | 1 Mo. | 3 Mo. | 6 Mo. | 1 Year. | | 1 Year. | |
|---|---|---|---|---|---|---|---|
| 1 page.... | $12 00 | $25 00 | $40 00 | $75 00 | Inserted pages must | 1st page. | $50 00 |
| ½ page ... | 8 00 | 15 00 | 25 00 | 40 00 | be plain paper and | 2d page. | 37 50 |
| ¼ page ... | 5 00 | 10 00 | 15 00 | 25 00 | black ink. | 3d page. | 33 33 |
| | | | | | | 4th page. | 25 00 |

Local or special notices, five lines or less, will be inserted for $3 each insertion: over five lines, 50 cts. per line

All advertising bills payable in advance, or at furthest, after the issue of the first number containing the advertisement. All transient advertisements to be paid for in advance.

☞CONTRIBUTIONS SOLICITED.

Exclusively Editorial Communications should be addressed to

**DR. J. TAFT, EDITOR.**

The latest number of the REGISTER may be ordered with or without other goods at any time, and all letters should be addressed to

**SPENCER, CROCKER & CO., OHIO DENTAL DEPOT,**

Cincinnati, Ohio.

